# Development of Novel Proline- and Pipecolic Acid-Based Allosteric Inhibitors of Dengue and Zika Virus NS2B/NS3 Protease

**DOI:** 10.3390/ph19010024

**Published:** 2025-12-22

**Authors:** Josè Starvaggi, Carla Di Chio, Johannes Lang, Valentina Belgiovine, Daniela Trisciuzzi, Santo Previti, Christian Klein, Orazio Nicolotti, Salvatore Di Maro, Maria Zappalà, Roberta Ettari

**Affiliations:** 1 Department of Chemical, Biological, Pharmaceutical and Environmental Sciences, University of Messina, Viale Ferdinando Stagno D’Alcontres 31, 98166 Messina, Italy; jose.starvaggi@studenti.unime.it (J.S.); cdichio@unime.it (C.D.C.); spreviti@unime.it (S.P.); mzappala@unime.it (M.Z.); 2 Institute of Pharmacy and Molecular Biotecnology, University of Heidelberg, Im Neuenheimer Feld 364, 69120 Heidelberg, Germany; johannes.lang@uni-heidelberg.de (J.L.); c.klein@uni-heidelberg.de (C.K.); 3 Department of Pharmacy-Drug Science, University of Bari, Via Amendola 173, 70126 Bary, Italy; valentina.belgiovine@uniba.it (V.B.); daniela.trisciuzzi@uniba.it (D.T.); orazio.nicolotti@uniba.it (O.N.); 4 Department of Environmental, Biological and Pharmaceutical Science and Technology, University of Campania “Luigi Vanvitelli”, Via A. Vivaldi, 43, 81100 Caserta, Italy; salvatore.dimaro@unicampania.it

**Keywords:** allosteric inhibitors, dengue virus, Zika virus, NS2B/NS3 serine protease

## Abstract

**Background:** In this study, we report a novel series of proline- and pipecolic acid-based small molecules designed as allosteric inhibitors of the NS2B/NS3 serine proteases from dengue and Zika viruses, key targets in antiviral drug discovery. **Results:** Enzymatic studies revealed that *S*-proline derivatives bearing electron-withdrawing substituents on the aromatic ring, particularly that with a trifluoromethyl group in *meta* position (i.e., compound **3**, IC_50_ = 5.0 µM), were the most potent against DENV NS2B/NS3, while nitro-substituted inhibitors were mostly effective only against the ZIKV protease. *R*-configured pipecolic acid-based derivatives were the only ones active against DENV NS2B/NS3, even if the mid-micromolar range; however, they demonstrated improved cellular efficacy since inhibitors **24** and **27** exhibiting strong activity in a DENV2 protease reporter gene assay (EC_50_ = 5.2 and 5.1 µM, respectively). All compounds showed no cytotoxicity (CC_50_ > 100 µM) and were selective for the viral protease over off-target serine proteases. Structure-based approaches were exploited to map the druggable allosteric site close to Asn152. **Conclusions:** Our findings led us to identify proline and pipecolic acid-based inhibitors as promising leads for the development of selective flaviviral NS2B/NS3 allosteric inhibitors.

## 1. Introduction

Since the beginning of 2025, the World Health Organization (WHO) has reported over three million dengue cases and over 1400 dengue-related deaths from 90 regions in the Pan American Health Organization (PAHO), South-East Asia and West Pacific Regions (SEARO and WPRO, respectively), in the Eastern Mediterranean Region (EMRO) and in Africa. In mainland Europe, there have been no locally acquired cases in 2025; however, cases have been reported from the EU’s outermost regions [1]. Dengue is a viral infection transmitted by mosquitoes and prevalent in tropical regions. It is caused by one of four closely related viruses, known as dengue virus (DENV) serotypes.

In 2023, dengue was designated a Grade 3 emergency by the WHO after outbreaks escalated in multiple countries [2].

Zika virus (ZIKV) is a mosquito-transmitted virus that was first identified in a Rhesus macaque in Uganda in 1947, with subsequent reports of human infection and disease in other African countries during the 1950s. Since 2007, outbreaks of ZIKV have been documented across Africa, the Americas, Asia and the Pacific. Although global cases of ZIKV have declined since 2017, transmission continues at low levels in several countries in the Americas and other endemic regions.

Additionally, the first locally transmitted mosquito-borne cases of ZIKV were reported in Europe in 2019, and outbreak activity was observed in India in 2021. To date, 89 countries and territories have reported evidence of mosquito-borne ZIKV infection; however, global surveillance remains limited [3]. DENVs are typically transmitted to humans through bites from infected *Aedes* mosquitoes [4]. ZIKV is primarily spread by *Aedes aegypti* and *Aedes albopictus*, which are also commonly associated with the flavivirus distribution, though human-to-human transmission has also been documented [5,6,7,8]. Classic dengue fever, often referred to as “breakbone fever,” manifests as an acute high fever 3–8 days after the bite of an infected mosquito.

Symptoms of dengue fever include frontal headache, retro-orbital pain, myalgia, arthralgia, rash and leukopenia. In some cases, dengue can progress to dengue hemorrhagic fever (DHF), a severe and potentially fatal form of the disease characterized by rapid temperature changes (from fever to hypothermia), hemorrhagic manifestations and alterations in mental status, such as irritability, confusion or obtundation. Without proper management, DHF can be fatal [9]. ZIKV infection during pregnancy can lead to microcephaly and other congenital abnormalities in the infant, including limb contractures, hypertonia, ocular defects and hearing loss. In addition, ZIKV infection has been associated with neurological complications such as Guillain–Barré syndrome, neuropathy and myelitis, particularly in adults [10].

Currently, TAK-003 is the only licensed dengue vaccine. It is administered as a two-dose series, spaced three months apart, and is recommended for specific age groups and circumstances in accordance with WHO guidelines. The WHO advises the use of TAK-003 in children aged 6–16 years in areas with high dengue transmission, while its routine use is not recommended in children under 6 years due to lower vaccine efficacy in this age group [11].

The four serotypes of DENV, i.e., DENV1-4, and ZIKV are all members of the Flaviviridae family. Their RNA genome encodes a polyprotein precursor, which is cleaved by both viral and host proteases. A key role in this process is played by the viral NS2B/NS3 serine protease, which processes the polyprotein into structural components of the virion, as well as non-structural (NS) proteins, essential for viral replication and maturation. Moreover, the NS2B/NS3 protease contributes to immune evasion by cleaving the stimulator of interferon genes (STINGs), thereby suppressing the host immune response [12,13]. These functions make the flaviviral NS2B/NS3 protease a particularly attractive target for the development of antiviral therapies [14].

NS3 is a bifunctional protein composed of 618 amino acid residues. Its *N*-terminal domain (residues 1–168) functions as a serine protease, while the *C*-terminal domain acts as nucleoside triphosphatase (NTPase) and as an RNA helicase. The NS2B cofactor (composed of 130 amino-acid residues) contains a central hydrophilic region of 47 residues that is sufficient to carry out its cofactor activity, flanked by two additional regions. In its active form, the 47-residue core of NS2B is linked to NS3 via a glycine linker, G4SG4, which is also essential for protease activity [15]. The NS3 domain adopts a chymotrypsin-like fold, consisting of two β-barrels (each formed by six β-strands), with the catalytic triad located in the cleft between the two β-barrels. NS2B not only stabilizes the *N*- and *C*-terminal β-barrels but also completes the substrate-binding site with its *C*-terminal region. In fact, it contributes to the correct folding of the protease by inserting a β-strand into the *N*-terminal β-barrels of NS3. The protease can therefore adopt two conformations: an open, inactive form and a closed, active form, in which NS2B fully wraps around the binding site with its *C*-terminal region [16,17].

**Figure 1:**
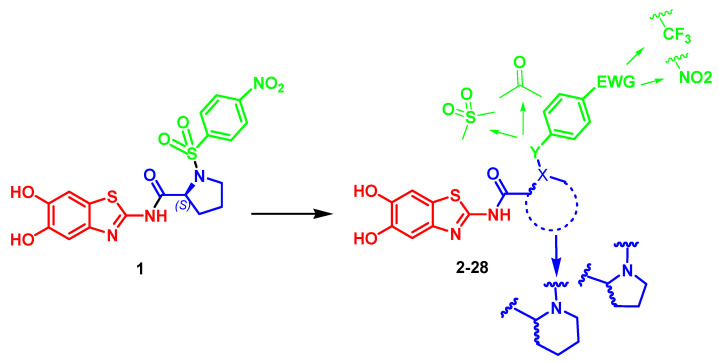
Design of novel inhibitors of DENV and ZIKV NS2B/NS3 serine protease.

To date, both orthosteric inhibitors, which bind to the active site containing the catalytic triad Ser135, His51 and Asp75, fully conserved among flaviviridae proteases, and allosteric inhibitors, which bind to a site close to Asn152, have been reported in the literature. In this respect, the orthosteric inhibitors are featured by two basic moieties (arginine, lysine or a mimetic), which reflect the dibasic substrate recognition motif. The most potent DENV and ZIKV NS2B/NS3 protease inhibitors are peptidic or peptidomimetic, often equipped with electrophilic warheads that covalently target the catalytic serine [14,18,19,20,21,22,23]. Designing and synthesizing orthosteric inhibitors is challenging, not only due to their high molecular weight and positive charge, but also because of the presence of essential basic residues that significantly decrease membrane permeability, thereby limiting cellular uptake [24]. As a result, a convenient design strategy would thus be that of synthesizing allosteric inhibitors, which are uncharged and smaller molecules, targeting a remote binding site that is assumed to lie in proximity of Asn152, a key residue acting as a “molecular switch” from open to closed conformations [25,26]. New proline-based inhibitors have been reported in the literature, endowed with good pharmacological profiles [26], whose binding to the proposed allosteric site was first validated via molecular docking. The allosteric binding site in the proteases was probed using mutagenesis and covalent modification of the obtained cysteine mutants with maleimides, followed by computational elucidation of the possible binding modes [26]. Given the appreciable sequence identity (>50%), the high similarity of the recognized substrates and the close structural similarity between the NS2B/NS3 proteases of DENV and ZIKV, as elucidated from the crystal structures reported in the Protein Data Bank (PDB) (DENV2: PDB 2FOM [27], ZIKV: PDB 5GPI [28]), the design of inhibitors effective against both enzymes represents a viable option. As a result, our investigation was focused on the batch synthesis of allosteric inhibitors of DENV and ZIKV NS2B/NS3. Our study was inspired by the structure of lead compound **1** (Figure 1) that inhibits the serine protease of DENV and ZIKV with IC_50_ values of 8.58 μM and 0.93 μM, respectively [26]. Extensive structure–activity relationships (SAR) were carried out on the critical portions of the molecule; the 5,6-dihydroxybenzo[d]thiazole-2-amine core was kept unchanged because this fragment was demonstrated to be a key structural feature for the interactions with the target proteases, with a key role played by the hydroxyl groups that were able to interact with Asn152 [26].

Concerning the linker connecting the heterocyclic nucleus to the aromatic portion, we introduced a proline nucleus both in the *S* or *R* configuration to evaluate the impact of the stereochemistry on the inhibitory properties. Subsequently, we introduced, in the novel small molecules, a pipecolic acid nucleus, to evaluate the impact of the homologation on the antiviral activity.

Furthermore, in the most interesting compounds, the sulphonyl group has been replaced by a carbonyl linker to investigate its role in the proper accommodation of the inhibitors in the allosteric site, while the nitro (NO_2_) group, present at position 4 of the aromatic ring of the lead compound **1**, was substituted with a trifluoromethyl (CF_3_) group in *ortho*, *meta* or *para* position, to evaluate the impact of this substitution on the inhibition properties, also considering its high electronegativity, strong electron-withdrawing effects and high lipophilicity that can enhance a molecule’s metabolic stability and membrane permeability.

In the present investigation, we report the design and synthesis of a series of novel allosteric inhibitors **2**–**28** (Figure 1), along with the biological studies and docking studies against DENV and ZIKV NS2B/NS3.

## 2. Results and Discussion

### 2.1. Chemistry

**Scheme 1:**
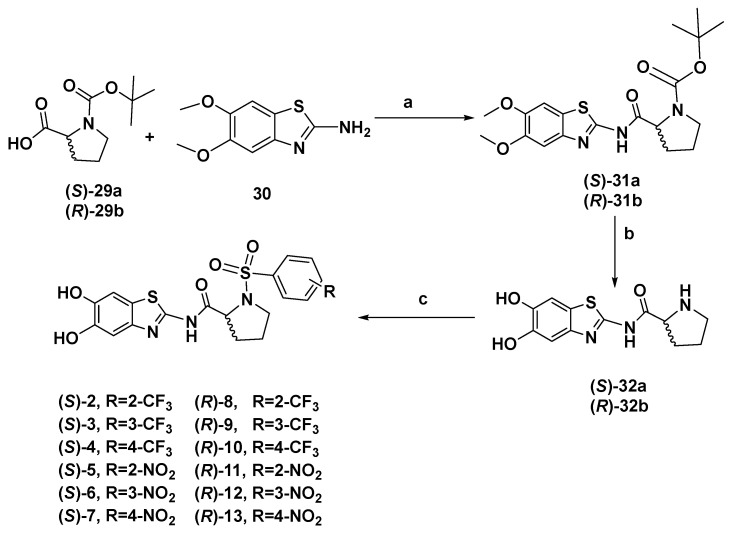
Reagents and conditions: (**a**) TBTU, DIPEA, DMF, 0 °C, r.t. 12 h, (**b**) BBr_3_, DCM, −78 °C, 1 h, then r.t. 12 h, (**c**) R-C_6_H_5_SO_2_Cl, Et_3_N, CH_3_CN, r.t., 12 h.

The synthesis of proline-based compounds **2**–**13** was obtained via an initial coupling reaction between (*tert*-butoxycarbonyl)-L-proline **29a** or (*tert*-butoxycarbonyl)-*R*-proline **29b** and 5,6 dimethoxybenzo[d]thiazol-2-amine **30** to obtain the coupling products **(*S*)–31a** or **(*R*)–31b**, respectively. Boc-deprotection to the NH of the proline nucleus and simultaneous cleavage of the two ether functions was accomplished under drastic reaction conditions, by using BBr_3_ to obtain intermediates **(*S*)–32a** or **(*R*)–32b** bearing the free hydroxyl groups. The final compounds **2**–**13** were then synthesized via a sulfonylation reaction between compounds **(*S*)–32a** or **(*R*)–32b** and the suitable sulfonyl chloride, variously decorated on the aromatic ring with a CF_3_ and NO_2_ group in *ortho*, *meta* and *para* position, to obtain inhibitors **2**–**13** in good yields (Scheme 1).

**Scheme 2:**
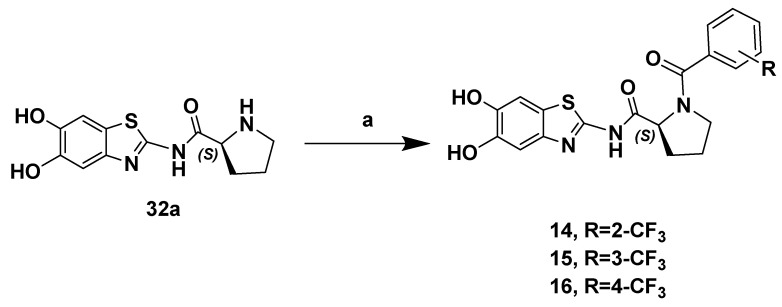
Reagents and conditions: (**a**) R-C_6_H_5_COCl, Et_3_N, CH_3_CN, r.t. 12 h.

The set of molecules **14**–**16**, benzoyl substituted at the NH of the (*S*)-proline, was obtained via a benzoylation reaction between intermediate **(*S*)–32a** and the required benzoyl chlorides bearing a CF_3_ group in the *ortho*, *meta* and *para* position of the aromatic ring (Scheme 2).

**Scheme 3:**
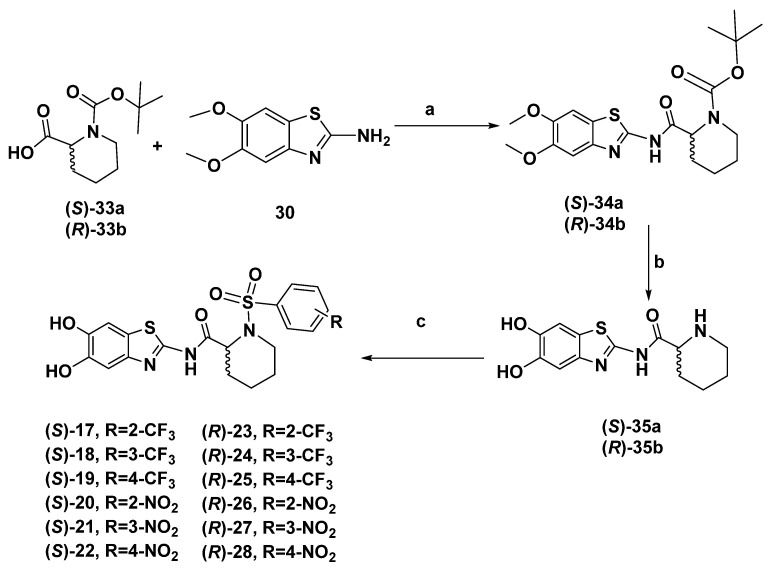
Reagents and conditions: (**a**) TBTU, DIPEA, DMF, 0 °C, r.t. 12 h (**b**) BBr_3_, DCM, −78 °C, 1 h, then r.t. 12 h, (**c**) R-C_6_H_5_SO_2_Cl, Et_3_N, CH_3_CN, r.t. 12 h.

The synthesis of pipecolic-based derivatives **17**–**28** was carried out, applying the same synthetic procedure used for proline-based inhibitors **2**–**13**. Specifically, 5,6-dimethoxybenzo[d]thiazol-2-amine **30** was coupled to (*S*)-1-(*tert*-butoxycarbonyl) piperidine-2-carboxylic acid **(*S*)–33a** or (*R*)-1-(*tert*-butoxycarbonyl) piperidine-2-carboxylic acid **(*R*)–33b** to obtain compounds **(*S*)–34a** or **(*R*)–34b**. After the standard one pot NH deprotection/ether cleavage, accomplished through BBr_3_, intermediates **(*S*)–35a** or **(*R*)–35b** were put to react with the appropriate sulfonyl chlorides yielding the target compounds **17**–**28** in good yields (Scheme 3).

### 2.2. Biological Activity

All inhibitors **2**–**28** were tested in fluorometric assays, in the presence of Bz-Nle-Lys-Arg-Arg-AMC as the fluorogenic substrate (50 µM for DENV, 10 µM for ZIKV), the enzymatic activity was monitored for 15 min and compounds **MB-8** [29] and **MB-53** [30] were used as positive control inhibitors.

From an initial analysis of the obtained biological data, regarding the inhibition of DENV protease and specifically concerning the inhibitors bearing a proline linker, the only active derivatives were those with the *S* configuration. The most active compound was proven to be compound **3**, containing a CF_3_ group in the *meta* position, with an IC_50_ value of 5.0 µM, comparable to that of the lead compound **7**, which was re-synthesized and tested in our experimental conditions (Table 1).

The carbonyl derivatives **8**–**10** showed weak inhibition of the DENV protease, with respect to the corresponding sulfonyl derivatives (e.g., **3** vs. **9**, IC_50_ = 5.0 µM vs. 55.9% of enzyme inhibition), thus highlighting the relevance of the sulfonyl group in properly accommodating the inhibitor within the allosteric site.

Regarding derivatives containing a pipecolic acid residue (compounds **17**–**28**), those active against the DENV protease were those with the *R* configuration, although with IC_50_ values in the mid-micromolar range. In this respect, we performed comparative docking studies on both the *R* and *S* stereoisomers of the *meta* trifluoromethyl pipecolic acid derivatives, **24** and **18**. In agreement with our experimental findings, compound **24** exhibited a more favorable docking score than **18** (−9.6 kcal/mol vs. −7.2 kcal/mol). Importantly, **24** was predicted to establish π–π stacking interactions with Trp83, an interaction that likely contributes significantly to its enhanced binding affinity. Further structural details are provided in Figure S1 of the Supporting Information.

On the contrary, the stereochemistry of the linker does not appear to be crucial for the inhibition of the ZIKV protease, whereas the presence of the pattern of substitution on the aromatic ring, i.e., the presence of a nitro group in *ortho*, *meta* or *para* seems to be fundamental for the inhibition of ZIKV NS2B/NS3 (e.g., **20**–**22** and **26**–**28**).

Concerning the DENV2 Reporter Gene Assay “DENV2proHeLa”, a stable co-transfection of the DENV-2 NS2B-NS3 protease and the *Renilla reniformis* luciferase connected to the oxygen-dependent degradation domain of HIF1α via the NS2A/NS2B protease cleavage site in HeLa cells [31,32], the most active compounds at the cellular level were those containing the pipecolic acid nucleus, thus suggesting the homologation of the proline nucleus could favorably influence the lipophilicity and therefore the cell permeability; in particular, the most active compounds **24** and **27** (EC_50_ = 5.2 and 5.1 µM, respectively) contain both a pipecolic acid core in the *R* configuration.

Interestingly, none of the novel synthesized inhibitors **2**–**28** exhibited a cytotoxic profile, since all inhibitors **2**–**28** showed a CC_50_ > 100 µM, and they were also found to be selective inhibitors for the viral protease since they did not inhibit the bovine serine proteases thrombin and trypsin at 25 µM and 50 µM, respectively.

**Figure 2:**
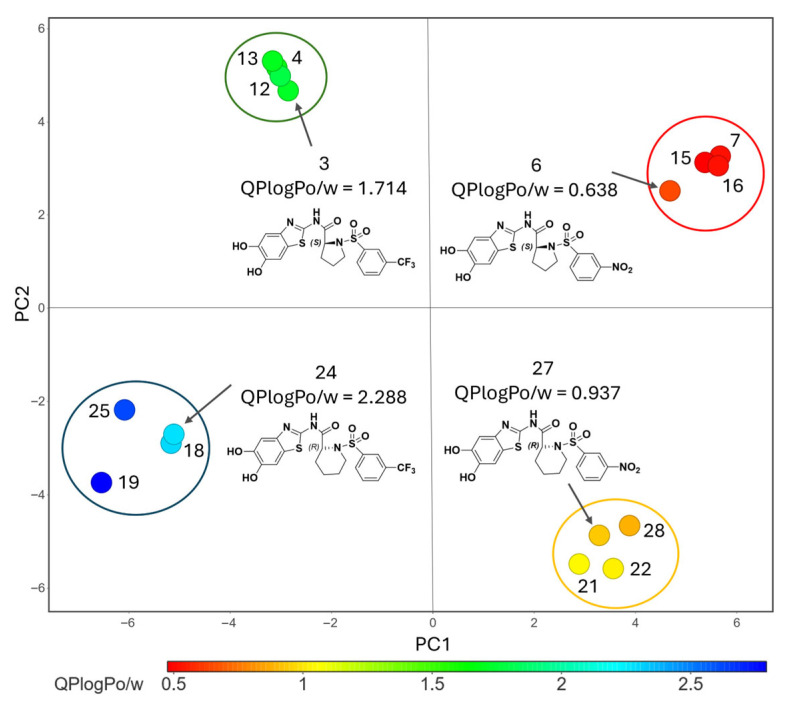
PCA carried out on pipecolic and proline sulfophenyl derivatives (i.e., **3**, **4**, **6**, **7**, **12**, **13**, **15**, **16**, **18**, **19**, **21**, **22**, **24**, **25**, **27**, **28**). PC1 and PC2 account for 49.1% and 41.2% of the explained variance, respectively. The color scale ranges from red to blue, indicating increasing predicted lipophilicity.

For the sake of completeness, principal component analysis (PCA) was performed on pipecolic and proline sulfonyl derivatives substituted at the *meta* and *ortho* positions with either trifluoromethyl or nitro groups (i.e., **3**, **4**, **6**, **7**, **12**, **13**, **15**, **16**, **18**, **19**, **21**, **22**, **24**, **25**, **27**, **28**), considering that the most active inhibitors against both the protease and at cellular levels are those *meta*-substituted on the aromatic ring. As shown in Figure 2, this analysis clearly discriminated clusters corresponding to pipecolic versus proline derivatives, as well as clusters defined by trifluoromethyl versus nitro substitution. The colormap further indicates that pipecolic acid derivatives are predicted to display higher lipophilicity relative to their proline counterparts. A similar trend is observed when comparing trifluoromethyl- with nitro-substituted derivatives.

### 2.3. Docking Studies and In Silico Investigation

**Figure 3:**
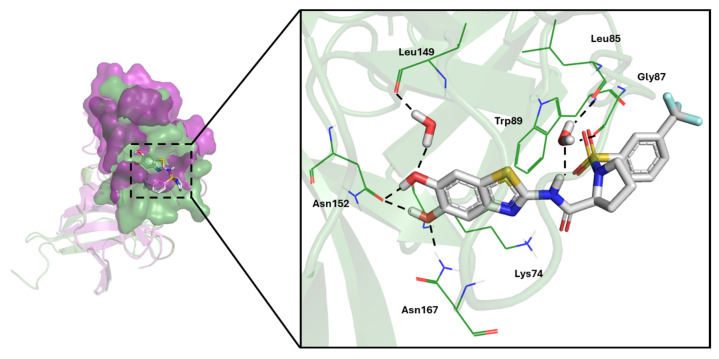
On the left-hand side, overlay of the allosteric pocket of NS2B/NS3 proteases of dengue (PDB: 2FOM, green cartoon) and Zika (PDB ID: 5GPI, purple cartoon) viruses mapped by using BioGPS software. On the right-hand side, zoomed in view of the IFD pose of **3** in white sticks, within the allosteric cavity of dengue virus (PDB ID: 2FOM). Water molecules were rendered as sticks, whereas black dashed lines indicated HBs. For the sake of completeness, all molecular interactions observed in IFD simulations were automatically flagged using GLIDE software. Schrödinger Release 2024-1.

Assessing the druggability of allosteric sites is extremely important to address the rational design of new bioactive compounds [33,34]. To this end, we carried out the in silico mapping of X-ray-solved NS2B/NS3 proteases of DENV and ZIKV. By using BioGPS software v2.1.364 one allosteric pocket was spotted for NS2B/NS3 proteases of ZIKV and DENV. This cavity was in the proximity of Asn152, a conserved residue shared by NS2B/NS3 proteases of ZIKV and DENV and played as “molecular switch” in the transition from the closed to open protein conformation [35]. As depicted on the left-hand side of Figure 3, the identified allosteric cavities showed a similar shape and could provide a solid rationale for further analyses. All the structural details concerning with these allosteric pockets are reported in Table 2.

**Figure 4:**
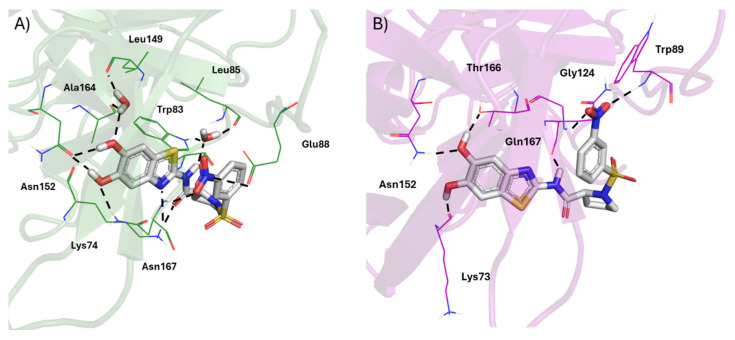
The panels (**A**) and (**B**) show the IFD poses of **6** in white sticks towards the allosteric site of NS2B/NS3 proteases of DENV (PDB: 2FOM, green cartoon) and ZIKV (PDB ID: 5GPI, purple cartoon) viruses, respectively. Black dashed lines indicate HBs.

Additional computational investigations were thus carried out to shed light on the molecular rationale behind the observed binding and selectivity of **2**, **3**, **4**, **5**, **6** and **7** towards the identified allosteric cavity of NS2B/NS3 proteases. Interestingly, **3** was found to be the most active towards the NS2B/NS3 protease of DENV, with an IC_50_ value of 5.0 ± 0.29 µM. The IFD simulation returned a convenient pose, experiencing the main expected interactions at the allosteric pocket of the NS2B/NS3 protease of DENV and a value of the docking score comparable to the other derivatives (see Table S1 of the Supporting Information). As shown in Figure 3, the two hydroxyl groups of the benzothiazole ring formed HBs with the side chains of key residue Asn152 (1.70 Å) and Asn167 (1.90 Å). Moreover, the two water molecules were supposed to be crucial for the effective inhibition of the NS2B/NS3 protease of dengue by forming a stable HB network, as previously reported [26]. The first water molecule (i.e., HOH247) bridged the amide group of **3** with the backbone of Leu85 and Gly87. The second water molecule (i.e., HOH230) interfaced the hydroxyl group of **3** with Leu149. Similar poses were obtained for **2** and **4** (cf. Figure S2). From an experimental point of view, **2**, **3** and **4** showed a high selectivity for DENV due to a drop in inhibition towards the NS2B/NS3 protease of ZIKV. This is likely due to the loss of the key interactions established by Asn152 and a water molecule (i.e., HOH247) (see Figure S3 of the Supporting Information). As far as nitro-substituted benzothiazole derivatives **5**, **6** and **7** are concerned, a less pronounced selectivity was observed. The IFD poses of **6** within the allosteric site of NS2B/NS3 proteases of DENV and ZIKV showed comparable posing and scoring, as depicted in the Figure 4 and in the Table S1 of the Supporting Information.

Remarkably, the hydroxyl groups of the benzothiazole ring of **6** returned HB interactions with the side chain of Asn152 of the NS2B/NS3 protease of DENV (Figure 4A). A first water molecule (HOH230) linked, via HB, the hydroxyl group of **6** and Leu149. Furthermore, a second water molecule (i.e., HOH247) bridged the amide group of **6** with the backbone of Leu85 and Trp83. Additional significant HB contacts were established between the amide of **6** and Asn167, as well as between its m-nitro group and residues Lys74 and Glu88. As shown in panel B of Figure 4, the critical engagement with key residue Asn152 was reinforced by an additional interaction established by the nitro group with Trp89 in the case of the NS2B/NS3 protease of ZIKV. Taken together, this helped to explain, at a molecular level of detail, the lack of selectivity of **6**. Similar behavior was also observed for **7** when binding the allosteric pocket of NS2B/NS3 proteases of ZIKV and DENV, as shown in Figure S4 of the Supporting Information.

## 3. Materials and Methods

### 3.1. Chemistry

All reagents and solvents were obtained from commercial suppliers and were used without any further purification. To determine the purity of compounds, elemental analyses were carried out on a C. Erba Model 1106 (Elemental Analyser for C, H, and N, Cornaredo (MI, Italy) instrument, and the obtained results were within ± 0.4% of the theoretical values. Merck silica gel 60 F254 plates were used for analytical TLC; flash column chromatography was performed on a Merck silica gel (200–400 mesh). ^1^H and ^13^C and NMR spectra were recorded on a Varian 500 MHz spectrometer (Palo Alto, CA, USA) equipped with a ONE_NMR probe and operating at 499.74 and 125.73 MHz for ^1^H and ^13^C, respectively. We used the residual signal of the deuterated solvent as an internal standard. Splitting patterns are described as singlet (s), doublet (d), doublet of doublet (dd), triplet (t), quartet (q), multiplet (m) or broad singlet (bs). ^1^H and ^13^C NMR chemical shifts (δ) are expressed in ppm, and coupling constants (*J*) are given in Hz.

#### 3.1.1. *Tert*-butyl (*S*)-2-((5,6-Dimethoxybenzo[d]thiazol-2-yl) Carbamoyl)pyrrolidine-1-carboxylate (**31a**)

(*Tert*-butoxycarbonyl)-L-proline **29a** (2.05 g, 9.51 mmol) was dissolved in DMF (15 mL); then, cooling the solution with an ice water, DIPEA (1.81 g, 1.83 mL, 14 mmol) and O-(benzotriazole-1-yl)-*N*,*N*,*N*′,*N*′-tetramethyluroniumtetrafluoroborate (TBTU) (4.50 g, 14 mmol) were added, and the mixture was stirred for 10 min. After this time, 5,6-dimethoxybenzo[d]thiazol-2-amine **30** (2 g, 9.51 mmol) was added, the ice bath was removed and the reaction mixture was stirred at room temperature for 12 h. Water (50 mL) was added, and the mixture was extracted with ethyl acetate three times (50 mL × 3). The organic layer was separated and washed two times with a saturated NaCl solution (brine). The organic layer was dried over Na_2_SO_4_, and the solvent was removed using reduced pressure. The crude was purified via column chromatography by using as eluent mixture ethyl acetate/light petroleum 8:2 to yield the pure product **31a** as a pale-yellow solid (2.8 g, 72.3%). R_f_ = 0.40 (ethyl acetate/light petroleum 8:2. ^1^H NMR (500 MHz, CDCl_3_): 1.39 (s, 9H), 1.79–1.91 (m, 2H), 1.90–1.98 (m, 2H), 3.28–3.71 (m, 2H), 3.88 (s, 6H), 4.03–4.10 (m, 1H), 7.18 (s, 1H), 7.33 (m, 1H) ppm; ^13^C NMR (125 MHz, CDCl3): 21.00, 28.83, 30.98, 47.28, 56.11, 56.34, 60.36, 81.01, 102.72, 103.25, 124.11, 142.10, 147.32, 150.15, 154.78, 161.96, 171.12 ppm.

#### 3.1.2. (*S*)-*N*-(5,6-Dihydroxybenzo[d]thiazol-2-yl)pyrrolidine-2-carboxamide (**32a**)

*Tert*-butyl-(S)-2-(2-(5,6-dimethoxybenzo[b]thiophen-2-yl)acetyl)pyrrolidine-1carboxylate **31a** (2.0 g, 4.9 mmol) was dissolved in dichloromethane (15 mL); then, the solution was brought to a temperature of −78 °C using a dry ice bath dissolved in acetone. In these drastic conditions, BBr_3_ (24.5 mmol, 24.5 mL of 1M solution in dichloromethane) was added dropwise, and after 30 min, the ice bath was removed, and the solution was stirred for 12 h at room temperature. After this time, methanol (10 mL) was added to the reaction solution cooling the mixture with an ice bath and putting it under stirring for 30 min. The solvent was then evaporated in vacuo to obtain the pure product **32a** that had a solid yellow consistency. (1 g, 73%). ^1^H NMR (500 MHz, DMSO): 1.88–1.95 (m, 2H), 1.96–2.03 (m, 1H), 2.33–2.43 (m, 1H), 3.21–3.32 (m, 2H), 4.43–4.51 (m, 1H), 7.10 (s, 1H), 7.25 (s, 1H) ppm; ^13^C NMR (125 MHz, DMSO): 23.54, 29.30, 45.96, 60.52, 106.53, 108.02, 127.73, 141.26, 144.30, 145.85, 161.66, 173.58 ppm.

#### 3.1.3. (*S*)-*N*-(5,6-Dihydroxybenzo[d]thiazol-2-yl)-1-((2-trifluoromethyl)phenyl)sulfonyl)pyrrolidine-2-carboxamide (**2**)

(*S*)-*N*-(5,6-dihydroxybenzo[d]thiazol-2-yl)pyrrolidine-2-carboxamide **32a** (167 mg, 0.6 mmol) was dissolved in acetonitrile (15 mL), and triethylamine (121 mg, 1.2 mmol) was added to the solution. Then, 2-(trifluoromethyl) benzenesulfonyl chloride (171 mg, 0.7 mmol) was added to the solution, and the reaction mixture was stirred at room temperature for 12 h. Water (10 mL) was added, and the mixture was extracted with dichloromethane three times (50 mL × 3). The organic layer was separated and washed two times with saturated NaCl solution (brine). The solvent of extraction was then dried with Na_2_SO_4_ and the solvent was removed using reduced pressure. The crude was purified via column chromatography by using as eluent mixture ethyl acetate/light petroleum 5:5 to yield the pure product **2** as a yellow powder (138 mg, 46.3%). R_f_ = 0.36 (ethyl acetate/light petroleum 5:5). ^1^H NMR (500 MHz, CDCl_3_): 1.84–1.92 (m, 1H), 1.95–2.03 (m, 1H), 2.05–2.18 (m, 1H), 2.18–2.25 (m, 1H), 3.33–3.40 (m, 1H), 3.52–3.61 (m, 1H), 4.61 (dd, *J* = 8.6 Hz and 3.3 Hz, 1H), 7.34 (s, 1H), 7.54 (s, 1H), 7.87–7-74 (m, 1H), 7.79 (t, *J* = 6.4 Hz, 1H), 7.91 (t, *J* = 6.4 Hz, 1H), 8.10 (d, *J* = 7.6 Hz, 1H) ppm; ^13^C NMR (125 MHz, CDCl_3_): 25.25, 29.09, 49.47, 60.45, 116.04, 116.86, 121.26 (q, *J* = 275.8 Hz), 128.06 (q, *J* = 33.4 Hz), 131.31, 131.69, 132.61 (d, *J* = 3.8 Hz),132.79, 133.74 (d, *J* = 10.5 Hz), 134.73, 137.33, 139.87, 147.39, 160.60, 170.55 ppm. Elemental analysis: calcd for C_19_H_16_F_3_N_3_O_5_S_2_: C, 46.82; H, 3.31; N, 8.62; found: C, 47.18 H, 3.06 N, 8.93.

#### 3.1.4. (*S*)-*N*-(5,6-Dihydroxybenzo[d]thiazol-2-yl)-1-((3-trifluoromethyl)phenyl)sulfonyl)pyrrolidine-2-carboxamide (**3**)

With a similar procedure described for compound **2**, (*S*)-*N*-(5,6-dihydroxybenzo[d]thiazol-2-yl)pyrrolidine-2-carboxamide **32a** (167 mg, 0.6 mmol) and 3-(trifluoromethyl) benzenesulfonyl chloride (171 mg, 0.7 mmol), triethylamine (121 mg, 1.2 mmol) in the same reaction conditions gave compound **3**. Then, the crude was purified via column chromatography by using, as an eluent mixture, ethyl acetate/light petroleum 4:6 to yield the pure product **3** as a yellow powder (134.3 mg, 46.3%). R_f_ = 0.75 (ethyl acetate/light petroleum 4:6). ^1^H NMR (500 MHz, CDCl_3_): 1.74–1.83 (m, 1H), 1.84–1.99 (m, 2H), 2.25–2.35 (m, 1H), 3.22–3.34 (m, 1H), 3.62–3.72 (m, 1H), 4.42 (dd, *J* = 8.4 Hz and 2.8 Hz, 1H), 7.54 (s, 1H), 7.66 (t, *J* = 7.9 Hz, 2H), 7.71 (s, 1H), 8.09 (d, *J* = 5.5 Hz, 1H), 8.13 (s, 1H) ppm; ^13^C NMR (125 MHz, CDCl_3_): 25.30, 29.50, 49.98, 62.29, 116.37, 117.05, 123.01 (q, *J* = 273.0 Hz ), 126.17, 130.29, 130.56, 131.16, 131.44, 132.19 (q, *J* = 34.2 Hz), 136.29, 137.25, 139.74, 147.79, 160.12, 170.18 ppm. Elemental analysis: calcd for C_19_H_16_F_3_N_3_O_5_S_2_: C, 46.82; H, 3.31; N, 8.62; found: C, 46.57 H, 3.67 N, 8.48.

#### 3.1.5. (*S*)-*N*-(5,6-Dihydroxybenzo[d]thiazol-2-yl)-1-((4-trifluoromethyl)phenyl)sulfonyl)pyrrolidine-2-carboxamide (**4**)

With a similar procedure previously described for compound **2**, (*S*)-*N*-(5,6-dihydroxybenzo[d]thiazol-2-yl)pyrrolidine-2-carboxamide **32a** (167 mg, 0.6 mmol), 4-(trifluoromethyl) benzenesulfonyl chloride (171 mg, 0.7 mmol) and triethylamine (121 mg, 1.2 mmol) were used to obtain compound **4**. Then, the crude was purified via column chromatography by using, as an eluent mixture, ethyl acetate/light petroleum 4:6 to yield the pure product **4** as a yellow powder (151.5 mg, 55.2%). R_f_ = 0.87 (ethyl acetate/light petroleum 4:6). ^1^H NMR (500 MHz, CDCl_3_): 1.75–1.82 (m, 1H), 1.82–1.89 (m, 1H), 1.89–1.97 (m, 1H), 2.27–2.38 (m, 1H), 3.22–3.33 (m, 1H), 3.63–3.74 (m, 1H), 4.36 (dd, *J* = 8.5 Hz and 3.2 Hz, 1H), 7.56 (s, 1H), 7.75 (s, 1H), 7.86 (d, *J* = 8.1 Hz, 2H), 8.04 (d, *J* = 8.1 Hz, 2H) ppm; ^13^C NMR (125 MHz, CDCl_3_): 24.76, 30.27, 50.17, 62.38, 116.25, 117.12, 123.42 (q, *J* = 272.5 Hz), 128.60, 129.20, 129.28, 137.42, 138.84, 139.19, 139.92, 147.67, 159.99, 169.97 ppm. Elemental analysis: calcd for C_19_H_16_F_3_N_3_O_5_S_2_: C, 46.82; H, 3.31; N, 8.62; found: C, 46.92 H, 2.93 N, 8.83.

#### 3.1.6. (*S*)-*N*-(5,6-Dihydroxybenzo[d]thiazol-2-yl)-1-((2-nitrophenyl)sulfonyl)pyrrolidine-2-carboxamide (**5**)

With a similar procedure previously described for compound **2**, (*S*)-*N*-(5,6-dihydroxybenzo[d]thiazol-2-yl)pyrrolidine-2-carboxamide **32a** (167 mg, 0.6 mmol) was reacted with 2-nitrobenzenesulfonyl chloride (155 mg, 0.7 mmol) and triethylamine (121 mg, 1.2 mmol) to obtain compound **5**. The crude was purified via column chromatography by using, as an eluent mixture, ethyl acetate/light petroleum 5:5 to yield the pure product **5** as a yellow powder (138.4 mg, 47.3%). R_f_ = 0.10 (ethyl acetate/light petroleum 5:5). ^1^H NMR (500 MHz, CDCl_3_) 1.97–2.04 (m, 2H), 2.19–2.27 (m, 1H), 2.33–2.45 (m, 1H), 3.57–3.66 (m, 1H), 3.69–3.80 (m, 1H), 4.73 (dd, *J* = 9.0 Hz and 3.2 Hz, 1H), 7.53 (s, 1H), 7.69–7.72 (m, 2H), 7.73 (s, 1H), 7.84 (d, *J* = 8.5 Hz, 1H), 8.00 (d, *J* = 8.0 Hz, 1H) ppm; ^13^C NMR (125 MHz, CDCl_3_): 24.83, 31.01, 49.88, 62.14, 116.36, 117.26, 124.70, 128.43, 130.41, 131.49, 132.30, 134.89, 137.50, 140.01, 147.67, 148.39, 159.80, 170.13 ppm. Elemental analysis: calcd for C_18_H_16_N_4_O_7_S_2_: C, 46.55; H, 3.47; N, 12.06; found: C, 46.40 H, 3.68 N, 11.74.

#### 3.1.7. (*S*)-*N*-(5,6-Dihydroxybenzo[d]thiazol-2-yl)-1-((3-nitrophenyl)sulfonyl)pyrrolidine-2-carboxamide (**6**)

With a similar procedure previously described for compound **2**, (*S*)-*N*-(5,6-dihydroxybenzo[d]thiazol-2-yl)pyrrolidine-2-carboxamide **32a** (167 mg, 0.6 mmol) was reacted with 3-nitrobenzenesulfonyl chloride (155 mg, 0.7 mmol) and triethylamine (121 mg, 1.2 mmol) to obtain compound **6**. The crude was purified via column chromatography by using, as an eluent mixture, ethyl acetate/light petroleum 5:5 to yield the pure product **6** as a yellow powder (116.6 mg, 35.5%). R_f_ = 0.31 (ethyl acetate/light petroleum 5:5). ^1^H NMR (500 MHz, CDCl_3_): 1.79–1.88 (m, 1H), 1.90–2.00 (m, 2H), 2.26–2.32 (m, 1H), 3.29–3.37 (m, 1H), 3.67–3.75 (m, 1H), 4.43 (dd, *J* = 8.3 Hz and 3.5 Hz, 1H), 7.50 (s, 1H), 7.73 (s, 1H), 7.80 (d, *J* = 8.1 Hz, 1H), 7.83 (d, *J* = 8.1 Hz, 1H), 8.57 (d, *J* = 9.4 Hz, 1H), 8.68 (s, 1H) ppm; ^13^C NMR (125 MHz, CDCl_3_): 24.77, 30.47, 50.10, 62.38, 116.36, 117.27, 122.96, 129.40, 131.14, 132.04, 133.39, 136.97, 138.08, 139.46, 147.80, 148.34, 160.19, 169.94 ppm. Elemental analysis: calcd for C_18_H_16_N_4_O_7_S_2_: C, 46.55; H, 3.47; N, 12.06; found: C, 46.86 H, 3.31 N, 12.24.

#### 3.1.8. (*S*)-*N*-(5,6-Dihydroxybenzo[d]thiazol-2-yl)-1-((4-nitrophenyl)sulfonyl)pyrrolidine-2-carboxamide (**7**)

With a similar procedure previously described for compound **2**, (*S*)-*N*-(5,6-dihydroxybenzo[d]thiazol-2-yl)pyrrolidine-2-carboxamide **32a** (111 mg, 0.4 mmol) was reacted with 4-nitrobenzenesulfonyl chloride (111 mg, 0.5 mmol) and triethylamine (81 mg, 0.8 mmol) to obtain compound **7**. Then, the crude was purified via column chromatography by using, as an eluent mixture, ethyl acetate/light petroleum 5:5 to yield the pure product **7** as a yellow powder (49 mg, 45%). R_f_ = 0.78 (ethyl acetate/light petroleum 5:5). ^1^H NMR (500 MHz, CDCl_3_): 1.85–1.99 (m, 2H), 2.01–2.11 (m, 1H), 2.19–2.26 (m, 1H), 3.25–3.33 (m, 1H), 3.50–3.59 (m, 1H), 4.43 (dd, *J* = 8.5 Hz and 4.2 Hz, 1H), 7.52 (s, 1H), 7.68 (s, 1H), 8.03 (d, *J* = 7.0 Hz, 2H), 8.11 (d, *J* = 7.0 Hz, 2H) ppm; ^13^C NMR (125 MHz, CDCl_3_): 24.43, 28.57, 49.15, 60.92, 115.78, 118.13, 124.70, 124.98, 128.84, 139.19, 142.31, 147.76, 150.08, 151.17, 161.32, 171.91 ppm. Elemental analysis: calcd for C_18_H_16_N_4_O_7_S_2_: C, 46.55; H, 3.47; N, 12.06; found: C, 46.38 H, 3.69 N, 11.76.

#### 3.1.9. *Tert*-butyl (*R*)-2-((5,6-Dimethoxybenzo[d]thiazol-2-yl)carbamoyl)pyrrolidine-1-carboxylate (**31b**)

With a similar procedure previously described for compound **31a** (*tert*-butoxy carbonyl)-*R*-proline **29b** (2.04 g, 9.51 mmol), DIPEA (1.83 g, 1.85 mL, 14.2 mmol), O-(benzotriazole-1-yl)-*N*,*N*,*N*′,*N*′-tetramethyluroniumtetrafluoroborate (TBTU) (4.56 g, 14.2 mmol) and 5,6-dimethoxybenzo[d]thiazol-2-amine **30** (2 g, 9.51 mmol) were reacted to give the final product **31b**. The crude was purified via column chromatography by using, as an eluent mixture, ethyl acetate/light petroleum 8:2 to yield the pure product **31b** as a pale-yellow solid (3.01 g, 77.8%). R_f_ = 0.61 (ethyl acetate/light petroleum 8:2. ^1^H NMR (500 MHz, DMSO): 1.42 (s, 9H), 1.82–2.06 (m, 2H), 2.13–2.37 (m, 2H), 3.39–3.58 (m, 2H), 3.79 (s, 6H), 4.44–4.68 (m, 1H), 7.27 (s, 1H), 7.52 (s, 1H) ppm; ^13^C NMR (125 MHz, DMSO): 24.71, 28.92, 30.43, 46.68, 55.75, 55.96, 60.18, 79.07, 103.59, 103.72, 127.21, 143.78, 146.99, 148.92, 153.64, 161.50, 169.90 ppm.

#### 3.1.10. (*R*)-*N*-(5,6-Dihydroxybenzo[d]thiazol-2-yl)pyrrolidine-2-carboxamide (**32b**)

According to the procedure described for compound **32a** in a round-bottom flask, *tert*-butyl (*R*)-2-((5,6-dimethoxybenzo[d]thiazol-2-yl) carbamoyl) pyrrolidine-1-carboxylate **31b** (978 mg, 2.4 mmol) and BBr_3_ (12 mmol, 12 mL of a 1 M solution in dichloromethane) were reacted to give compound **32b** as a white powder (810 mg, 63.3%), which was used for the next step without purification. ^1^H NMR (500 MHz, DMSO): 1.91–1.98 (m, 2H), 1.98–2.05 (m, 1H), 2.36–2.46 (m, 1H), 3.23–3.35 (m, 2H), 4.46–4.54 (m, 1H), 7.13 (s, 1H), 7.27 (s, 1H) ppm; ^13^C NMR (125 MHz, DMSO): 23.37, 29.13, 45.79, 60.35, 106.36, 107.85, 127.56, 141.09, 144.13, 145.68, 161.49, 173.41 ppm.

#### 3.1.11. (*R*)-*N*-(5,6-Dihydroxybenzo[d]thiazol-2-yl)-1-((2-trifluoromethyl)phenyl)sulfonyl)pyrrolidine-2-carboxamide (**8**)

With a similar procedure previously described for compound **2**, (*R*)-*N*-(5,6-dihydroxybenzo[d]thiazol-2-yl)pyrrolidine-2-carboxamide **32b** (28 mg, 0.1 mmol), 2-(trifluoromethyl)benzenesulfonyl chloride (27 mg, 0.11 mmol) and triethylamine (20 mg, 0.2 mmol) were reacted to give product **8**. The crude was purified via column chromatography by using, as an eluent mixture, ethyl acetate/light petroleum 6:4 to yield the pure product **8** as a yellow powder (16 mg, 41%), R_f_ = 0.33 (ethyl acetate/light petroleum 6:4). ^1^H NMR (500 MHz, CDCl_3_): 1.87–2.00 (m, 2H), 2.16–2.30 (m, 2H), 3.54–3.60 (m, 2H), 4.70 (dd, *J* = 8.4 Hz and 3.6 Hz, 1H), 7.43 (s, 1H), 7.62 (s, 1H), 7.69 (t, *J* = 6.4 Hz, 2H), 7.84 (d, *J* = 7.6 Hz, 2H) ppm; ^13^C NMR (125 MHz, CDCl_3_): 25.00, 29.35, 49.73, 62.20, 116.29, 117.12, 121.52 (q, *J* = 274.5 Hz), 127.50, 128.31 (q, *J* = 33.4 Hz), 128.99, 129.35, 131.56, 131.95, 134.98, 140.12, 146.43, 147.65, 160.86, 170.81, 174.05 ppm. Elemental analysis: calcd for C_20_H_16_F_3_N_3_O_4_S: C, 53.21; H, 3.57; N, 9.31; found: C, 53.31 H, 3.21 N, 9.52.

#### 3.1.12. (*R*)-*N*-(5,6-Dihydroxybenzo[d]thiazol-2-yl)-1-((3-trifluoromethyl) phenyl)sulfonyl)pyrrolidine-2-carboxamide (**9**)

With a similar procedure previously described for compound **2**, (*R*)-*N*-(5,6-dihydroxybenzo[d]thiazol-2-yl)pyrrolidine-2-carboxamide **32b** (67 mg, 0.24 mmol), 3-(trifluoromethyl) benzene sulfonyl chloride (64 mg, 0.26 mmol) and triethylamine (48 mg, 0.48 mmol) were reacted to give product **9**. The crude was purified via column chromatography by using, as an eluent mixture, ethyl acetate/light petroleum 3:7 to yield the pure product **9** as a yellow powder (11 mg, 15%), R_f_ = 0.35 (ethyl acetate/light petroleum 3:7). ^1^H NMR (500 MHz, CDCl_3_): 1.84–1.96 (m, 1H), 2.02–2.16 (m, 1H), 2.21–2.33 (m, 2H), 3.50–3.59 (m, 1H), 3.67–3.76 (m, 1H), 4.99 (m, 1H), 7.44 (s, 1H), 7.62 (s, 1H), 7.68 (t, *J* = 8.5 Hz, 1H), 7.81 (d, *J* = 9.2 Hz, 2H), 8.16 (s, 1H) ppm; ^13^C NMR (125 MHz, CDCl_3_): 25.30, 29.19, 50.70, 60.90, 115.46, 115.65, 124.52 (q, *J* = 272.5 Hz), 126.78, 126.85, 127.36, 129.08, 130.08, 131.10 (q, *J* = 33.6 Hz), 136.35, 138.87, 141.16, 146.60, 163.04, 170.83, 173.58 ppm. Elemental analysis: calcd for C_20_H_16_F_3_N_3_O_4_S: C, 53.21; H, 3.57; N, 9.31; found: C, 52.94 H, 3.68 N, 8.97.

#### 3.1.13. (*R*)-*N*-(5,6-Dihydroxybenzo[d]thiazol-2-yl)-1-((4-trifluoromethyl)phenyl)sulfonyl)pyrrolidine-2-carboxamide (**10**)

With a similar procedure previously described for compound **2**, (*R*)-N-(5,6-dihydroxybenzo[d]thiazol-2-yl)pyrrolidine-2-carboxamide **32b** (56 mg, 0.2 mmol), 4-(trifluoromethyl) benzenesulfonyl chloride (54 mg, 0.22 mmol) and triethylamine (40 mg, 0.4 mmol) were reacted to give product **10**. The crude was purified via column chromatography by using, as an eluent mixture, ethyl acetate/light petroleum 5:5 to yield the pure product **10** as a yellow powder (10 mg, 20.5%). R_f_ = 0.45 (ethyl acetate/light petroleum 5:5). ^1^H NMR (500 MHz, CDCl_3_): 1.89–2.00 (m, 1H), 2.15–2.25 (m, 1H), 2.24–2.35 (m, 2H), 3.54–3.62 (m, 1H), 3.65–3.75 (m, 1H), 5.07 (dd, *J* = 7.7 Hz and 5.3 Hz, 1H), 7.66 (d, *J* = 7.8 Hz, 2H), 7.73 (d, *J* = 7.8 Hz, 2H), 7.78 (s, 1H), 7.88 (s, 1H) ppm; ^13^C NMR (125 MHz, CDCl_3_): 25.35, 28.77, 50.70, 60.66, 115.73, 115.79, 125.65 (q, *J* = 274.2 Hz), 127.99, 130.49, 130.57, 131.89 (d, *J* = 12.7 Hz), 135.52, 138.92, 141.23, 147.08, 163.25, 170.64, 173.54 ppm. Elemental analysis: calcd for C_20_H_16_F_3_N_3_O_4_S: C, 53.21; H, 3.57; N, 9.31; found: C, 53.44 H, 3.24 N, 9.43.

#### 3.1.14. (*R*)-*N*-(5,6-Dihydroxybenzo[d]thiazol-2-yl)-1-((2-nitrophenyl)sulfonyl)pyrrolidine-2-carboxamide (**11**)

With a similar procedure previously described for compound **2**, (*R*)-N-(5,6-dihydroxybenzo[d]thiazol-2-yl)pyrrolidine-2-carboxamide **32b** (167 mg, 0.6 mmol), 2-nitrobenzenesulfonyl chloride (155 mg, 0.7 mmol) and triethylamine (121 mg, 1.2 mmol) were reacted to give compound **11**. The crude was purified via column chromatography by using, as an eluent mixture, ethyl acetate/light petroleum 4:6 to yield the pure product **11** as a yellow powder (20 mg, 21.5%), R_f_ = 0.35 (ethyl acetate/light petroleum 4:6). ^1^H NMR (500 MHz, CDCl_3_): 1.90–1.98 (m, 1H), 2.01–2.09 (m, 1H), 2.11–2.23 (m, 1H), 2.23–2.31 (m, 1H), 3.39–3.46 (m, 1H), 3.57–3.66 (m, 1H), 4.67 (dd, J = 8.6 Hz and 3.3 Hz, 1H), 7.40 (s, 1H), 7.60 (s, 1H), 7.77 (d, *J* = 7.8 Hz, 1H), 7.84 (t, *J* = 6.4 Hz, 1H), 7.97 (t, *J* = 6.4 Hz, 1H), 8.15 (d, *J* = 7.6 Hz, 1H) ppm; ^13^C NMR (125 MHz, CDCl_3_): 24.49, 30.65, 49.22, 61.69, 115.78, 116.61, 121.01 (q, *J* = 275.8 Hz), 127.80 (q, *J* = 33.4 Hz), 128.48, 128.84, 131.05, 131.44, 133.14, 137.08, 139.61, 146.46, 147.14, 160.35, 170.30 ppm. Elemental analysis: calcd for C_19_H_16_F_3_N_3_O_5_S_2_: C, 46.82; H, 3.31; N, 8.62; found: C, 46.46 H, 3.55 N, 8.32.

#### 3.1.15. (*R*)-*N*-(5,6-Dihydroxybenzo[d]thiazol-2-yl)-1-((3-nitrophenyl)sulfonyl)pyrrolidine-2-carboxamide (**12**)

With a similar procedure previously described for compound **2**, (*R*)-*N*-(5,6-dihydroxybenzo[d]thiazol-2-yl)pyrrolidine-2-carboxamide **32b** (67 mg, 0.24 mmol), 3-nitrobenzenesulfonyl chloride (58 mg, 0.26 mmol) and triethylamine (48 mg, 0.48 mmol) were reacted to obtain compound **12**. The crude was purified via column chromatography by using, as an eluent mixture, ethyl acetate/light petroleum 4:6 to yield the pure product **12** as a white powder (26 mg, 28%), R_f_ = 0.33 (ethyl acetate/light petroleum 4:6). ^1^H NMR (500 MHz, CDCl_3_): 1.90–2.03 (m, 1H), 2.09–2.23 (m, 1H), 2.27–2.39 (m, 2H), 3.56–3.66 (m, 1H), 3.73–3.83 (m, 1H), 5.05 (m, 1H), 7.43–7.55 (m, 3H), 7.68 (s, 1H), 7.82 (s, 1H), 8.22 (s, 1H) ppm; ^13^C NMR (125 MHz, CDCl_3_): 25.05, 28.93, 50.44, 60.64, 115.21, 115.40, 124.27 (q, *J* = 272.5 Hz), 126.60, 127.10, 128.83, 129.15, 130.74, 133.04, 138.62, 140.90, 145.43, 146.35, 162.79, 170.58 ppm. Elemental analysis: calcd for C_19_H_16_F_3_N_3_O_5_S_2_: C, 46.82; H, 3.31; N, 8.62; found: C, 46.68 H, 3.49 N, 8.35.

#### 3.1.16. (*R*)-*N*-(5,6-Dihydroxybenzo[d]thiazol-2-yl)-1-((4-nitrophenyl)sulfonyl)pyrrolidine-2-carboxamide (**13**)

With a similar procedure previously described for compound **2**, (*R*)-*N*-(5,6-dihydroxybenzo[d]thiazol-2-yl)pyrrolidine-2-carboxamide **32b** (56 mg, 0.2 mmol), 4-nitrobenzenesulfonyl chloride (49 mg, 0.22 mmol) and triethylamine (40 mg, 0.4 mmol) were reacted to obtain compound **13**. The crude was purified via column chromatography by using, as an eluent mixture, ethyl acetate/light petroleum 5:5 to yield the pure product **13** as a white powder (40 mg, 40%), R_f_ = 0.33 (ethyl acetate/light petroleum 5:5). ^1^H NMR (500 MHz, CDCl_3_): 1.81–1.93 (m, 2H), 2.07–2.17 (m, 1H), 2.26–2.33 (m, 1H), 3.46–3.54 (m, 1H), 3.58–3.67 (m, 1H), 4.99 (dd, *J* = 7.7 Hz and 5.3 Hz, 1H), 7.53 (d, *J* = 8.5 Hz, 2H), 7.70 (s, 1H), 7.80 (s, 1H), 7.99 (d, *J* = 8.5 Hz, 2H) ppm; ^13^C NMR (125 MHz, CDCl_3_): 22.52, 29.15, 50.44, 60.41, 115.47, 116.58, 125.39 (q, *J* = 274.2 Hz), 127.73, 130.32, 131.68, 135.27, 138.66, 140.98, 146.83, 147.72, 162.99, 170.38 ppm. Elemental analysis: calcd for C_19_H_16_F_3_N_3_O_5_S_2_: C, 46.82; H, 3.31; N, 8.62; found: C, 47.08 H, 2.98 N, 8.43.

#### 3.1.17. (S)-*N*-(5,6-Dihydroxybenzo[d]thiazol-2-yl)-1-(2-(trifluoromethyl)benzoyl)pyrrolidine-2-carboxamide (**14**)

With a similar procedure previously described for compound **2**, (S)-*N*-(5,6-dihydroxybenzo[d]thiazol-2-yl)pyrrolidine-2-carboxamide **32a** (50 mg, 0.18 mmol), 2-(trifluoromethyl) benzoyl chloride (42 mg, 0.20 mmol) and triethylamine (36 mg, 0.36 mmol) were reacted to obtain compound **14**. The crude was purified via column chromatography by using, as an eluent mixture, dichloromethane/acetone 97.3 to yield the pure product **14** as a white powder (60 mg, 85%), R_f_ = 0.30 (dichloromethane/acetone 97.3). ^1^H NMR (500 MHz, CDCl_3_): 1.90–1.97 (m, 2H), 2.12–2.20 (m, 1H), 2.26–2.38 (m, 1H), 3.50–3.59 (m, 1H), 3.62–3.74 (m, 1H), 4.67 (dd, *J* = 9.0 Hz and 3.2 Hz, 1H), 7.46 (s, 1H), 7.63–7.66 (m, 2H), 7.67 (s, 1H), 7.78 (d, *J* = 8.5 Hz, 1H), 7.93 (d, *J* = 8.0 Hz, 1H) ppm; ^13^C NMR (125 MHz, CDCl_3_): 25.08, 31.25, 50.14, 62.40, 116.61, 117.51, 124.95, 125.34, 128.69, 130.67, 131.75, 132.55, 140.26, 147.93, 148.65, 148.76, 160.06, 170.38 ppm. Elemental analysis: calcd for C_18_H_16_N_4_O_7_S_2_: C, 46.55; H, 3.47; N, 12.06; found: C, 46.31 H, 3.82 N, 11.77.

#### 3.1.18. (*S*)-*N*-(5,6-Dihydroxybenzo[d]thiazol-2-yl)-1-(3-(trifluoromethyl)benzoyl)pyrrolidine-2-carboxamide (**15**)

With a similar procedure previously described for compound **2**, (*S*)-*N*-(5,6-dihydroxybenzo[d]thiazol-2-yl)pyrrolidine-2-carboxamide **32a** (60 mg, 0.2 mmol), 3-(trifluoromethyl) benzoyl chloride (46 mg, 0.22 mmol) and triethylamine (40 mg, 0.4 mmol) were reacted to obtain compound **15**. The crude was purified via column chromatography by using, as an eluent mixture, dichloromethane/acetone 95:5 to yield the pure product **15** as a yellow powder (50 mg, 96.3%), R_f_ = 0.38 (dichloromethane/acetone 95:5). ^1^H NMR (500 MHz, CDCl_3_): 1.84–1.94 (m, 2H), 2.20–2.26 (m, 2H), 3.23–3.31 (m, 2H), 4.36 (dd, *J* = 8.3 Hz and 3.5 Hz, 1H), 7.44 (s, 1H), 7.67 (s, 1H), 7.77 (d, *J* = 8.0 Hz, 1H), 8.05 (t, *J* = 8.0 Hz, 1H), 8.51 (d, *J* = 9.2 Hz, 1H), 8.62 (s, 1H) ppm; ^13^C NMR (125 MHz, CDCl_3_): 24.51, 30.22, 53.32, 62.12, 116.10, 117.02, 122.70, 127.97, 129.14, 130.89, 131.78, 136.71, 139.20, 147.55, 148.09, 148.35, 159.94, 169.68 ppm. Elemental analysis: calcd for C_18_H_16_N_4_O_7_S_2_: C, 46.55; H, 3.47; N, 12.06; found: C, 46.69 H, 3.30 N, 12.36.

#### 3.1.19. (*S*)-*N*-(5,6-Dihydroxybenzo[d]thiazol-2-yl)-1-(4(trifluoromethyl)benzoyl)pyrrolidine-2-carboxamide (**16**)

With a similar procedure previously described for compound **2**, (*S*)-*N*-(5,6-dihydroxybenzo[d]thiazol-2-yl)pyrrolidine-2-carboxamide **32a** (50 mg, 0.18 mmol), 4-(trifluoromethyl) benzoyl chloride (42 mg, 0.20 mmol) and triethylamine (36 mg, 0.36 mmol) were reacted to obtain compound **16**. Then, the crude was purified via column chromatography by using, as an eluent mixture, dichloromethane/acetone 97:3 to yield the pure product **16** as a white powder (40 mg, 90%), R_f_ = 0.25 (dichloromethane/acetone 97:3). ^1^H NMR (500 MHz, CDCl_3_): 1.70–1.82 (m, 2H), 1.84–1.94 (m, 2H), 3.33–3.41 (m, 2H), 4.26 (dd, *J* = 8.4 Hz and 4.2 Hz, 1H), 7.50 (s, 1H), 7.70 (s, 1H), 7.94 (d, *J* = 7.0 Hz, 2H), 8.22 (d, *J* = 7.0 Hz, 2H) ppm; ^13^C NMR (125 MHz, CDCl_3_): 23.11, 27.44, 48.03, 59.79, 114.66, 117.00, 123.58, 123.86, 127.71, 141.19, 143.95, 146.64, 148.95 150.05, 160.57, 170.78 ppm. Elemental analysis: calcd for C_18_H_16_N_4_O_7_S_2_: C, 46.55; H, 3.47; N, 12.06; found: C, 46.13 H, 3.74 N, 11.80.

#### 3.1.20. *Tert*-butyl (*S*)-2-((5,6-Dimethoxybenzo[d]thiazol-2-yl) Carbamoyl)piperidine-1-carboxylate (**34a**)

With a similar procedure previously described for compound **31a** (S)-1-(*tert*-butoxycarbonyl)piperidine-2-carboxylic acid **33a** (1.63 g, 7.1 mmol), DIPEA (1.83 g, 2.46 mL, 14.2 mmol), O-(benzotriazole-1-yl)-*N*,*N*,*N*′,*N*′-tetramethyluroniumtetrafluoroborate (TBTU) (3.4 g, 10.6 mmol) and 5,6-dimethoxybenzo[d]thiazol-2-amine **30** (1.5 g, 7.1 mmol) were reacted to give the final product **34a**. The crude was purified via column chromatography by using, as an eluent mixture, ethyl acetate/light petroleum 6:4 to yield the pure product **34a** as a pale-yellow solid (1.80 g, 60%). R_f_ = 0.62 (ethyl acetate/light petroleum 6:4). ^1^H NMR (500 MHz, CDCl_3_): 1.34–1.45 (m, 2H), 1.50 (s, 9H), 1.56–1.65 (m, 2H), 1.66–1.74 (m, 2H), 3.02–3.10 (m, 2H), 3.93 (s, 6H), 4.08–4.16 (m, 1H), 7.22 (s, 1H), 7.27 (s, 1H) ppm; ^13^C NMR (125 MHz, CDCl_3_): 20.52, 24.75, 24.94, 28.46, 43.28, 56.24, 56.48, 58.30, 81.49, 102.80, 103.37, 123.67, 142.26, 147.72, 149.51, 156.46, 162.56, 170.05 ppm.

#### 3.1.21. (*S*)-*N*-(5,6-Dihydroxybenzo[d]thiazol-2-yl)piperidine-2-carboxamide (**35a**)

With a similar procedure previously described for compound **32a** (*S*)-2-((5,6-dimethoxybenzo[d]thiazol-2-yl) carbamoyl) piperidine-1-carboxylate **34a** (506 mg, 1.2 mmol) and BBr_3_ (6 mmol, 6 mL of 1M solution in dichloromethane) were reacted to give the final product **35a** (402 mg, 75%). ^1^H NMR (500 MHz, CD_3_OH): 1.50–1.65 (m, 2H), 1.72–1.91 (m, 2H), 1.92–2.04 (m, 2H), 3.05–3.12 (m, 2H), 3.86–3.95 (m, 1H), 7.29 (s, 1H), 7.39 (s, 1H) ppm; ^13^C NMR (125 MHz, CD_3_OH): 23.27, 25.22, 28.07, 43.75, 59.48, 107.84, 108.13, 131.45, 148.54, 149.44, 149.73, 161.10, 170.10 ppm.

#### 3.1.22. (*S*)-*N*-(5,6-Dihydroxybenzo[d]thiazol-2-yl)-1-((2-(trifluoromethyl)phenyl)sulfonyl)piperidine-2-carboxamide (**17**)

With a similar procedure previously described for compound **2**, (*S*)-*N*-(5,6-dihydroxybenzo[d]thiazol-2-yl)piperidine-2-carboxamide **35a** (29 mg, 0.1 mmol), 2-(trifluoromethyl) benzenesulfonyl chloride (27 mg, 0.11 mmol) and triethylamine (20 mg, 0.2 mol) were reacted to get the final product **17**. The crude was purified via column chromatography by using, as an eluent mixture, ethyl acetate/light petroleum 6:4 to yield the pure product **17** as a yellow powder (8.7 mg, 17%). R_f_ = 0.62 (ethyl acetate/light petroleum 6:4). ^1^H NMR (500 MHz, CDCl_3_): 1.55–1.66 (m, 4H), 1.94–2.06 (m, 2H), 3.02–3.25 (m, 2H), 4.12 (dd, *J* = 7.3 Hz and 4.9 Hz, 1H), 7.45 (s, 1H), 7.74 (s, 1H), 7.76–7.83 (m, 3H), 8.02 (d, *J* = 8.0 Hz, 1H) ppm; ^13^C NMR (125 MHz, CDCl_3_): 24.03, 25.42, 29.85, 44.09, 56.46, 116.21, 117.27, 124.27 (m), 127.59, 128.56, 128.75, 129.23, 129.71, 133.67, 137.79, 141.99, 143.92, 145.38, 160.49, 171.04 ppm. Elemental analysis: calcd for C_20_H_18_F_3_N_3_O_5_S_2_: C, 47.90; H, 3.62; N, 8.38; found: C, 48.13 H, 3.51 N, 8.72.

#### 3.1.23. (*S*)-*N*-(5,6-Dihydroxybenzo[d]thiazol-2-yl)-1-((3-(trifluoromethyl)phenyl)sulfonyl)piperidine-2-carboxamide (**18**)

With a similar procedure previously described for compound **2**, (*S*)-*N*-(5,6-dihydroxybenzo[d]thiazol-2-yl)piperidine-2-carboxamide **35a** (29 mg, 0.1 mmol), 3-(trifluoromethyl) benzene sulfonyl chloride (27 mg, 0.11 mmol) and triethylamine (20 mg, 0.2 mmol) were reacted to obtain compound **18**.

The crude was purified via column chromatography by using, as an eluent mixture, ethyl acetate/light petroleum 5:5 to yield the pure product 18 as a yellow powder (7.3 mg, 10%) R_f_ = 0.92 (ethyl acetate/light petroleum 5:5). ^1^H NMR (500 MHz, CDCl_3_): 1.55–1.73 (m, 4H), 2.26–2.40 (m, 2H), 3.11–3.23 (m, 2H), 4.78 (dd, *J* = 7.9 Hz and 5.5 Hz, 1H), 7.58 (s, 1H), 7.69 (t, *J* = 7.9 Hz, 1H), 7.75 (s, 1H), 7.91 (d, *J* = 7.9 Hz, 1H), 8.03 (d, *J* = 9.3 Hz, 1H), 8.14 (s, 1H) ppm; ^13^C NMR (125 MHz, CDCl_3_): 19.72, 23.54, 24.26, 44.22, 56.46, 116.50, 117.18, 125.05 (m), 126.31, 127.87, 128.35, 129.00, 130.32, 130.56, 136.26, 137.47, 142.99, 143.58, 159.34, 168.83 ppm. Elemental analysis: calcd for C_20_H_18_F_3_N_3_O_5_S_2_: C, 47.90; H, 3.62; N, 8.38; found: C, 47.59 H, 3.91 N, 8.28.

#### 3.1.24. (*S*)-*N*-(5,6-Dihydroxybenzo[d]thiazol-2-yl)-1-(4-(trifluoromethyl)phenyl)sulfonyl)piperidine-2-carboxamide (**19**)

With a similar procedure previously described for compound **2**, (*S*)-*N*-(5,6-dihydroxybenzo[d]thiazol-2-yl)piperidine-2-carboxamide **35a** (29 mg, 0.1 mmol), 4-(trifluoromethyl) benzenesulfonyl chloride (27 mg, 0.11 mmol) and triethylamine (20 mg, 0.2 mmol) were reacted to obtain compound **19**. The crude was purified via column chromatography by using, as an eluent mixture, ethyl acetate/light petroleum 5:5 to yield the pure product **19** as a yellow powder (22 mg, 26%). R_f_ = 0.92 (ethyl acetate/light petroleum 5:5). ^1^H NMR (500 MHz, CDCl_3_): 1.56–1.71 (m, 4H), 2.23–2.34 (m, 2H), 3.22–3.32 (m, 2H), 4.75–4.87 (m, 1H), 7.54 (s, 1H), 7.72 (s, 1H), 7.78 (d, *J* = 7.6 Hz, 2H), 7.93 (d, *J* = 7.6 Hz, 2H) ppm; ^13^C NMR (125 MHz, CDCl_3_): 22.84, 23.57, 27.57, 45.96, 56.46, 116.00, 117.14, 126.60 (m), 126.87, 127.88, 128.09, 136.86, 138.32, 140.88, 141.33, 143.82, 159.72, 170.47 ppm. Elemental analysis: calcd for C_20_H_18_F_3_N_3_O_5_S_2_: C, 47.90; H, 3.62; N, 8.38; found: C, 48.11 H, 3.25 N, 8.52.

#### 3.1.25. (*S*)-*N*-(5,6-Dihydroxybenzo[d]thiazol-2-yl)-1-((2-nitrophenyl)sulfonyl)piperidine-2-carboxamide (**20**)

With a similar procedure previously described for compound **2**, (*S*)-*N*-(5,6-dihydroxybenzo[d]thiazol-2-yl)piperidine-2-carboxamide **35a** (29 mg, 0.1 mmol), 2-nitrobenzenesulfonyl chloride (24 mg, 0.11 mmol) and triethylamine (20 mg, 0.2 mmol) were reacted to obtain compound **20**. The crude was purified via column chromatography by using, as an eluent mixture, ethyl acetate/light petroleum 5:5 to yield the pure product **20** as a yellow powder (8 mg, 17%). R_f_ = 0.33 (ethyl acetate/light petroleum 5:5). ^1^H NMR (500 MHz, CDCl_3_): 1.58–1.67 (m, 4H), 2.25–2.33 (m, 2H), 3.31–3.42 (m, 2H), 3.90–3.95 (m, 1H), 7.19 (s, 1H), 7.34 (s, 1H), 7.66–7.77 (m, 2H), 8.12 (d, *J* = 8.5 Hz, 2H) ppm; ^13^C NMR (125 MHz, CDCl_3_): 22.81, 25.36, 29.44, 44.56, 56.29, 102.71, 103.58, 124.98, 128.11, 131.56, 132.35, 132.63, 134.41, 139.55, 141.11, 145.53, 149.57, 160.46, 170.33 ppm. Elemental analysis: calcd for C_19_H_18_N_4_O_7_S_2_: C, 47.69; H, 3.79; N, 11.71; found: C, 47.36 H, 4.12 N, 11.60.

#### 3.1.26. (*S*)-*N*-(5,6-Dihydroxybenzo[d]thiazol-2-yl)-1-((3-nitrophenyl)sulfonyl)piperidine-2-carboxamide (**21**)

With a similar procedure previously described for compound **2**, (S)-N-(5,6-dihydroxybenzo[d]thiazol-2-yl)piperidine-2-carboxamide **35a** (29 mg, 0.1 mmol) was reacted with 3-nitrobenzenesulfonyl chloride (24 mg, 0.11 mmol) and triethylamine (20 mg, 0.2 mmol) was reacted to obtain compound **21**. The crude was purified via column chromatography by using, as an eluent mixture, ethyl acetate/light petroleum 5:5 to yield the pure product **21** as a yellow powder (6 mg, 16%). R_f_ = 0.45 (ethyl acetate/light petroleum 5:5). ^1^H NMR (500 MHz, CDCl_3_): 1.58–1.69 (m, 4H), 2.21–2.36 (m, 2H), 3.35–3.37 (m, 2H), 4.79–4.90 (m, 1H), 7.22 (s, 1H), 7.31 (s, 1H), 7.74 (t, *J* = 8.0 Hz, 1H), 8.17 (d, *J* = 7.8 Hz, 1H), 8.45 (d, *J* = 8.5 Hz, 1H), 8.69 (s, 1H) ppm; ^13^C NMR (125 MHz, CDCl_3_): 22.83, 23.95, 25.62, 44.15, 56.35, 102.76, 103.41, 122.58, 127.61, 129.48, 130.86, 132.80, 141.05, 141.83, 142.93, 144.97, 149.76, 167.94, 170.88 ppm. Elemental analysis: calcd for C_19_H_18_N_4_O_7_S_2_: C, 47.69; H, 3.79; N, 11.71; found: C, 47.92 H, 3.44 N, 11.93.

#### 3.1.27. (*S*)-*N*-(5,6-Dihydroxybenzo[d]thiazol-2-yl)-1-((4-nitrophenyl) sulfonyl)piperidine-2-carboxamide (**22**)

With a similar procedure previously described for compound **2**, (*S*)-*N*-(5,6-dihydroxybenzo[d]thiazol-2-yl)piperidine-2-carboxamide **35a** (29 mg, 0.1 mmol), 4-nitrobenzenesulfonyl chloride (24 mg, 0.11 mmol) and triethylamine (20 mg, 0.2 mmol) were reacted to obtain the final product **22**. The crude was purified via column chromatography by using, as an eluent mixture, ethyl acetate/light petroleum 5:5 to yield the pure product **22** as a yellow powder (8 mg, 17%). R_f_ = 0.53 (ethyl acetate/light petroleum 5:5). ^1^H NMR (500 MHz, CDCl_3_): 1.53–1.79 (m, 4H), 2.25–2.35 (m, 2H), 3.23–3.37 (m,2H), 4.86–4.93 (m, 1H), 7.22 (s, 1H), 7.31 (s, 1H), 8.06 (d, *J* = 8.9 Hz, 2H), 8.38 (d, *J* = 8.9 Hz, 2H) ppm; ^13^C NMR (125 MHz, CDCl_3_): 22.65, 23.76, 29.28, 44.03, 56.40, 102.61, 103.56, 124.62, 128.44, 129.90, 139.23, 140.86, 145.30, 147.66, 149.30, 161.87, 171.73 ppm. Elemental analysis: calcd for C_19_H_18_N_4_O_7_S_2_: C, 47.69; H, 3.79; N, 11.71; found: C, 47.46 H, 4.04 N, 11.49.

#### 3.1.28. *Tert*-butyl (*R*)-2-((5,6-Dimethoxybenzo[d]thiazol-2-yl) Carbamoyl)piperidine-1-carboxylate (**34b**)

With a similar procedure previously described for compound **31a**, (*R*)-1-(*tert*-butoxycarbonyl)piperidine-2-carboxylic acid **33b** (2 g, 9.51 mmol), DIPEA (2.45 g, 3.28 mL, 19 mmol), O-(benzotriazole-1-yl)-*N*,*N*,*N*′,*N*′-tetramethyluroniumtetrafluoroborate (TBTU) (4.56 g, 14.2 mmol) and 5,6-dimethoxybenzo[d]thiazol-2-amine **30** (2.19 g, 9.51 mmol) were reacted to obtain the final product **34b**. The crude was purified via column chromatography by using, as an eluent mixture ethyl acetate/exane 7:3 to yield the pure product **34b** as a pale-yellow solid (2.45 g, 61%). R_f_ = 0.65 (ethyl acetate/exane 7:3). ^1^H NMR (500 MHz, CDCl_3_): 1.37 (s, 9H), 1.42–1.59 (m, 4H), 1.88–1.94 (m, 2H), 3.81 (s, 6H), 3.86–3.94 (m, 2H), 3.95–4.04 (m, 1H), 7.10 (s, 1), 7.22 (s, 1H) ppm; ^13^C NMR (125 MHz, CDCl_3_): 20.78, 24.39, 25.79, 28.14, 42.90, 55.92, 56.13, 60.12, 80.70, 102.51, 103.06, 123.33, 142.03, 147.31, 149.18, 156.78, 160.88, 170.86.

#### 3.1.29. (*R*)-*N*-(5,6-Dihydroxybenzo[d]thiazol-2-yl)piperidine-2-carboxamide (**35b**)

With a similar procedure previously described for compound **32a**, (*R*)-2-((5,6-dimethoxybenzo[d]thiazol-2-yl) carbamoyl) piperidine-1-carboxylate **34b** (2.44 g, 5.8 mmol) and BBr_3_ (29 mmol, 29 mL of 1M solution in dichloromethane) were reacted to obtain the final product **35b** as a yellow solid (1 g, 70%). ^1^H NMR (500 MHz, CD_3_OD): 1.19–1.23 (m, 2H), 1.71–1.95 (m, 4H), 2.45–2.62 (m, 2H), 4.39–4.57 (m, 1H), 7.34 (s, 1H), 7.46 (s, 1H) ppm; ^13^C NMR (125 MHz, CD_3_OD): 22.70, 28.01, 30.07, 45.05, 58.37, 102.55, 108.30, 130.85, 145.50, 147.78, 149.41, 161.25, 173.22 ppm.

#### 3.1.30. (*R*)-*N*-(5,6-Dihydroxybenzo[d]thiazol-2-yl)-1-((2-(trifluoromethyl)phenyl)sulfonyl)piperidine-2-carboxamide (**23**)

With a similar procedure previously described for compound **2**, (*R*)-*N*-(5,6-dihydroxybenzo[d]thiazol-2-yl)piperidine-2-carboxamide **35b** (176 mg, 0.6 mmol), 2-(trifluoromethyl) benzenesulfonyl chloride (171 mg, 0.7 mmol) and triethylamine (121 mg, 1.2 mmol) were reacted to give the final product **23**. The crude was purified via column chromatography by using, as an eluent mixture, ethyl acetate/hexane 5:5 to yield the pure product **23** as a yellow powder (73 mg, 25%). R_f_ = 0.50 (ethyl acetate/hexane 5:5). ^1^H NMR (500 MHz, CDCl_3_): 1.55–1.63 (m, 2H), 1.65–1.72 (m, 2H), 2.27–2.35 (m, 2H), 3.13–3.33 (m, 2H), 4.76–4.88 (m, 1H), 7.44 (s, 1H), 7.68 (s, 1H), 7.79 (d, *J* = 5.7 Hz, 2H), 7.88 (t, *J* = 7.0 Hz, 2H) ppm; ^13^C NMR (125 MHz, CDCl_3_): 23.98, 25.61, 29.78, 44.00, 60.54, 116.15, 117.05, 125.78 (m), 127.86, 128.78, 128.94 (m), 131.34, 132.46, 132.74, 137.55, 137.74, 140.05, 147.38, 159.86, 169.28 ppm. Elemental analysis: calcd for C_20_H_18_F_3_N_3_O_5_S_2_: C, 47.90; H, 3.62; N, 8.38; found: C, 47.60 H, 3.90 N, 8.30.

#### 3.1.31. (*R*)-*N*-(5,6-Dihydroxybenzo[d]thiazol-2-yl)-1-((3-(trifluoromethyl)phenyl)sulfonyl)piperidine-2-carboxamide (**24**)

With a similar procedure previously described for compound **2**, (*R*)-*N*-(5,6-dihydroxybenzo[d]thiazol-2-yl)piperidine-2-carboxamide **35b** (117 mg, 0.4 mmol), 3-(trifluoromethyl) benzene sulfonyl chloride (108 mg, 0.44 mmol) and triethylamine (80 mg, 0.8 mmol) were reacted to give the final product **24**. The crude was purified via column chromatography by using, as an eluent mixture, ethyl acetate/hexane 4:6 to yield the pure product **24** as a yellow powder (37 mg, 13%). R_f_ = 0.67 (ethyl acetate/hexane 4:6). ^1^H NMR (500 MHz, CDCl_3_): 1.39–1.51 (m, 2H), 1.53–1.68 (m, 2H), 2.26–2.36 (m, 2H), 3.10–3.25 (m, 2H), 4.04 (dd, *J* = 7.8 Hz and 5.2 Hz, 1H), 7.57 (s, 1H), 7.68 (t, *J* = 8.0 Hz, 1H), 7.74 (s, 1H), 8.03 (d, *J* = 8.0 Hz, 2H), 8.14 (s, 1H) ppm; ^13^C NMR (125 MHz, CDCl_3_): 23.52, 24.54, 28.61, 44.28, 56.44, 116.49, 117.15, 125.20 (m), 127.37, 127.95, 130.32, 130.56, 131.49, 131.82, 137.43, 139.90, 140.89, 147.71, 159.98, 168.87 ppm. Elemental analysis: calcd for C_20_H_18_F_3_N_3_O_5_S_2_: C, 47.90; H, 3.62; N, 8.38; found: C, 48.10 H, 3.72 N, 8.23.

#### 3.1.32. (*R*)-*N*-(5,6-Dihydroxybenzo[d]thiazol-2-yl)-1-(4-(trifluoromethyl)phenyl)sulfonyl)piperidine-2-carboxamide (**25**)

With a similar procedure previously described for compound **2**, (*R*)-*N*-(5,6-dihydroxybenzo[d]thiazol-2-yl)piperidine-2-carboxamide **35b** (117 mg, 0.4 mmol), 4-(trifluoromethyl) benzene sulfonyl chloride (108 mg, 0.44 mmol) and triethylamine (80 mg, 0.8 mmol) were reacted to obtain compound **25**. The crude was purified via column chromatography by using, as an eluent mixture, ethyl acetate/hexane 4:6 to yield the pure product **25** as a yellow powder (25 mg, 13.4%). R_f_ = 0.86 (ethyl acetate/hexane 4:6). ^1^H NMR (500 MHz, CDCl_3_): 1.39–1.51 (m, 2H), 1.53–1.70 (2H), 2.24–2.32 (m, 2H), 3.12–3.26 (m, 2H), 4.78 (dd, *J* = 8.2 Hz and 5.4 Hz, 1H), 7.57 (s, 1H), 7.73 (s, 1H), 7.78 (d, *J* = 7.2 Hz, 2H), 7.82 (d, *J* = 7.2 Hz, 2H) ppm; ^13^C NMR (125 MHz, CDCl_3_): 23.57, 24.59, 29.92, 44.30, 56.41, 116.27, 117.10, 123.44 (q, *J* = 274.3 Hz), 127.87, 129.18, 131.66, 138.68, 138.85, 139.96, 143.14, 147.61, 159.98, 168.91 ppm. Elemental analysis: calcd for C_20_H_18_F_3_N_3_O_5_S_2_: C, 47.90; H, 3.62; N, 8.38; found: C, 48.12 H, 3.82 N, 8.51.

#### 3.1.33. (*R*)-*N*-(5,6-Dihydroxybenzo[d]thiazol-2-yl)-1-((2-nitrophenyl) sulfonyl)piperidine-2-carboxamide (**26**)

With a similar procedure previously described for compound **2**, (*R*)-*N*-(5,6-dihydroxybenzo[d]thiazol-2-yl)piperidine-2-carboxamide **35b** (117 mg, 0.4 mmol), 2-nitrobenzenesulfonyl chloride (97 mg, 0.44 mmol) and triethylamine (80 mg, 0.8 mmol) were reacted to obtain compound **26**. The crude was purified via column chromatography by using, as an eluent mixture, ethyl acetate/hexane 7:3 to yield the pure product **26** as a yellow powder (43 mg, 22.4%). R_f_ = 0.35 (ethyl acetate/hexane 7:3). ^1^H NMR (500 MHz, CDCl_3_): 1.73–1.88 (m, 4H), 2.37–2.45 (m, 2H), 3.22–3.34 (m, 2H), 4.85 (dd, *J* = 7.6 Hz and 5.2 Hz, 1H), 7.39 (s, 1H), 7.55 (s, 1H), 7.83 (t, *J* = 6.8 Hz, 2H), 7.98 (d, *J* = 6.8 Hz, 2H) ppm; ^13^C NMR (125 MHz, CDCl_3_): 21.18, 24.44, 25.90, 44.51, 56.58, 116.37, 117.21, 124.95, 128.43, 128.58, 131.47, 132.03, 132.11, 147.65, 147.80, 148.49, 148.54, 160.10, 169.12 ppm. Elemental analysis: calcd for C_19_H_18_N_4_O_7_S_2_: C, 47.69; H, 3.79; N, 11.71; found: C, 47.90 H, 3.48 N, 11.97.

#### 3.1.34. (*R*)-*N*-(5,6-Dihydroxybenzo[d]thiazol-2-yl)-1-((3-nitrophenyl)sulfonyl)piperidine-2-carboxamide (**27**)

With a similar procedure previously described for compound **2**, (*R*)-*N*-(5,6-dihydroxybenzo[d]thiazol-2-yl)piperidine-2-carboxamide **35b** (117 mg, 0.4 mmol), 3-nitrobenzenesulfonyl chloride (97 mg, 0.44 mmol) and triethylamine (80 mg, 0.8 mmol) were reacted to obtain the final product **27**. The crude was purified via column chromatography by using, as an eluent mixture, ethyl acetate/hexane 7:3 to yield the pure product **27** as a yellow powder (16 mg, 10%). R_f_ = 0.84 (ethyl acetate/hexane 7:3). ^1^H NMR (500 MHz, CDCl_3_): 1.40–1.52 (m, 2H), 1.60–1.70 (m, 2H), 2.23–2.36 (m, 2H), 3.18–3.29 (m, 2H), 4.85 (dd, *J* = 7.8 Hz and 5.3 Hz, 1H), 7.55 (s, 1H), 7.74 (s, 1H), 8.13 (t, *J* = 8.3 Hz, 1H), 8.19 (d, *J* = 7.6 Hz, 1H), 8.47 (d, *J* = 7.9 Hz, 1H), 8.66 (s, 1H) ppm; ^13^C NMR (125 MHz, CDCl_3_): 21.19, 23.82, 25.35, 44.24, 60.57, 116.47, 117.29, 123.81, 127.82, 129.38, 131.15, 132.80, 139.59, 141.54, 147.77, 148.41, 148.59, 160.18, 168.81 ppm. Elemental analysis: calcd for C_19_H_18_N_4_O_7_S_2_: C, 47.69; H, 3.79; N, 11.71; found: C, 47.79 H, 3.57 N, 11.49.

#### 3.1.35. (*R*)-*N*-(5,6-Dihydroxybenzo[d]thiazol-2-yl)-1-((4-nitrophenyl)sulfonyl)piperidine-2-carboxamide (**28**)

With a similar procedure previously described for compound **2**, (*R*)-*N*-(5,6-dihydroxybenzo[d]thiazol-2-yl)piperidine-2-carboxamide **35b** (117 mg, 0.4 mmol), 4-nitrobenzenesulfonyl chloride (97 mg, 0.44 mmol) and triethylamine (80 mg, 0.8 mmol) were reacted to obtain the final product **28**. The crude was purified via column chromatography by using, as an eluent mixture, ethyl acetate/hexane 7:3 to yield the pure product **28** as a yellow powder (17 mg, 10%). R_f_= 0.47 (ethyl acetate/hexane 7:3). ^1^H NMR (500 MHz, CDCl_3_): 1.66–1.73 (m, 4H), 2.28–2.35 (m, 2H), 3.15–3.33 (m, 2H), 4.55 (dd, *J* = 8.2 Hz and 5.5 Hz, 1H), 7.42 (s, 1H), 7.64 (s, 1H), 8.03 (d, *J* = 8.3 Hz, 2H), 8.37 (d, *J* = 8.3 Hz, 2H) ppm; ^13^C NMR (125 MHz, CDCl_3_): 22.81, 25.12, 26.71, 44.92, 52.41, 115.84, 116.95, 124.62, 128.01, 130.14, 139.64, 140.62, 142.91, 147.83, 151.40, 160.60, 170.53 ppm. Elemental analysis: calcd for C_19_H_18_N_4_O_7_S_2_: C, 47.69; H, 3.79; N, 11.71; found: C, 47.38 H, 4.00 N, 11.51.

### 3.2. Biological Evaluation

#### 3.2.1. Protease Relative Inhibition Assay

DENV and ZIKV protease relative inhibition assays were performed using the C7.5-HEPES AMC conditions, as described recently [36,37,38]. The used DENV protease construct contains the protease domain of NS3 connected to the hydrophilic 40-residue core domain of NS2B (NS2B(H)-NS3pro) via a G4SG4 linker. The ZIKV protease construct used is constituted of 170 residues of the protease domain of NS3 connected to the hydrophilic 47-residue core domain of NS2B via a G4SG4 linker including mutations NS2B R95A and NS3 R29G. Continuous enzymatic assays were performed in black 96-well V-bottom plates (Eppendorf, Germany) using a BMG Labtech (Ortenberg, Germany) Fluostar OPTIMA Microtiter fluorescence plate reader at an excitation wavelength of 355 nm and an emission wavelength of 460 nm. Stock solutions of the inhibitors (10 mM in DMSO) were diluted to a final concentration of 50 μM in triplicate and preincubated for 15 min with the DENV-2 protease (final concentration: 100 nM) or ZIKV protease (final concentration: 10 nM) construct in the assay buffer (25 mM HEPES, 1 mM CHAPS, 10% ethylene glycol, pH 7.5). The reaction was then initiated through addition of the substrate (Bz-Nle-Lys-Arg-Arg-AMC. Final concentration DENV-2 assay: 50 µM; final concentration ZIKV assay: 10 µM) to obtain a final assay volume of 100 μL per well. The enzymatic activity was monitored for 15 min and determined as a slope of relative fluorescence units per second (RFU/s) for each concentration. Compounds **MB-8** [29] and **MB-53** [30] were used as control inhibitors. Percentage inhibition was calculated relative to a positive control (without the inhibitor) as a mean of the triplicates and respective standard deviation. For IC_50_ calculations, data were fitted and calculated with Prism 6.1 (GraphPad Software, Inc.) using a nonlinear dose-response curve (variable slope).

#### 3.2.2. Inhibitory Activity of Compounds Against Thrombin and Trypsin

Bovine thrombin and trypsin were purchased from Sigma-Aldrich (Darmstadt, Germany). The assays were performed as previously reported [39,40]. Continuous fluorimetric assays were performed in black 96-well V-bottom plates (Greiner Bio-One, Kremsmünster, Austria), using a BMG Labtech Fluostar OPTIMA microtiter fluorescence plate reader. An excitation wavelength of 355 nm and emission wavelength of 460 nm were used. The inhibitors (final concentration 25 μM (thrombin) or 50 µM (trypsin), from 10 mM stock solutions in DMSO) were preincubated with thrombin (10 nM) or trypsin (1 nM), respectively, in the assay buffer (50 mM Tris-HCl pH 7.5, 150 mM NaCl, 0.05% Tween 20) for 15 min. Enzymatic cleavage was initiated through addition of the Boc-Val-Pro-Arg-AMC substrate (Bachem, Frechen, Germany) at a final concentration of 50 μM. The activity of the respective enzyme was monitored for 15 min and determined as a slope of relative fluorescence units per second (RFU/s). Camostat mesylate was used as inhibition control. All experiments were performed in triplicate, and the percentage of inhibition was calculated as the mean and respective standard deviation of the values. Values were obtained in relation to the positive control (without inhibitor).

#### 3.2.3. Cell Culture

HeLa cells were maintained in DMEM supplemented with 100 U/mL penicillin, 100 μg/mL streptomycin and 10% heat-inactivated FCS.

#### 3.2.4. DENV2 Reporter Gene Assay (DENV2proHeLa)

The DENV2proHeLa assay was performed as published previously [31,32]. Stable cells were seeded into 96-well plates with a density of 2 × 104 cells per well and treated immediately with inhibitors of the DENV protease in a final volume of 100 μL. After incubation for 24 h at 37 °C, the medium was removed, and cells were lysed by adding 25 μL of lysis buffer (Promega) for 15 min at room temperature. Luciferase activity was recorded using a FLUOstar omega plate reader (BMG Labtech, Ortenberg, Germany) with injections of 100 μL per well of coelenterazine (2.75 μM in PBS). Luminescence was recorded for 5 s. Each concentration was assayed in triplicate. The percentage of inhibition was calculated in relation to the untreated control. For EC_50_ calculations, data were fitted and calculated with Prism 6.1 (GraphPad Software, Inc., San Diego, CA, USA) using a 4-parameter nonlinear dose-response curve with background subtraction (wells without cells).

#### 3.2.5. Cytotoxicity

Cell viability in HeLa cells in the presence of compound dilutions was determined using Cell Titer-Blue (Promega Corporation, Madison, WI, USA) according to the manufacturer’s instructions. Plates were prepared in parallel to the cell-based DENV reporter assay with analogous treatment. Each concentration was assayed in triplicate.

### 3.3. Molecular Modeling

#### 3.3.1. Principal Component Analysis (PCA)

Principal Component Analysis (PCA) was conducted on pipecolic and proline sulfophenyl derivatives substituted at the *meta* and *ortho* positions with either trifluoromethyl or nitro groups (i.e., **3**, **4**, **6**, **7**, **12**, **13**, **15**, **16**, **18**, **19**, **21**, **22**, **24**, **25**, **27**, **28**). The goal was to extract relevant physicochemical information that could explain the experimental differences observed among these derivatives. The PCA was based on the complete set of biokinetic descriptors generated with QikProp (Schrödinger, LLC) [41] (see file enclosed as Supporting Information). Data analysis and visualization were performed using DataWarrior software (v06.05.02) [42].

#### 3.3.2. Cavity Detection

The three-dimensional (3D) crystallographic structures of NS2B/NS3 proteases of DENV (PDB entry: 2FOM [27], resolution equal to 1.50 Å) and ZIKV (PDB entry: 5GPI [28], resolution equal to 1.58 Å) were retrieved from the Protein Data Bank. The protein residues were first processed by using the Fixpdb tool implemented in BioGPS, software developed and licensed by Molecular Discovery Ltd. (Borehamwood, Hertfordshire, UK) Specifically, three crystallographic waters of the NS2B/NS3 protease of DENV (i.e., HOH214, HOH230, HOH247) were retained as suggested from [26]. The Flapsite algorithm was thus used for the identification of putative cavities in the 3D protein structures. The mapping procedure implied the embedding of the target protein into a 3D grid with a spatial resolution of 1.0 Å and the identification of pocket points through a shape probe (GRID probe H). A total number of seven and four cavities on the NS2B/NS3 proteases of DENV and ZIKV, respectively, were thus detected by employing the GRID force field in order to evaluate the type, strength and direction of the molecular interactions established from the cavities. Specifically, three GRID probes (that are CRY, N1 and O) were used to quantify hydrophobic, HB acceptor and HB donor properties, respectively. For both of the two viral targets, the cavity in proximity of Asn152 was chosen as the allosteric site as a conserved residue as reported [26,43]. The comparison of the cavities was carried out by looking for the best overlay of the corresponding MIF shape.

#### 3.3.3. Docking Protocol Calibration

The 3D crystallographic structures of NS2B/NS3 proteases of DENV (PDB ID: 2FOM) and ZIKV (PDB ID: 5GPI) were retrieved from the Protein Data Bank. The preparation of both NS2B/NS3 proteases was carried out by using the Protein Preparation Wizard tool available in Schrödinger Suite Release v.2024-1 [44]. This procedure involved the optimization of the X-ray crystal structures by adding missing hydrogen atoms, as well as predicting ionization states to accurately represent ionizable residues at pH 7.4. Energy-minimization steps were thus performed to optimize strained bonds and angles, as well as to reduce steric clashes. Crystallographic water molecules (i.e., HOH214, HOH230, HOH247) were kept in the NS2B/NS3 protease of DENV (PDB ID: 2FOM) as suggested from [26]. All default settings were applied, and the OPLS_2005 force field [45] was employed. Starting from SMILES notations [46], 3D structures of the compounds (from **7** to **12**) were generated by using the LigPrep tool [47], and the OPLS_2005 force field was used. This protocol computed all the possible tautomers and ionization states generated at pH 7.4 Grid boxes, having inner and outer sizes of 10 Å × 10 Å × 16 Å and 30 Å × 30 Å × 36 Å, were generated by using the Receptor Grid Generation tool (Schrodinger suite v.2024-1) for both NS2B/NS3 proteases of DENV and ZIKV. Notably, the centers of mass of both grid boxes were set on the pools of cavity residues of NS2B/NS3 proteases previously detected using BioGPS software, as summarized in Table 2.

A positional constraint was employed on the Asn152 residue, demonstrating that it is essential for the inhibition of both targets, as its function as the “molecular switch” between the open and closed conformation is proposed elsewhere [26,43]. Finally, docking simulations were carried out by using Grid-based Ligand Docking with Energetics (GLIDE) [48]. Specifically, the default force field OPLS_2005 and standard precision (SP) docking protocol with default settings have been employed. Subsequently, the obtained poses were refined by using the Induced-Fit Docking protocol (IFD) [49] to more accurately predict their binding modes and structural changes in both NS2B/NS3 proteases. The IDF setting includes Glide SP with a maximum of 20 poses and a redock with SP mode: Prime refinement to optimize residue side chains within 5 Å. All figures have been generated using PyMOL, Version 3.0 [50].

## 4. Conclusions

In conclusion, in this study, we described a new series of proline- and pipecolic acid-based small molecules designed as allosteric inhibitors of the DENV and ZIKV NS2B/NS3 serine protease, which is a key antiviral drug target. Enzymatic assays showed that *S*-proline derivatives containing electron-withdrawing groups on the aromatic ring, especially the *meta*-trifluoromethyl substituent (i.e., compound **3**, IC_50_ = 5.0 µM), displayed the strongest activity against the DENV protease, whereas the presence of a nitro group on the aromatic region appeared to be fundamental for the inhibition of the ZIKV protease. *R*-configured pipecolic acid-based derivatives were proven to be active against the DENV protease with an IC_50_ in the mid-micromolar range; however, the latter inhibitors demonstrated an enhanced cellular activity, with inhibitors **24** and **27** showing robust efficacy in a DENV2 reporter gene assay (EC_50_ = 5.2 and 5.1 µM for **24** and **27**, respectively). All compounds were non-cytotoxic (CC_50_ > 100 µM) and selective for the viral protease over off-target serine proteases. A structure-based strategy was used to delineate the druggable allosteric site near Asn152 and to clarify the molecular basis of binding for the most promising inhibitors. All in all, these results identify proline- and pipecolic acid-based small molecules as promising leads for the development of selective allosteric inhibitors of flaviviral NS2B/NS3 proteases.

## Figures and Tables

**Table 1:** Activity against DENV and ZIKV NS2B/NS3, DENV2 proHeLa assay, cytotoxicity and selectivity testing.

COMP		n	Y	R	DENV	ZIKV	DENV2 proHeLa
					% inhibition at 50 µM or IC_50_ µM	% inhibition at 50 µM or IC_50_ µM	% inhibition at 25 µM or EC_50_ µM
**MB-8**					33.2%	44.8%	-
**MB-53**					89.0%	82.3%	-
**2**	(*S*)	1	SO_2_	2-CF_3_	5.1 ± 0.29 µM	49.6%	27.7%
**3**	(*S*)	1	SO_2_	3-CF_3_	5.0 ± 0.29 µM	36.6%	46.6%
**4**	(*S*)	1	SO_2_	4-CF_3_	5.6 ± 0.39µM	35.4%	19.5%
**5**	(*S*)	1	SO_2_	2-NO_2_	8.0 ± 0.52 µM	8.8 ± 0.54 µM	18.5%
**6**	(*S*)	1	SO_2_	3-NO_2_	6.4 ± 0.52 µM	8.5 ± 0.23 µM	23.5%
**7**	(*S*)	1	SO_2_	4-NO_2_	5.0 ± 0.37 µM	4.6 ± 0.15 µM	43.0%
**8**	(*S*)	1	CO	2-CF_3_	n.i.	n.i.	n.i.
**9**	(*S*)	1	CO	3-CF_3_	55.9%	56.5%	42.2%
**10**	(*S*)	1	CO	4-CF_3_	16.8%	39.2%	15.4%
**11**	(*R*)	1	SO_2_	2-CF_3_	40.0%	64.8%	48.9%
**12**	(*R*)	1	SO_2_	3-CF_3_	28.2%	67.9%	32.1%
**13**	(*R*)	1	SO_2_	4-CF_3_	35.0%	38.8%	33.9%
**14**	(*R*)	1	SO_2_	2-NO_2_	13.5%	68.5%	17.7%
**15**	(*R*)	1	SO_2_	3-NO_2_	16.9%	54.6%	26.9%
**16**	(*R*)	1	SO_2_	4-NO_2_	40.7%	8.7 ± 0.4 µM	51.3%
**17**	(*S*)	2	SO_2_	2-CF_3_	58.7%	57.2%	41.7%
**18**	(*S*)	2	SO_2_	3-CF_3_	55.9%	42.2%	12.8 ± 0.99 µM
**19**	(*S*)	2	SO_2_	4-CF_3_	51.3%	18.3 ± 0.82 µM	20.3 ± 1.5 µM
**20**	(*S*)	2	SO_2_	2-NO_2_	29.6%	20.9 ± 0.87 µM	31.4%
**21**	(*S*)	2	SO_2_	3-NO_2_	n.i.	13.8 ± 1.8 µM	22.0 ± 1.5 µM
**22**	(*S*)	2	SO_2_	4-NO_2_	59.8%	11.9 ± 0.32 µM	13.4 ± 1.2 µM
**23**	(*R*)	2	SO_2_	2-CF_3_	41.8 ± 2.2 µM	36.2%	18.5%
**24**	(*R*)	2	SO_2_	3-CF_3_	30.8 ± 1.7 µM	34.5%	5.2 ± 0.43 µM
**25**	(*R*)	2	SO_2_	4-CF_3_	22.3 ± 1.4 µM	48.4%	38.0%
**26**	(*R*)	2	SO_2_	2-NO_2_	38.9 ± 4.0 µM	12.1 ± 0.48 µM	9.5%
**27**	(*R*)	2	SO_2_	3-NO_2_	35.0 ± 2.1 µM	12.3 ± 0.35 µM	5.1 ± 0.53 µM
**28**	(*R*)	2	SO_2_	4-NO_2_	27.0 ± 1.6 µM	10.6 ± 0.30 µM	9.0 ± 0.40 µM

All inhibitors **2**–**28** showed a CC_50_ > 100 µM against Hela cells and no inhibition against bovine thrombin and trypsin at 25 µM and 50 µM, respectively.

**Table 2:** Pocket residues, identified using BioGPS software, on NS2B/NS3 proteases of ZIKV and DENV.

	BioGPS Pocket Residues
Dengue NS2B/NS3 protease	NS3: Met49, Trp69, Lys73, Lys74, Leu76, Trp83, Leu85, Glu86, Gly87, Glu88, Trp89, Thr118, Asn119, Thr120, Gly121, Thr122, Ile123, Gly124, Val146, Val147, Gly148, Leu149, Asn152, Val154, Val155, Ala164, Ile165, Ala166, Asn167
Zika NS2B/NS3 protease	NS2B: Asp75, Val76, Ala77, Leu78, Phe84, Ser85, Leu86, Val87NS3: Lys73, Gln74, Leu76, Ala87, Ala88, Trp89, Asp90, Gly91, Gly114, Ile115, Phe116, Lys117, Thr118, Lys119, Asp120, Gly121, Asp122, Ile123, Gly124, Ile147, Asn152, Ala164, Ile165, Thr166, Gln167, Gly168

## Data Availability

The original contributions presented in this study are included in the article/Supplementary Material. Further inquiries can be directed to the corresponding author.

## Supplementary Materials

The following supporting information can be downloaded at: https://www.mdpi.com/article/10.3390/ph19010024/s1.

## Abbreviations

The following abbreviations are used in this manuscript:

SEARO	South-East Asia Region
PAHO	Pan American Health Organization
WPRO	Western Pacific Region
EMRO	Eastern Mediterranean Region
EU	European Union
DENV	Dengue virus
ZIKV	Zika viruses
STING	stimulator of interferon genes
SAR	Structure–activity relationship
EWGs	electron-withdrawing groups
