# Supplementary material for: Development of Novel Proline- and Pipecolic Acid-Based Allosteric Inhibitors of Dengue and Zika Virus NS2B/NS3 Protease
Source: Pharmaceuticals (Basel). 2025 Dec 22;19(1):24. doi: 10.3390/ph19010024 (PMC12845239; doi:10.3390/ph19010024)
Supplement: Supplementary file 1 [file pharmaceuticals-19-00024-s001.zip › pharmaceuticals-4034010-supplementary.pdf]

Supporting Information

Development of novel Proline- and Pipecolic acid-based Allosteric Inhibitors of Dengue and Zika Virus NS2B/NS3 Protease

Josè Starvaggi,[1] Carla Di Chio,[1] Johannes Lang,[2] Valentina Belgiovine,[3] Daniela Trisciuzzi,[3] Christian Klein,[2] Orazio Nicolotti,[3] Salvatore Di Maro,[4] Maria Zappalà[1] and Roberta Ettari[1],*

[1]Department of Chemical, Biological, Pharmaceutical and Environmental Sciences, University of Messina, Viale Ferdinando Stagno d'Alcontres 31, 98166 Messina, Italy.
[2]Institute of Pharmacy and Molecular Biotechnology, University of Heidelberg, Im Neuenheimer Feld 364, 69120 Heidelberg, Germany.
[3]Department of Pharmacy-Drug Sciences, University of Bari, via Amendola 173, I-70126 Bari, Italy.
[4]Department of Environmental, Biological and Pharmaceutical Science and Technology, University of Campania "Luigi Vanvitelli", Via A. Vivaldi, 43, 81100 Caserta, Italy

*Correspondence: rettari@unime.it; Tel.: +39-090-676-6554.

Table S1. IFD scores of **2**, **3**, **4**, **5**, **6**, 7 towards NS2B/NS3 proteases of Dengue and Zika virus, respectively.

	**IFD Score (kcal/mol)**	
**Compound**	**Dengue NS2B/NS3 protease**	**Zika NS2B/NS3 protease**
**2**	-7.484	-8.246
**3**	-6.753	-9.436
**4**	-7.391	-8.813
**5**	N/A	N/A
**6**	-7.781	-7.739
**7**	-7.590	-7.781

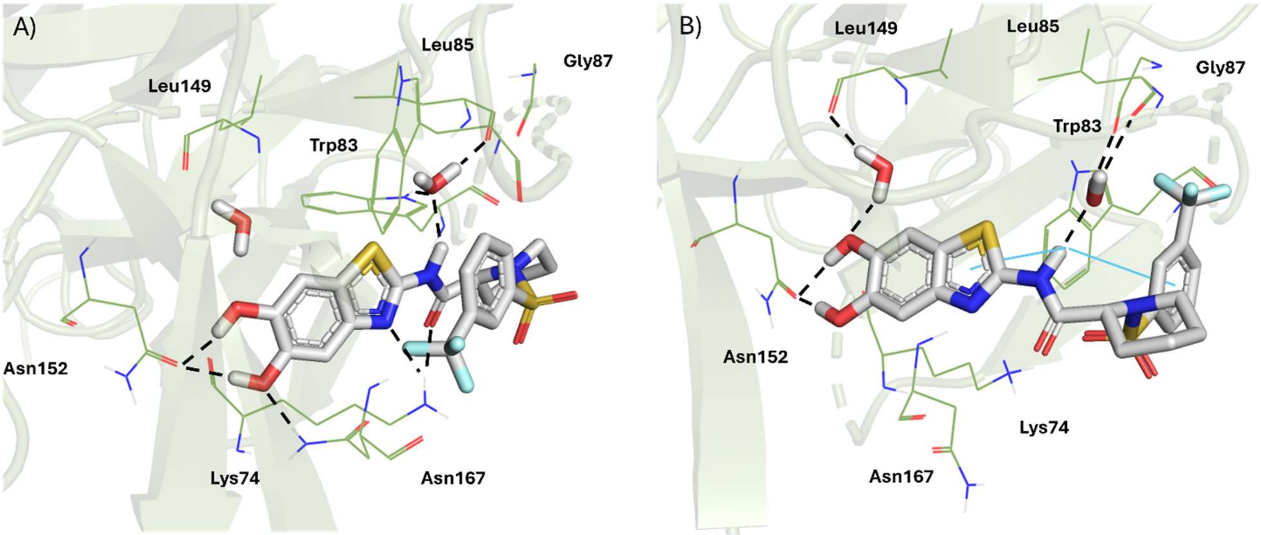

Figure S1. The panels (A) and (B) show the IFD poses of **18** and **24**, respectively, in white sticks, within the allosteric site of the NS2B/NS3 proteases of Dengue virus (PDB: 2FOM, green cartoon). Black dashed and cyan lines indicate HBs and π-π interaction, respectively.

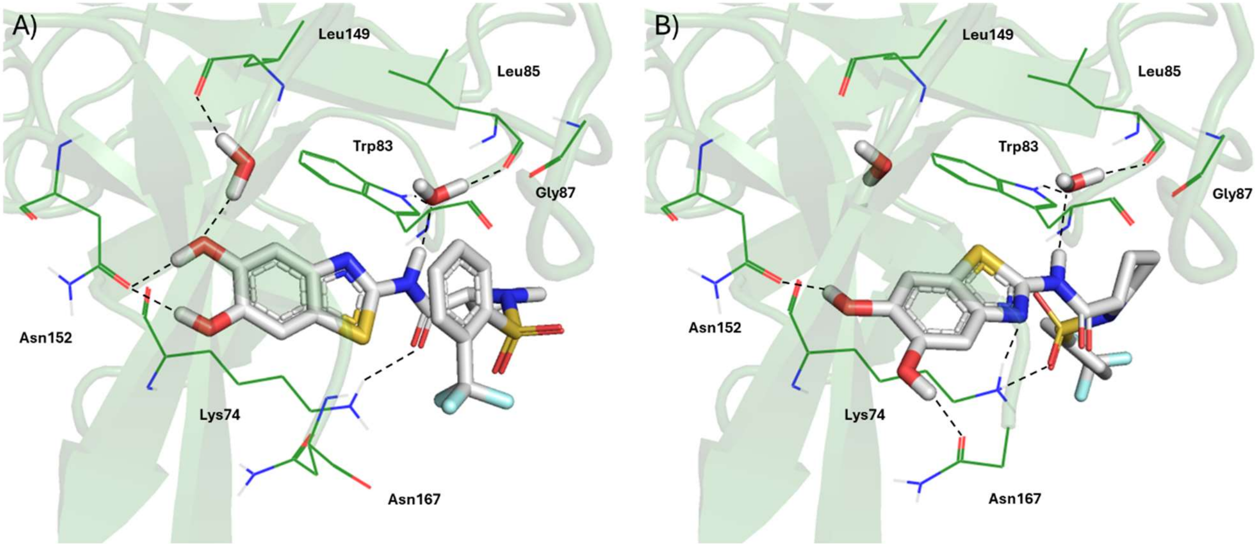

Figure S2. The panels (A) and (B) show the IFD poses of **2** and **4**, respectively, in white sticks, within the allosteric site of the NS2B/NS3 proteases of Dengue virus (PDB: 2FOM, green cartoon). Black dashed lines indicate HBs.

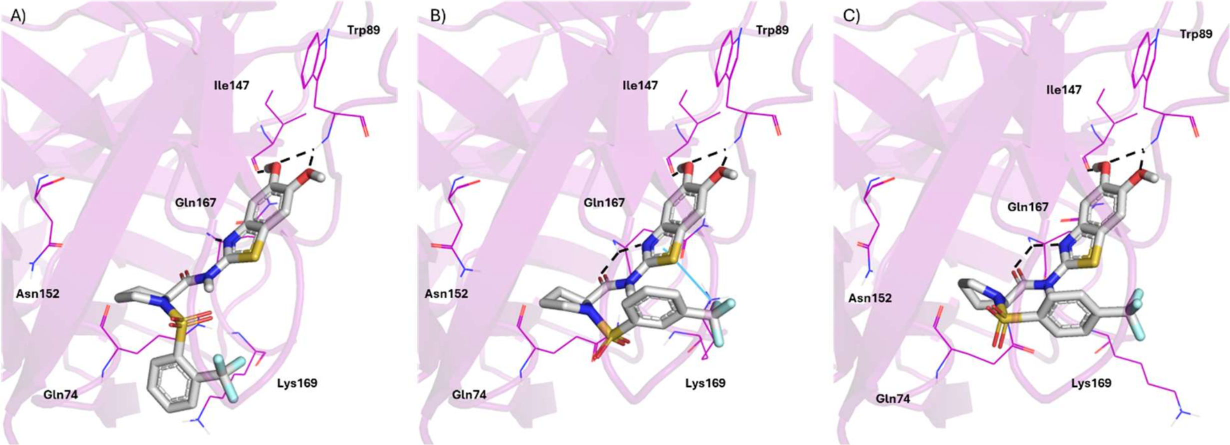

Figure S3. The panels (A), (B) and (C) show the IFD poses of **2**, **3** and **4** in white sticks within the allosteric site of the NS2B/NS3 protease of Zika virus (PDB: 5GPI, purple cartoon). Black dashed and cyan lines indicate HBs and π-π interaction, respectively.

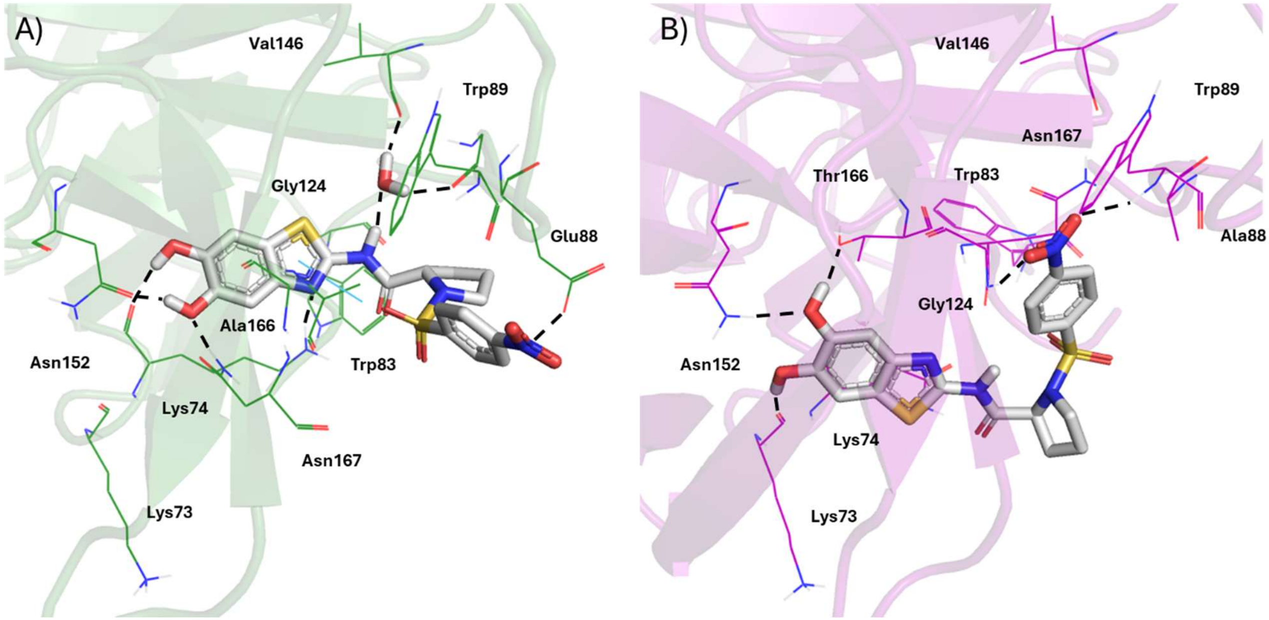

Figure S4. The panels (A) and (B) show the IFD poses of **7**, in white sticks within the allosteric pocket of NS2B/NS3 proteases of Dengue virus (PDB: 2FOM, green cartoon) and Zika virus (PDB: 5GPI, purple cartoon). Black dashed and cyan line indicate and π-π interaction, respectively.

Figure S5: ^{1}H NMR spectrum of compound **2**.

Figure S6: ^{13}C NMR spectrum of compound **2**.

Figure S7: ^{1}H NMR spectrum of compound **3**.

Figure S8: ^{13}C NMR spectrum of compound **3**.

Figure S9: ^{1}H NMR spectrum of compound **4**.

Figure S10: ^{13}C NMR spectrum of compound **4**.

Figure S11: ^{1}H NMR spectrum of compound **5**.

Figure S12: ^{13}C NMR spectrum of compound **5**.

Figure S13: ^{1}H NMR spectrum of compound **6**.

Figure S14: ^{13}C NMR spectrum of compound **6**.

Figure S15: ^{1}H NMR spectrum of compound **7**.

Figure S16: ^{13}C NMR spectrum of compound **7**.

Figure S17: ^{1}H NMR spectrum of compound **8**.

Figure S18: ^{13}C NMR spectrum of compound **8**.

Figure S19: ^{1}H NMR spectrum of compound **9**.

Figure S20: ^{13}C NMR spectrum of compound **9**.

Figure S21: ^{1}H NMR spectrum of compound **10**.

Figure S22: ^{13}C NMR spectrum of compound **10.**

Figure S23: ^{1}H NMR spectrum of compound **11**.

Figure S24: ^{13}C NMR spectrum of compound **11.**

Figure S25: ^{1}H NMR spectrum of compound **12**.

Figure S26: ^{13}C NMR spectrum of compound **12.**

Figure S27: ^{1}H NMR spectrum of compound **13**.

Figure S28: ^{13}C NMR spectrum of compound **13.**

Figure S29: ^{1}H NMR spectrum of compound **14**.

Figure S30: ^{13}C NMR spectrum of compound **14.**

Figure S31: ^{1}H NMR spectrum of compound **15**.

Figure S32: ^{13}C NMR spectrum of compound **15.**

Figure S33: ^{1}H NMR spectrum of compound **16**.

Figure S34: ^{13}C NMR spectrum of compound **16.**

Figure S35: ^{1}H NMR spectrum of compound **17**.

Figure S36: ^{13}C NMR spectrum of compound **17.**

Figure S37: ^{1}H NMR spectrum of compound **18**.

Figure S38: ^{13}C NMR spectrum of compound **18.**

Figure S39: ^{1}H NMR spectrum of compound **19**.

Figure S40: ^{13}C NMR spectrum of compound **19.**

Figure S41: ^{1}H NMR spectrum of compound **20**.

Figure S42: ^{13}C NMR spectrum of compound **20.**

Figure S43: ^{1}H NMR spectrum of compound **21**.

Figure S44: ^{13}C NMR spectrum of compound **21.**

Figure S45: ^{1}H NMR spectrum of compound **22**.

Figure S46: ^{13}C NMR spectrum of compound **22.**

Figure S47: ^{1}H NMR spectrum of compound **23**.

Figure S48: ^{13}C NMR spectrum of compound **23.**

Figure S49: ^{1}H NMR spectrum of compound **24**.

Figure S50: ^{13}C NMR spectrum of compound **24.**

Figure S51: ^{1}H NMR spectrum of compound **25**.

Figure S52: ^{13}C NMR spectrum of compound **25.**

Figure S53: ^{1}H NMR spectrum of compound **26**.

Figure S54: ^{13}C NMR spectrum of compound **26.**

Figure S55: ^{1}H NMR spectrum of compound **27**.

Figure S56: ^{13}C NMR spectrum of compound **27.**

Figure S57: ^{1}H NMR spectrum of compound **28**.

Figure S58: ^{13}C NMR spectrum of compound **28.**

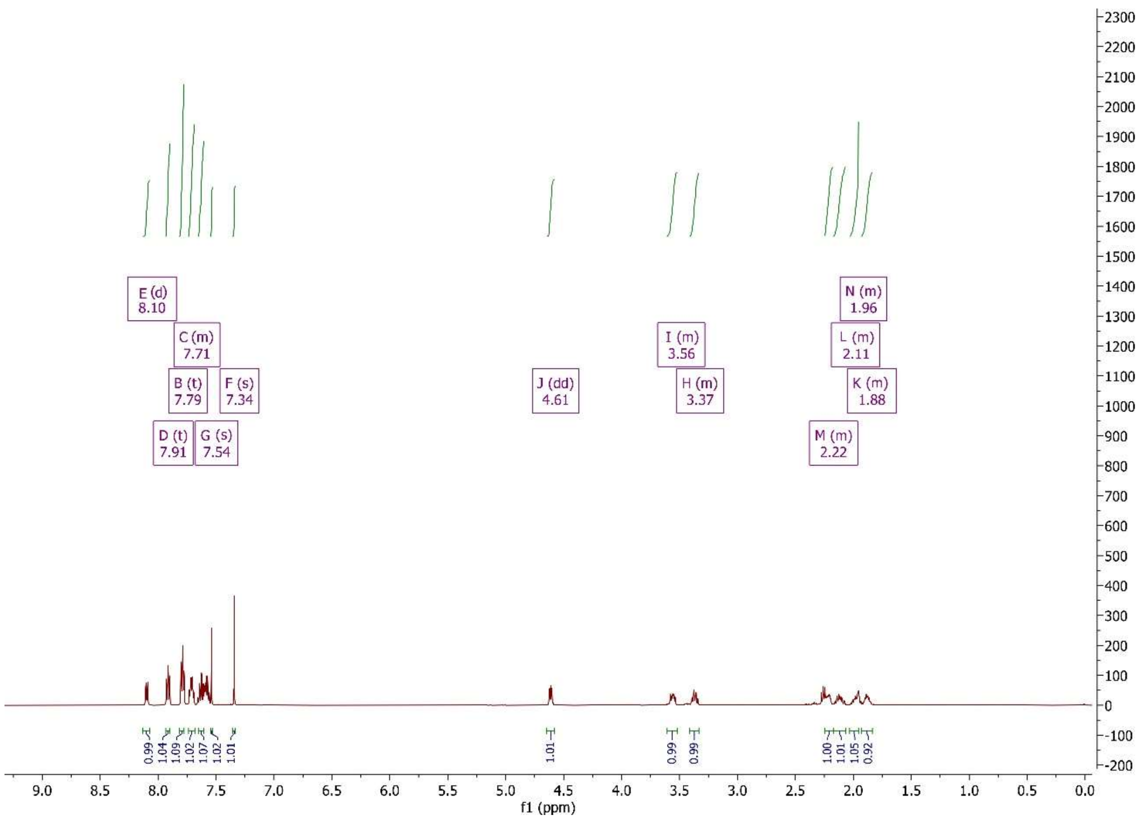

Figure S5: ^{1}H NMR spectrum of compound **2**.

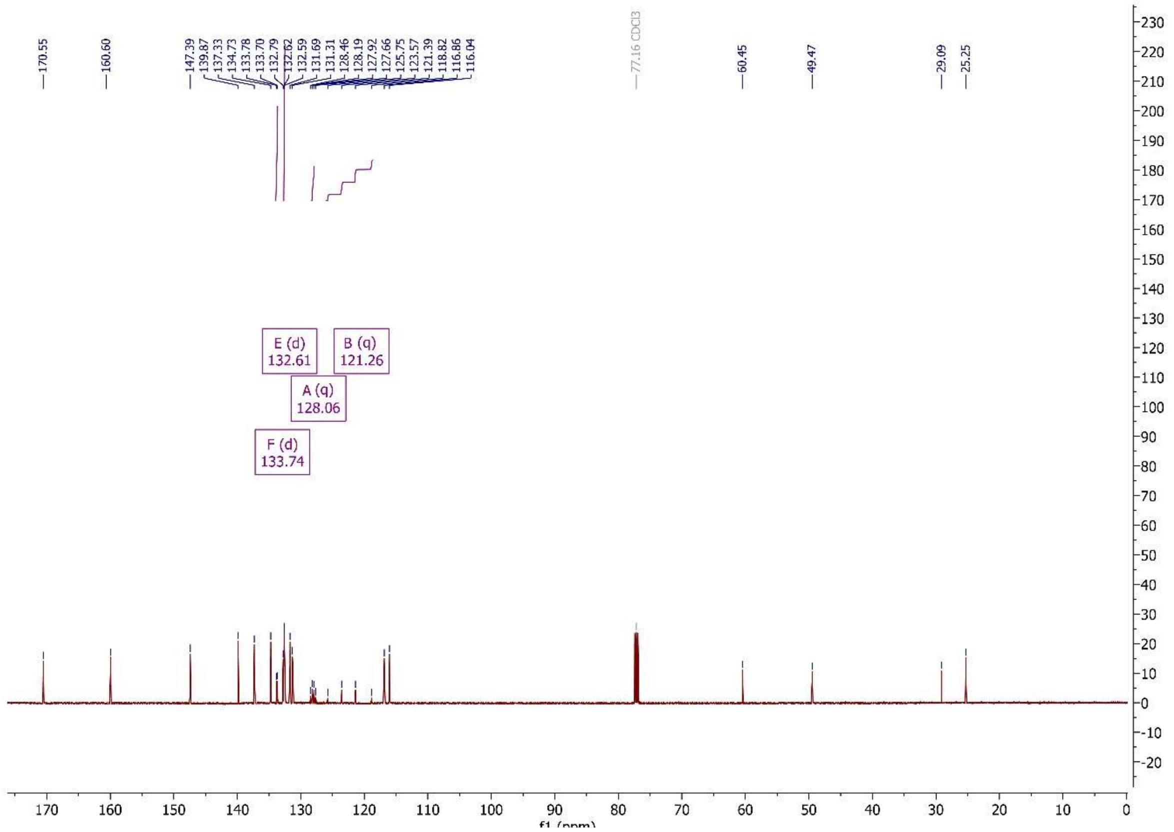

Figure S6: ^{13}C NMR spectrum of compound **2**.

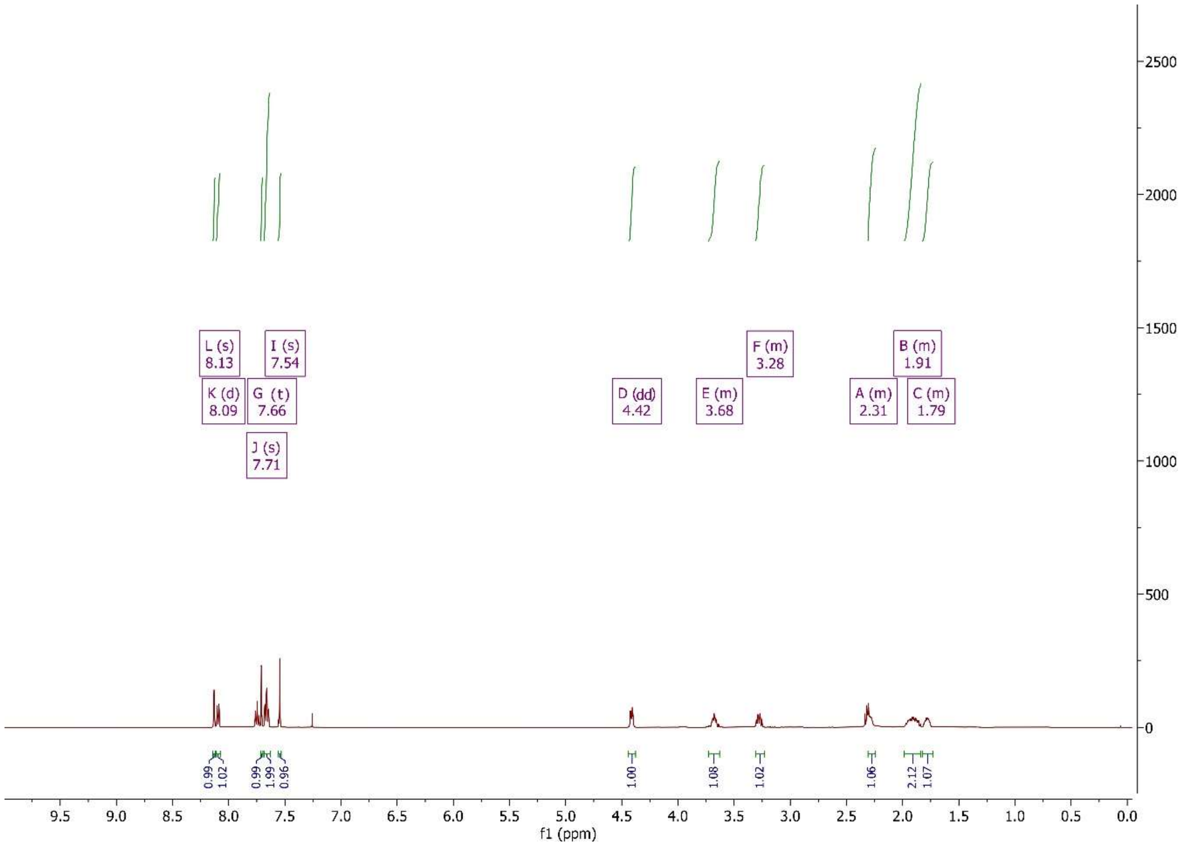

Figure S7: ^{1}H NMR spectrum of compound **3**.

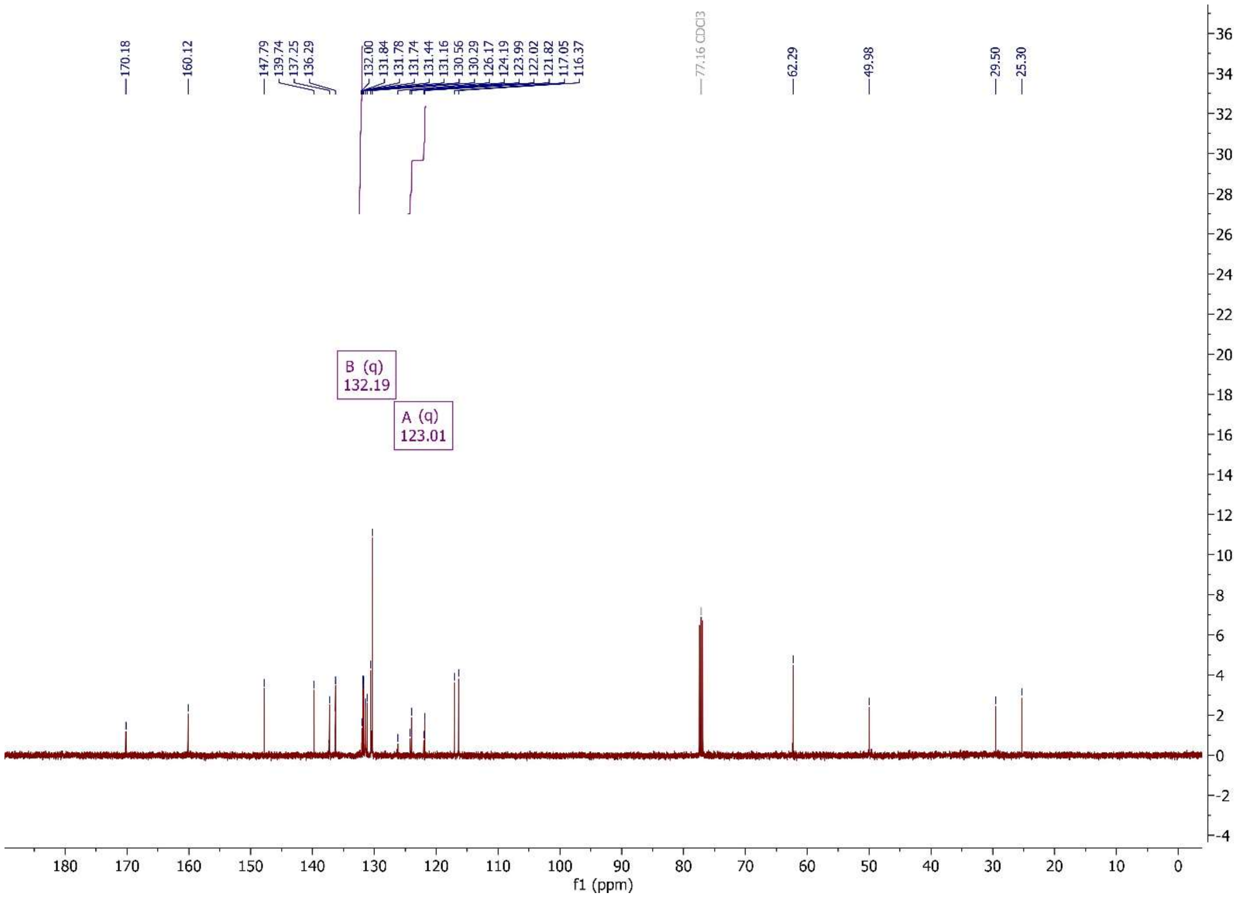

Figure S8: ^{13}C NMR spectrum of compound **3**.

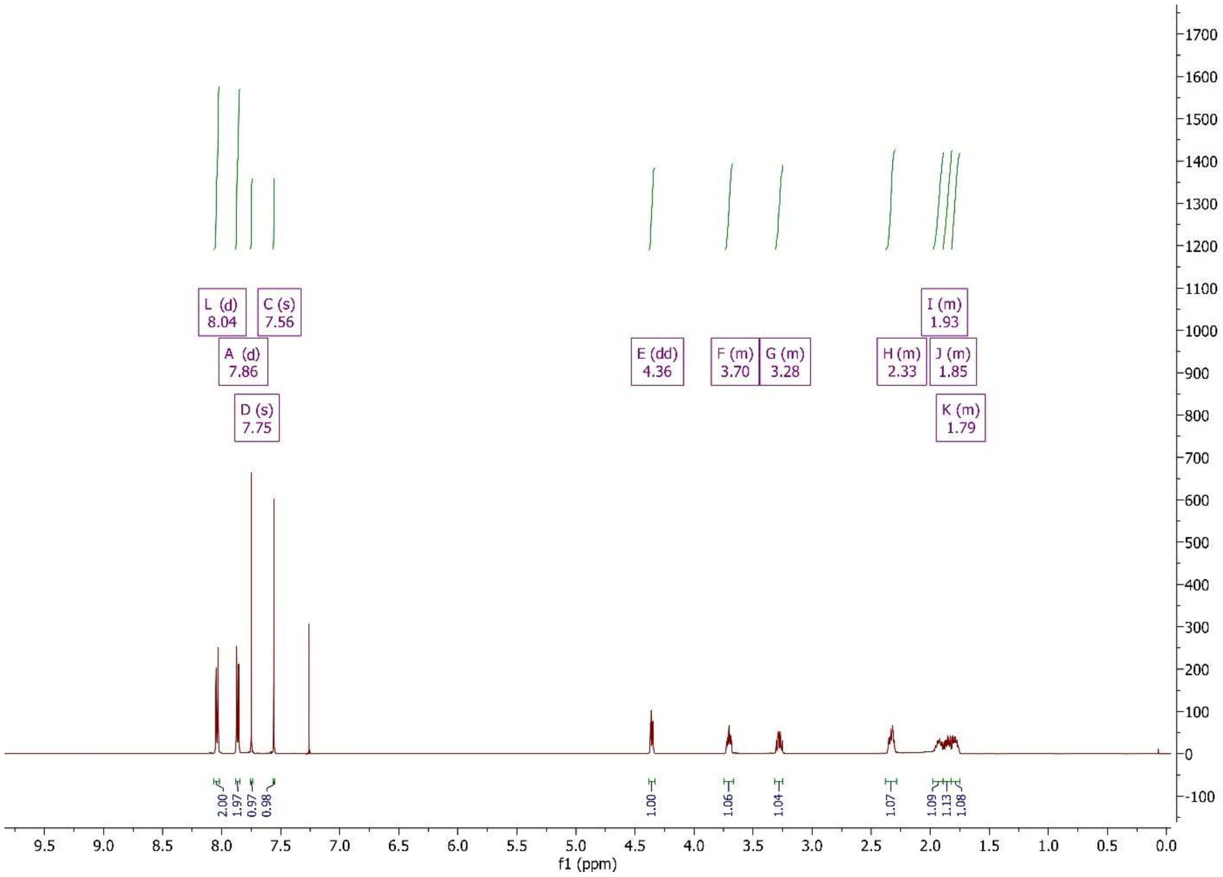

Figure S9: ^{1}H NMR spectrum of compound **4**.

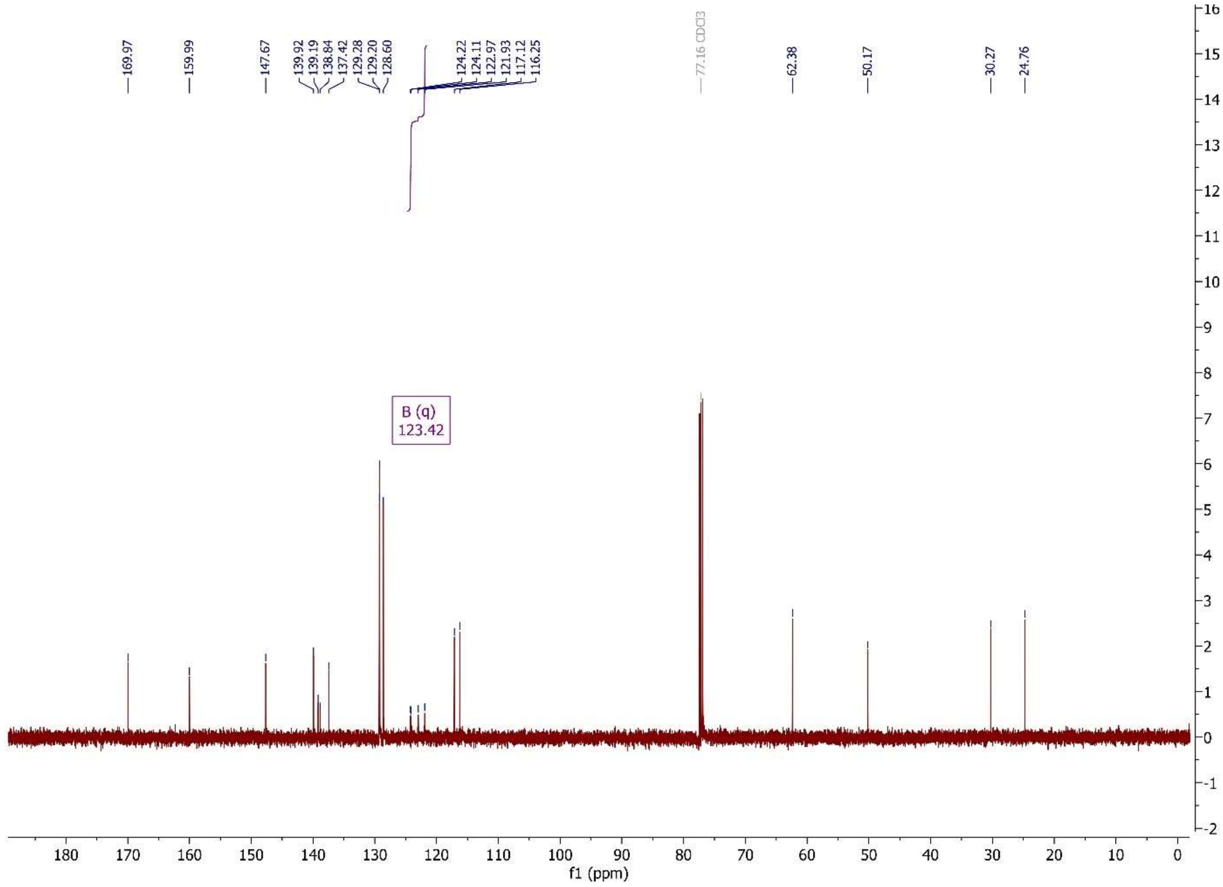

Figure S10: ^{13}C NMR spectrum of compound **4**.

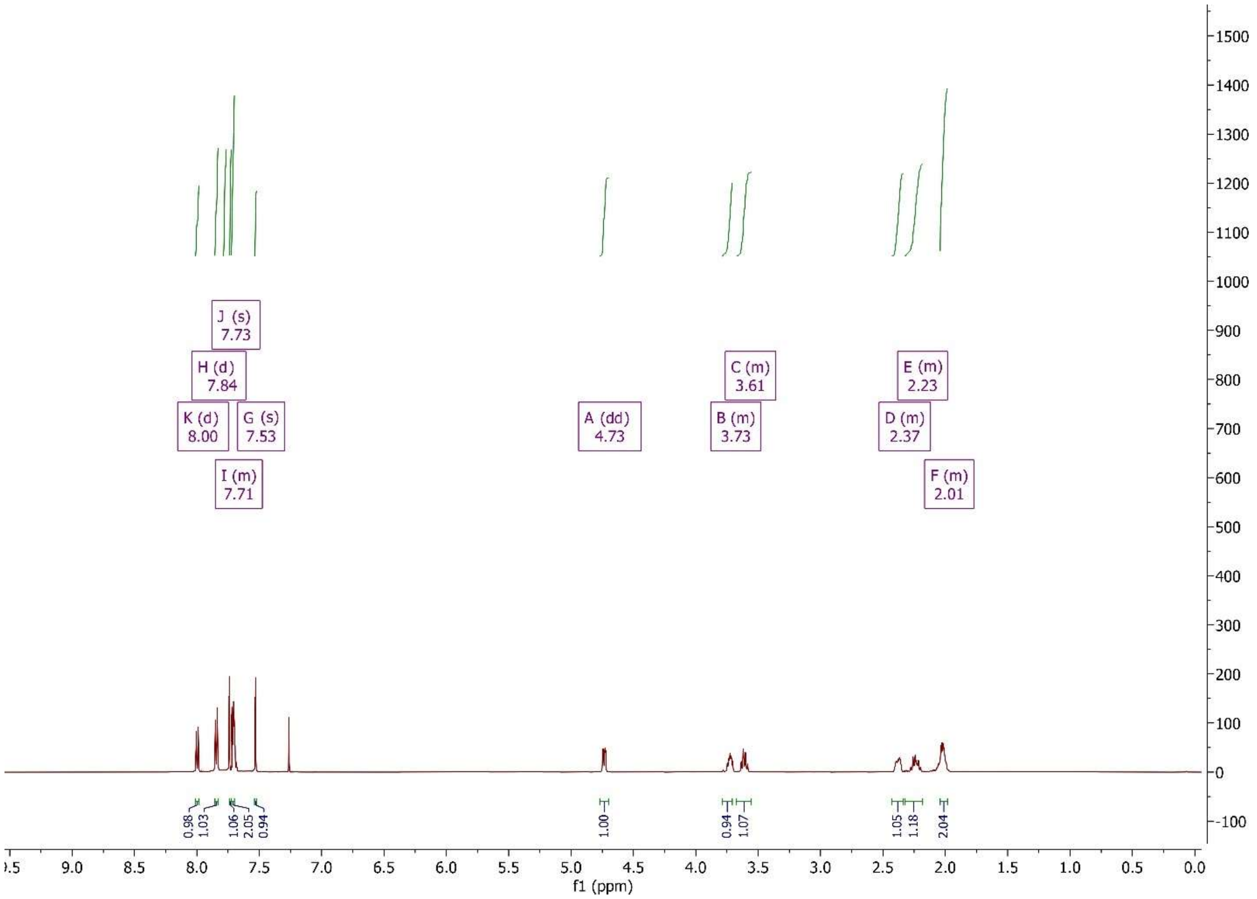

Figure S11: ^{1}H NMR spectrum of compound **5**.

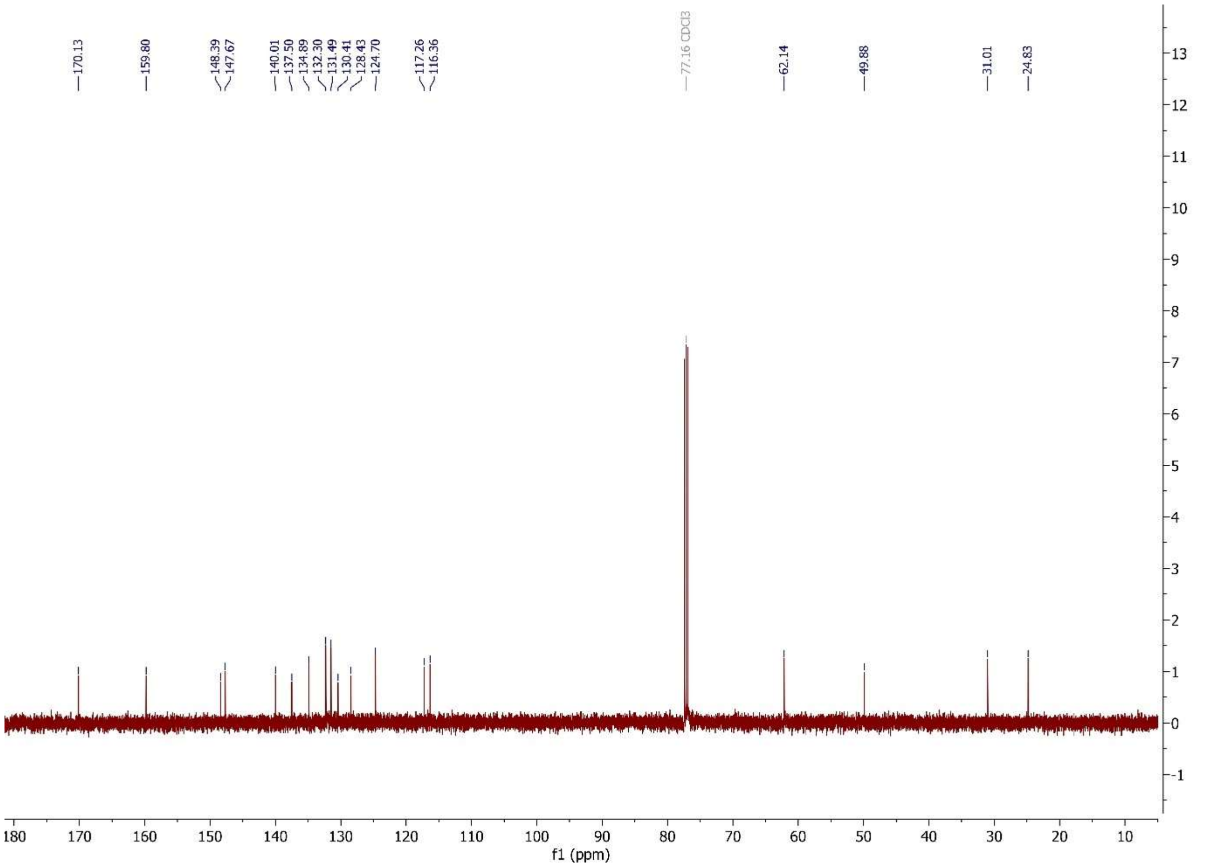

Figure S12: ^{13}C NMR spectrum of compound **5**.

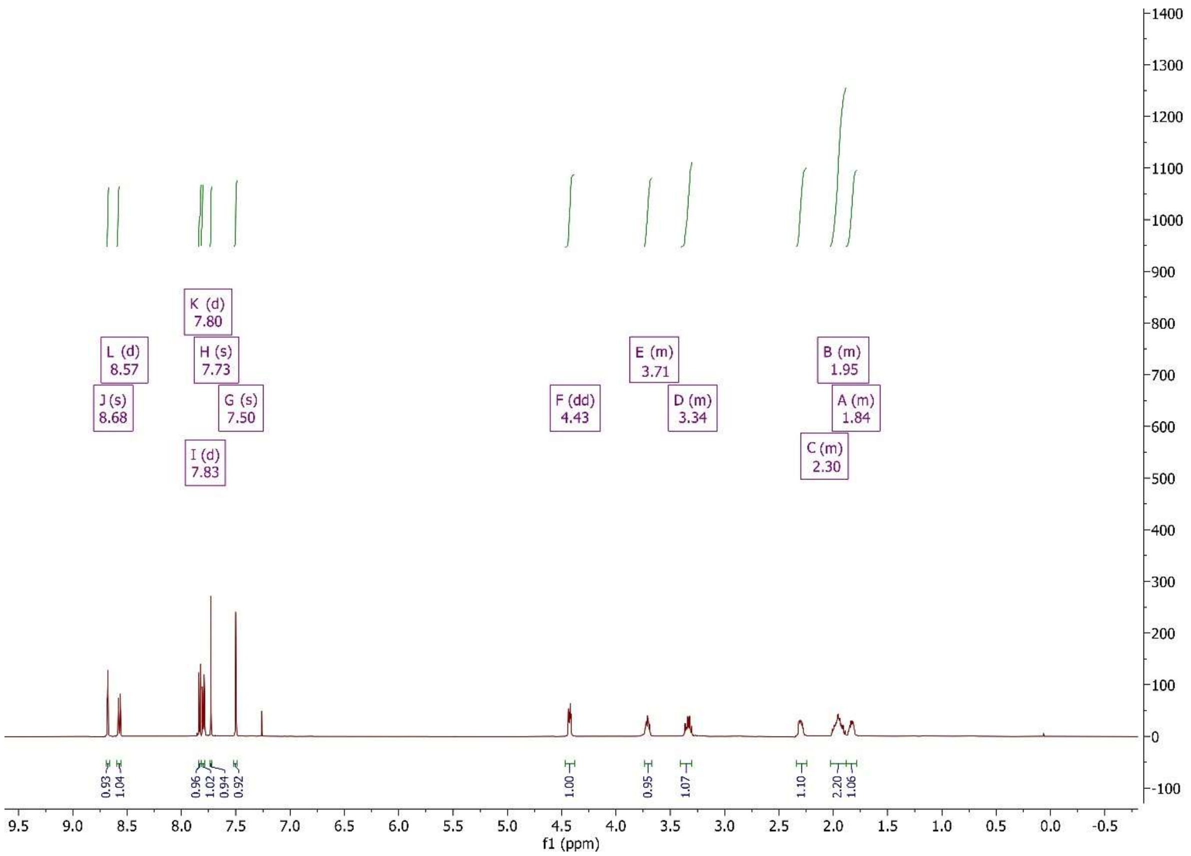

Figure S13: ^{1}H NMR spectrum of compound **6**.

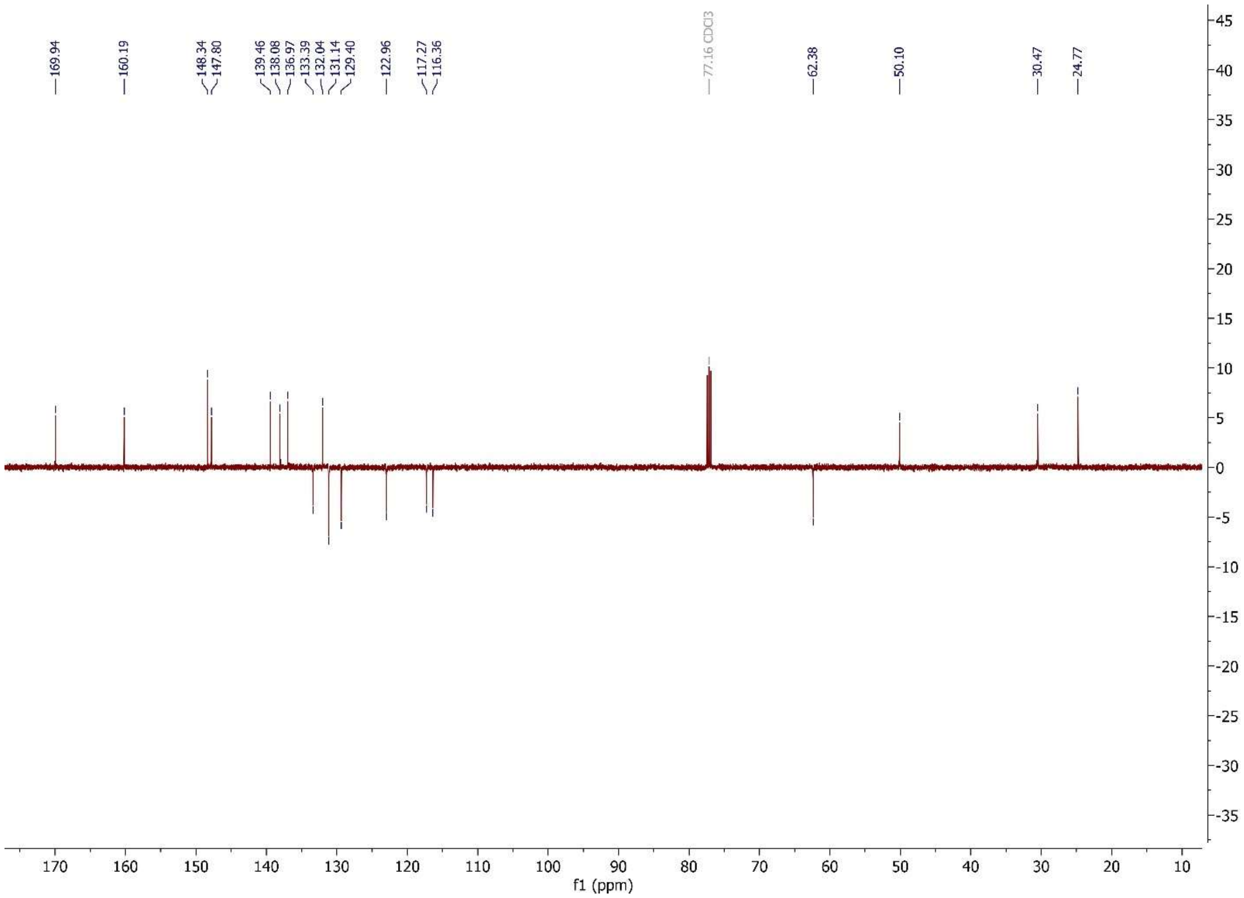

Figure S14: ^{13}C NMR spectrum of compound **6**.

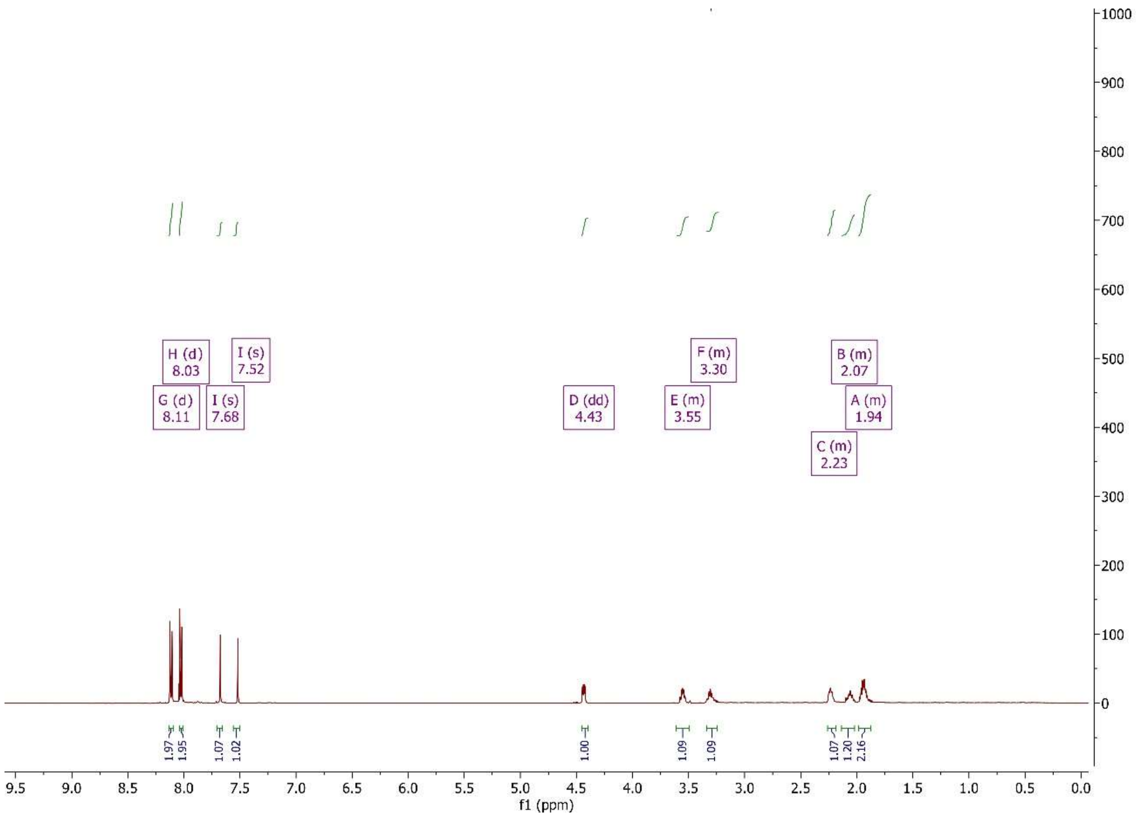

Figure S15: ^{1}H NMR spectrum of compound **7**.

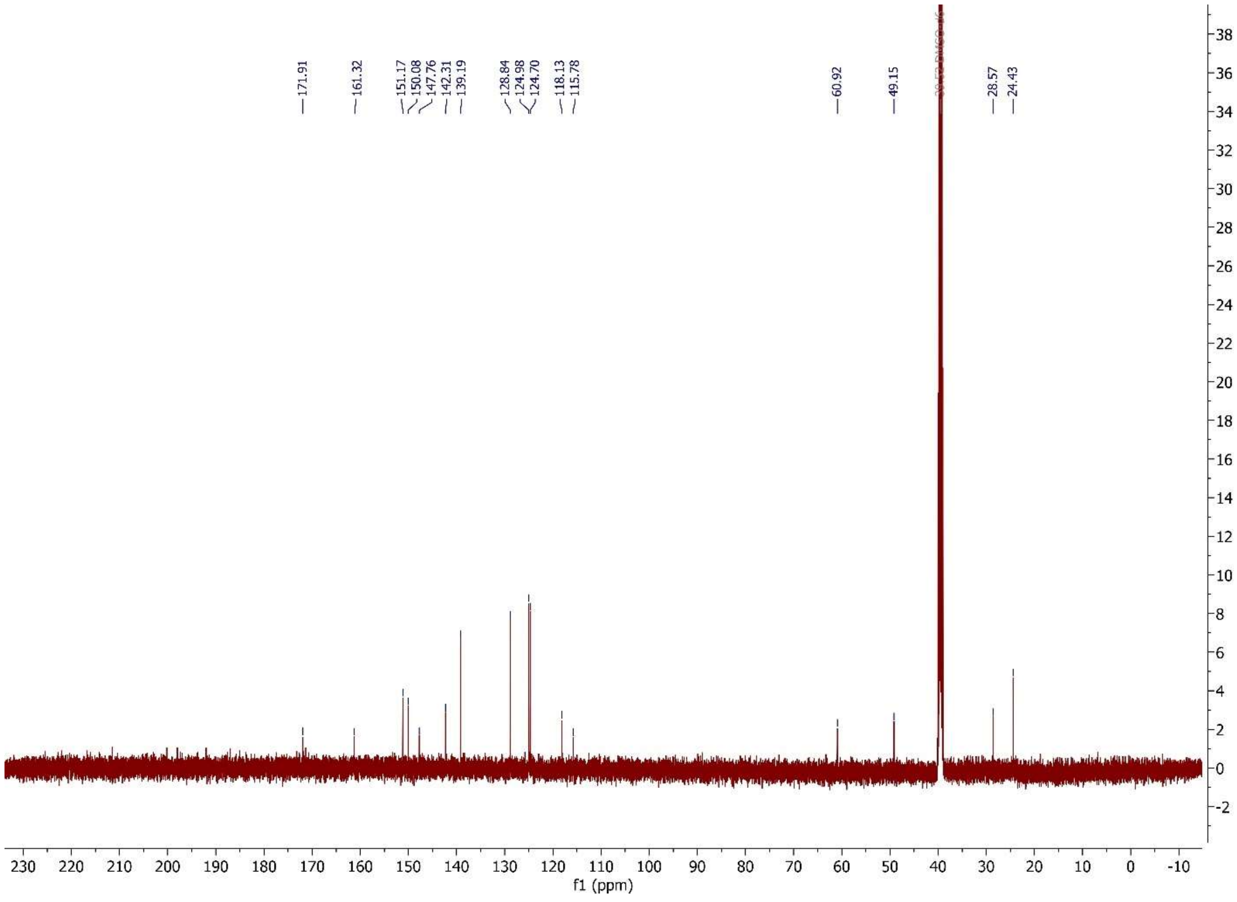

Figure S16: ^{13}C NMR spectrum of compound **7**.

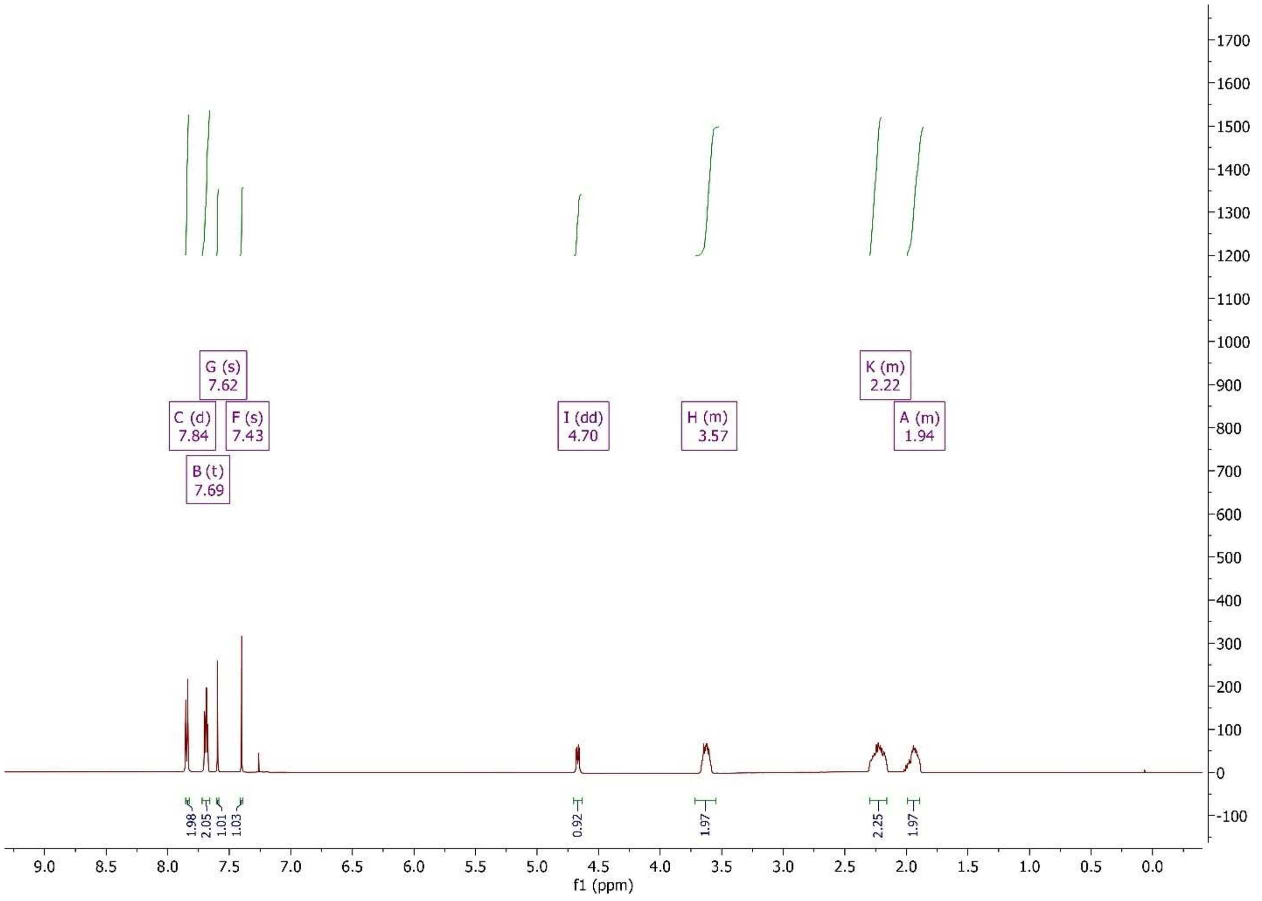

Figure S17: ^{1}H NMR spectrum of compound **8**.

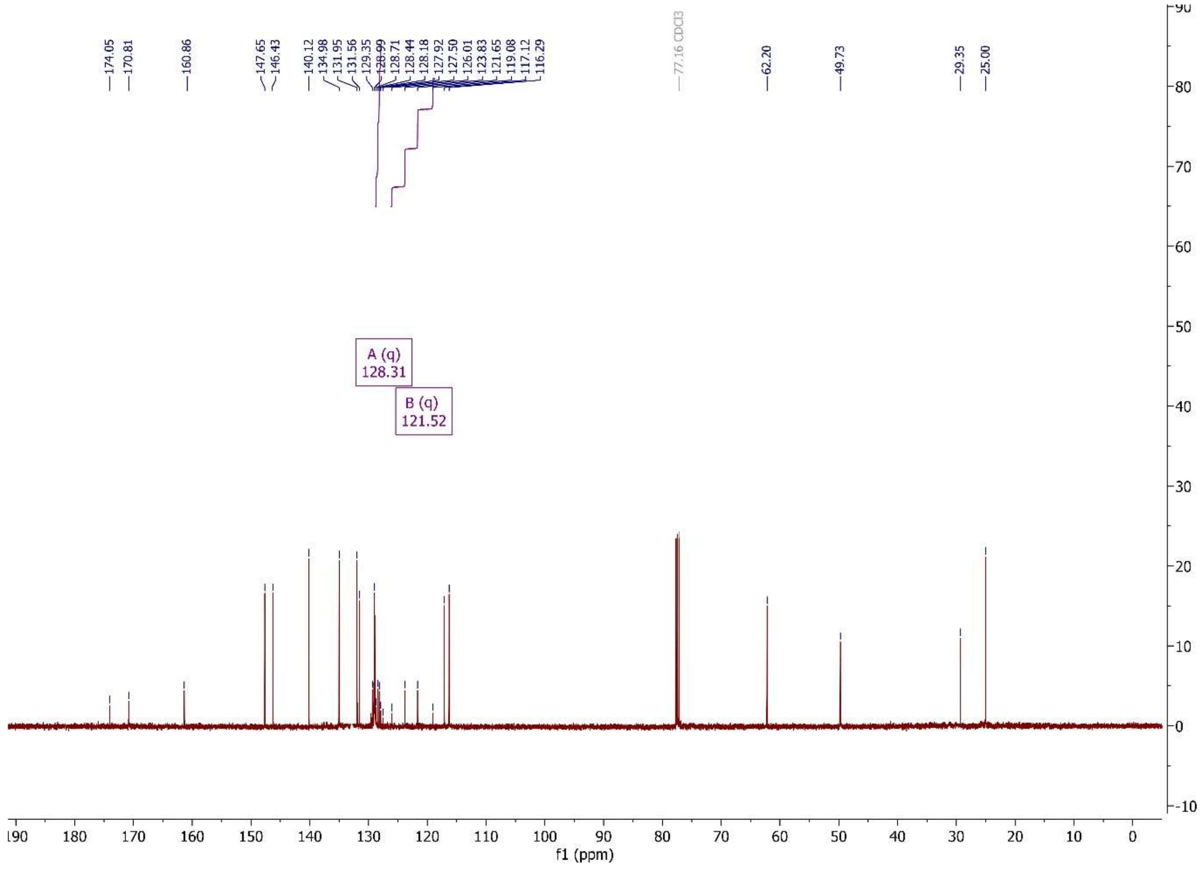

Figure S18: ^{13}C NMR spectrum of compound **8**.

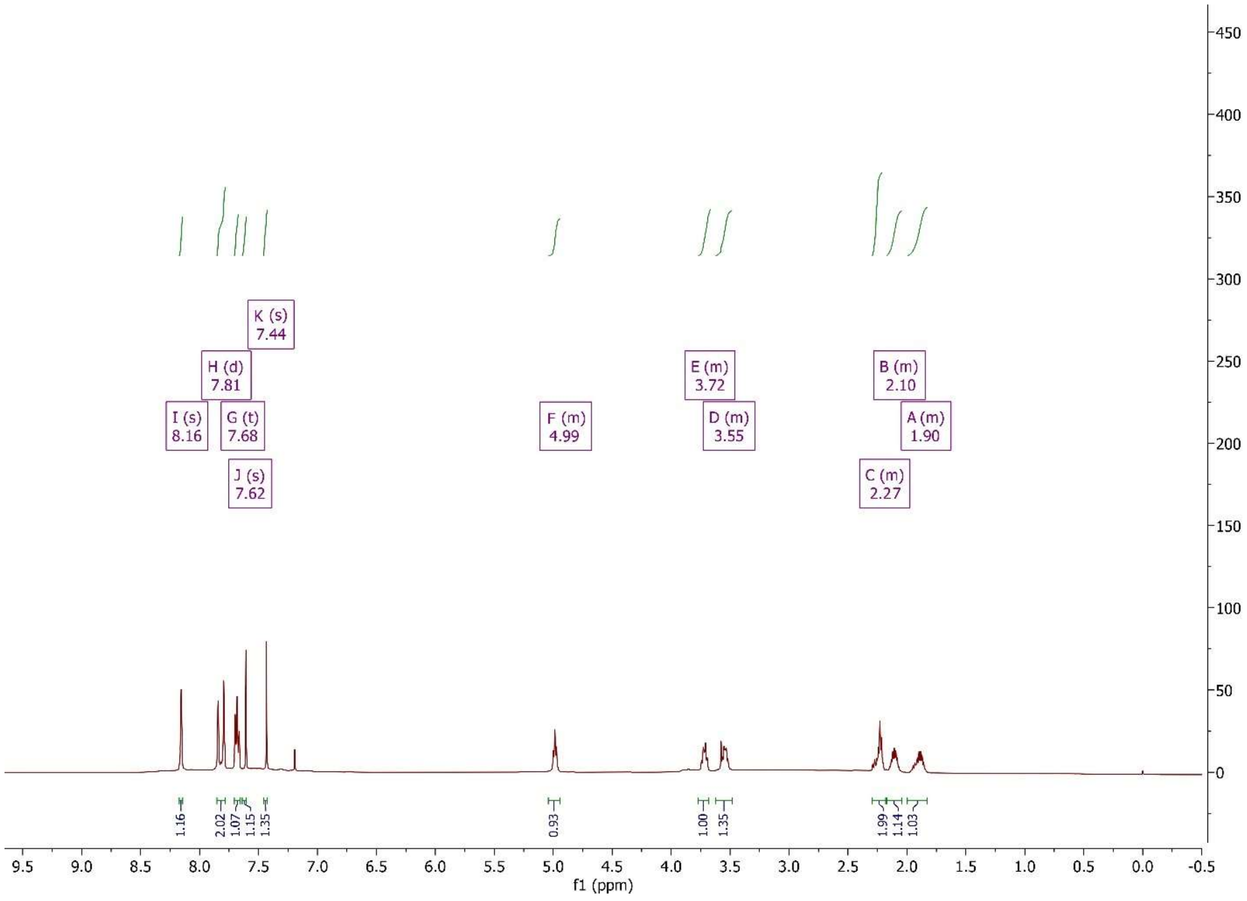

Figure S19: ^{1}H NMR spectrum of compound **9**.

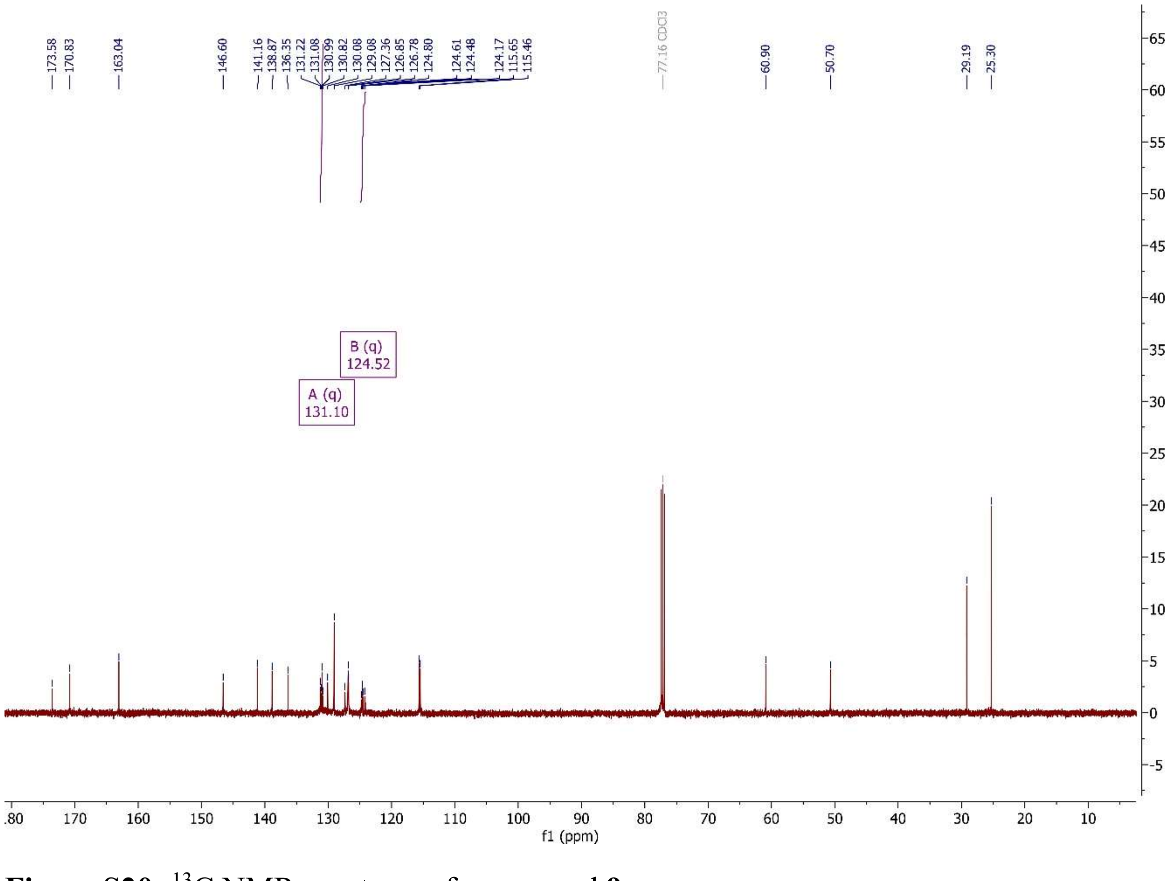

Figure S20: ^{13}C NMR spectrum of compound **9**.

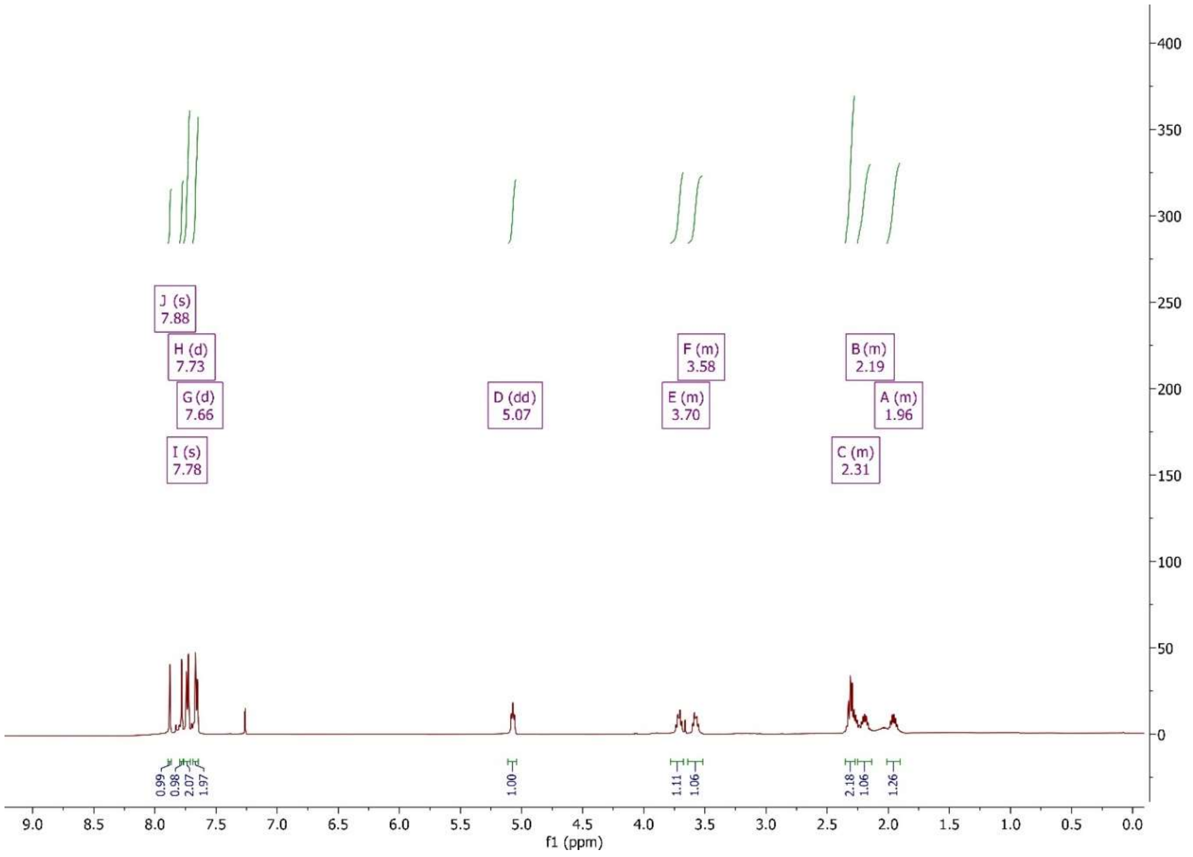

Figure S21: ^{1}H NMR spectrum of compound **10**.

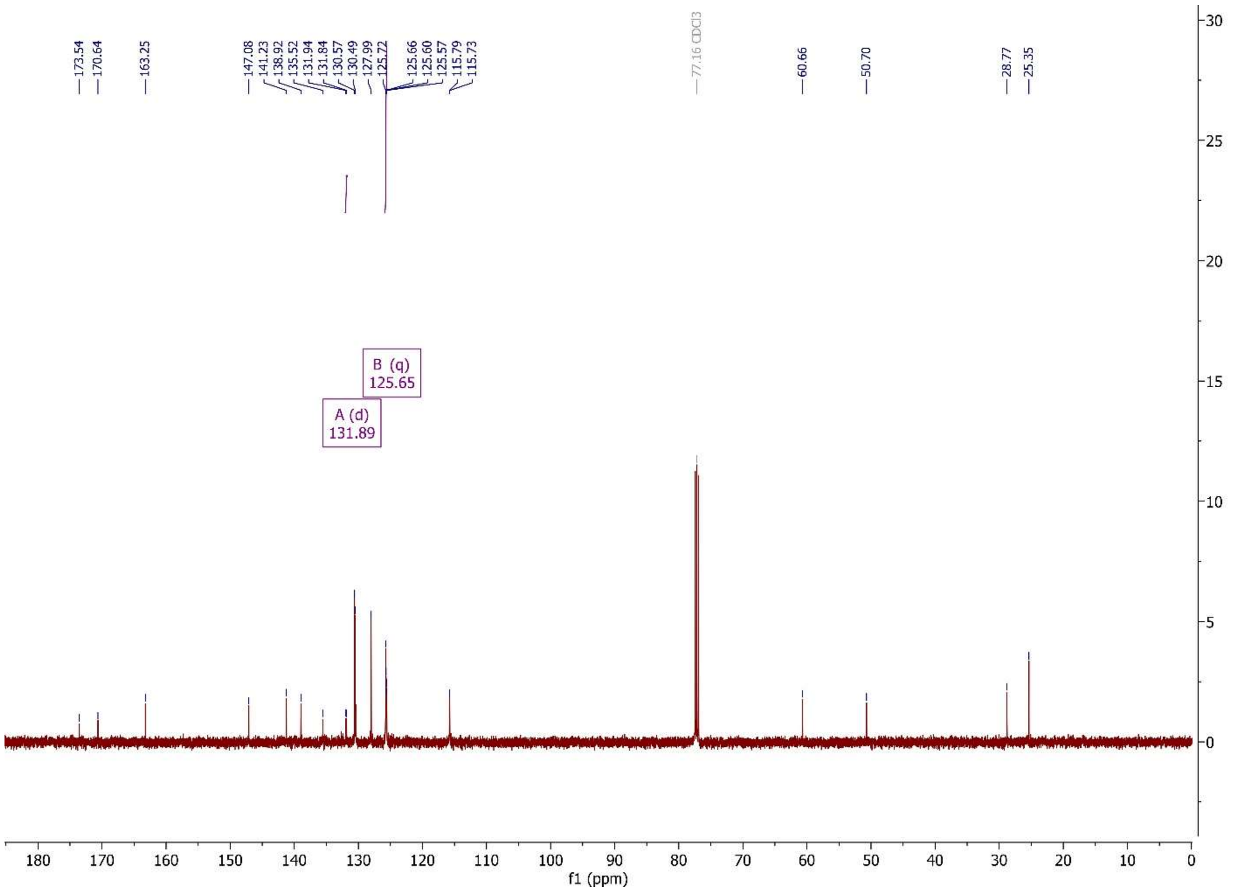

Figure S22: ^{13}C NMR spectrum of compound **10.**

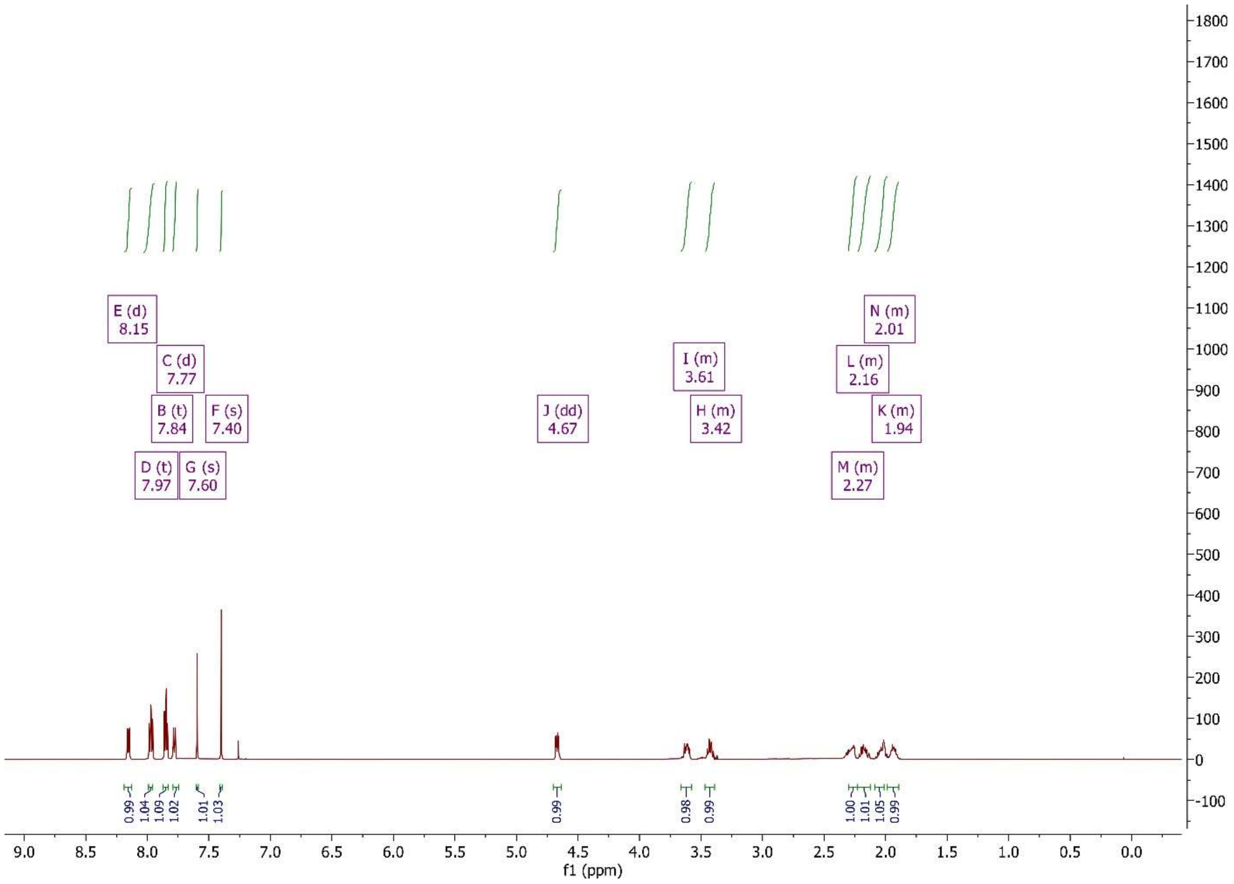

Figure S23: ^{1}H NMR spectrum of compound **11**.

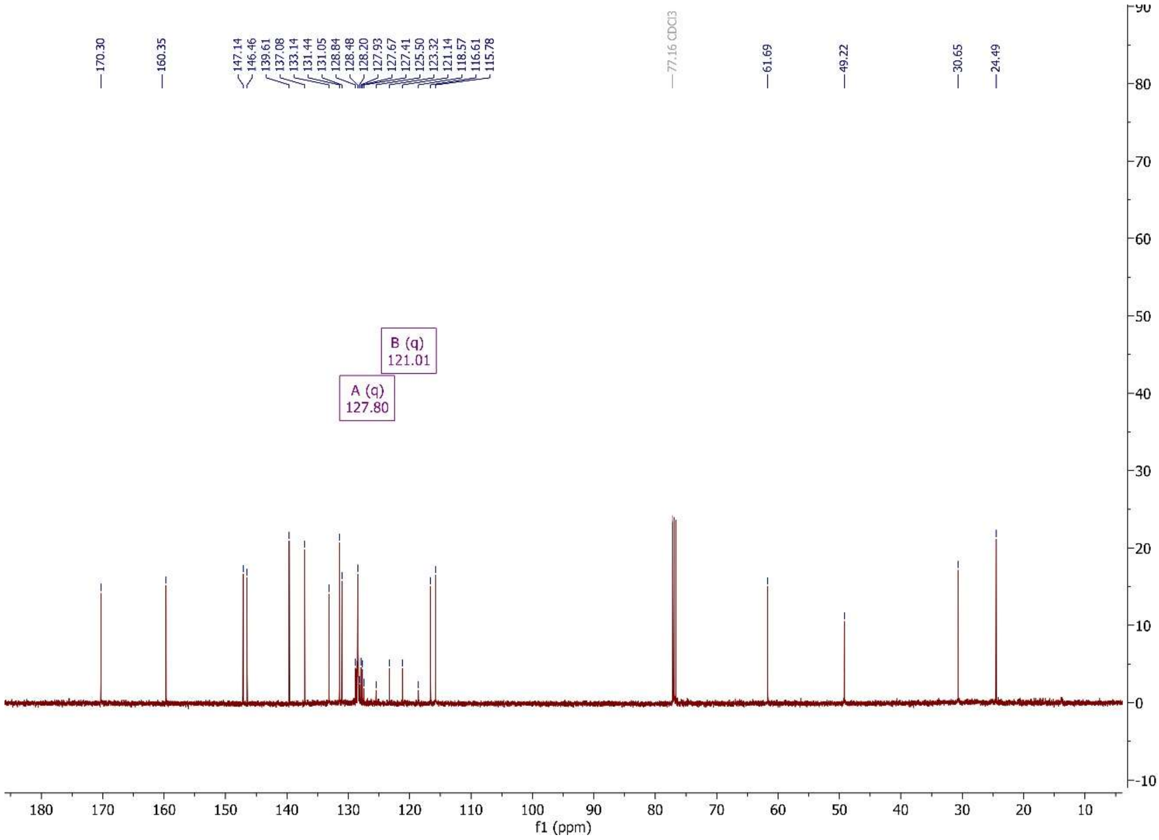

Figure S24: ^{13}C NMR spectrum of compound **11.**

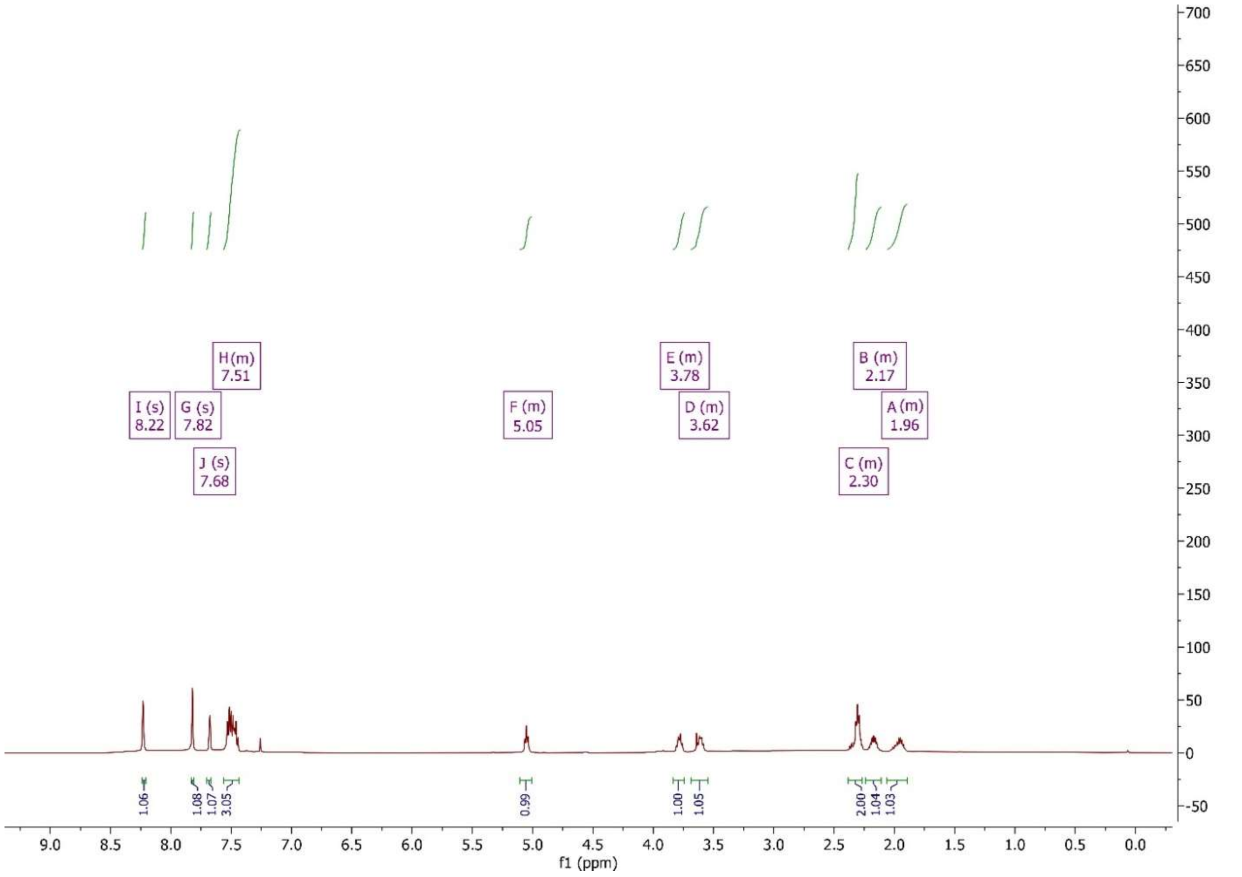

Figure S25: ^{1}H NMR spectrum of compound **12**.

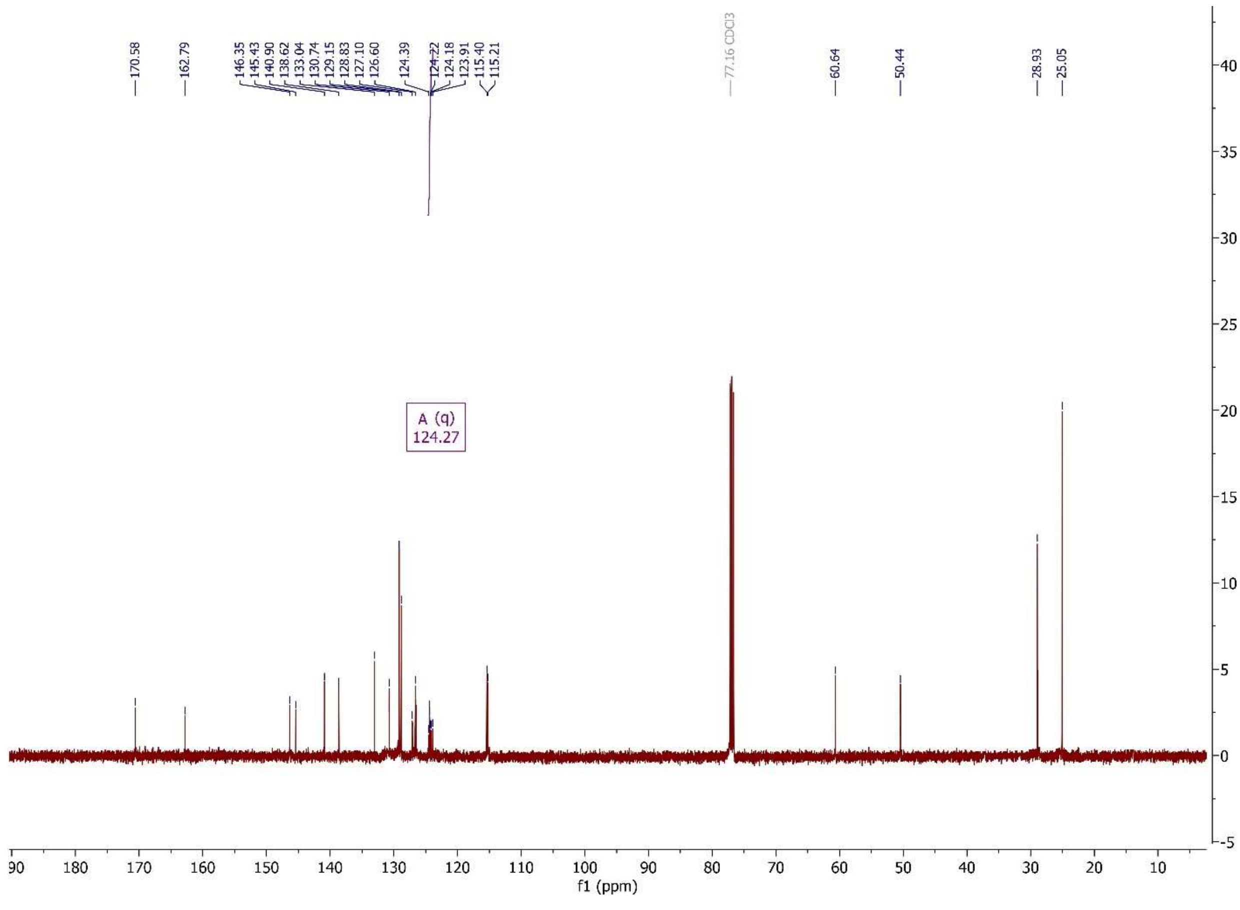

Figure S26: ^{13}C NMR spectrum of compound **12.**

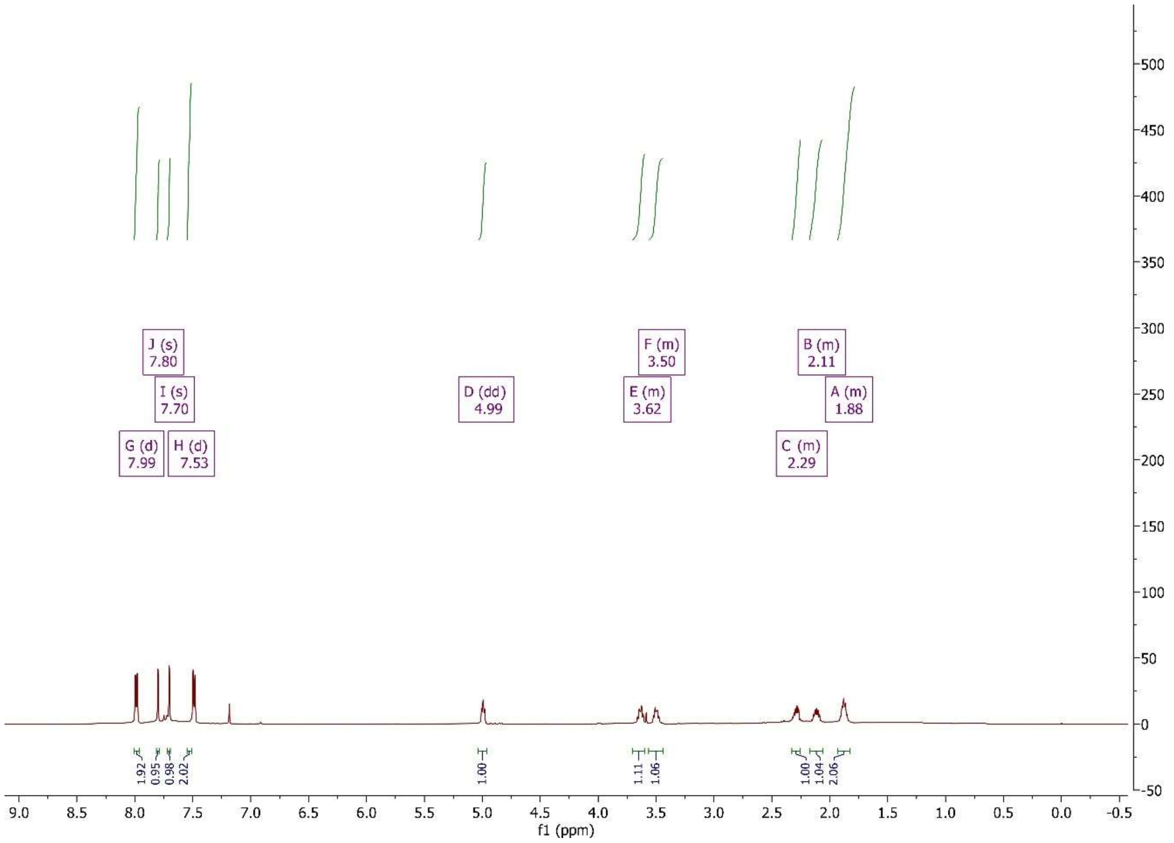

Figure S27: ^{1}H NMR spectrum of compound **13**.

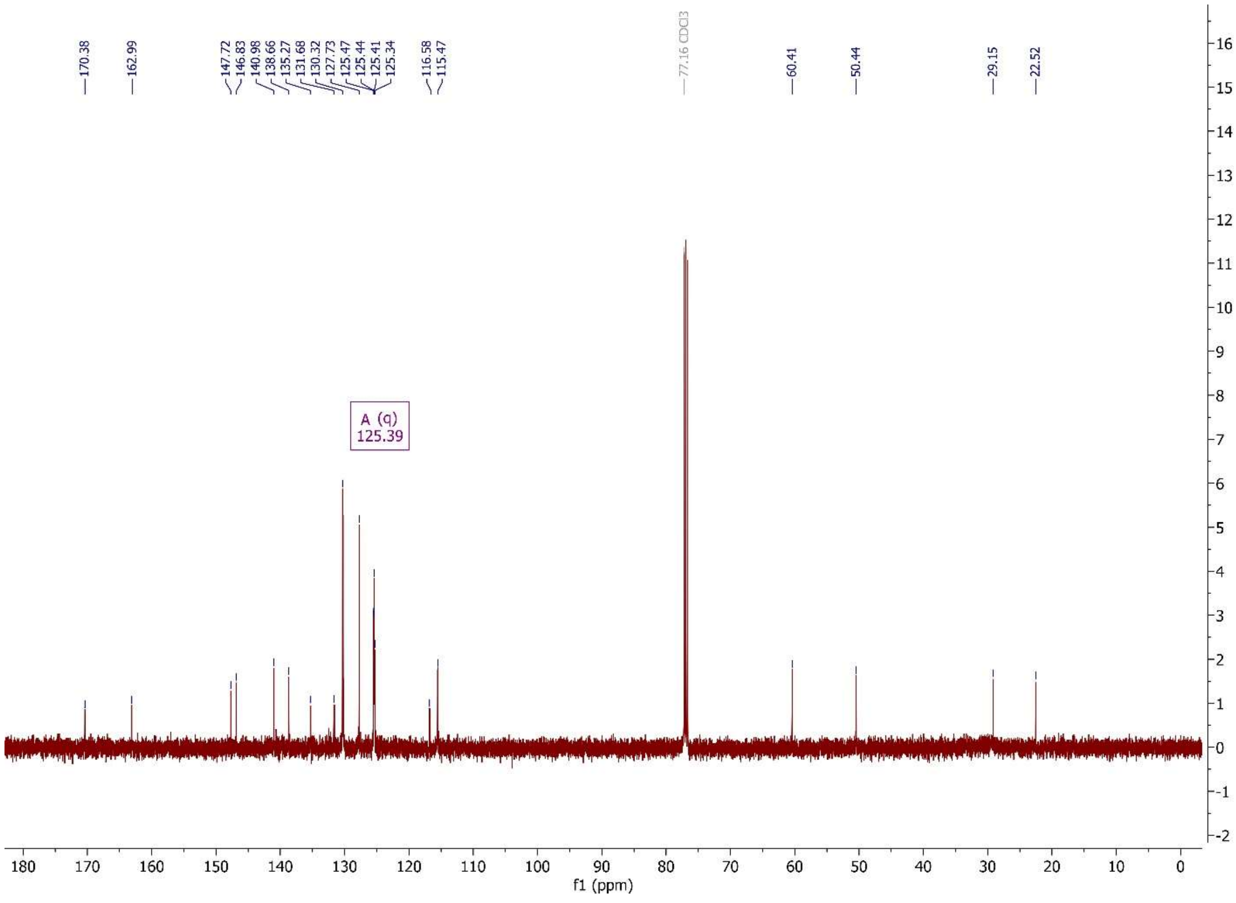

Figure S28: ^{13}C NMR spectrum of compound **13.**

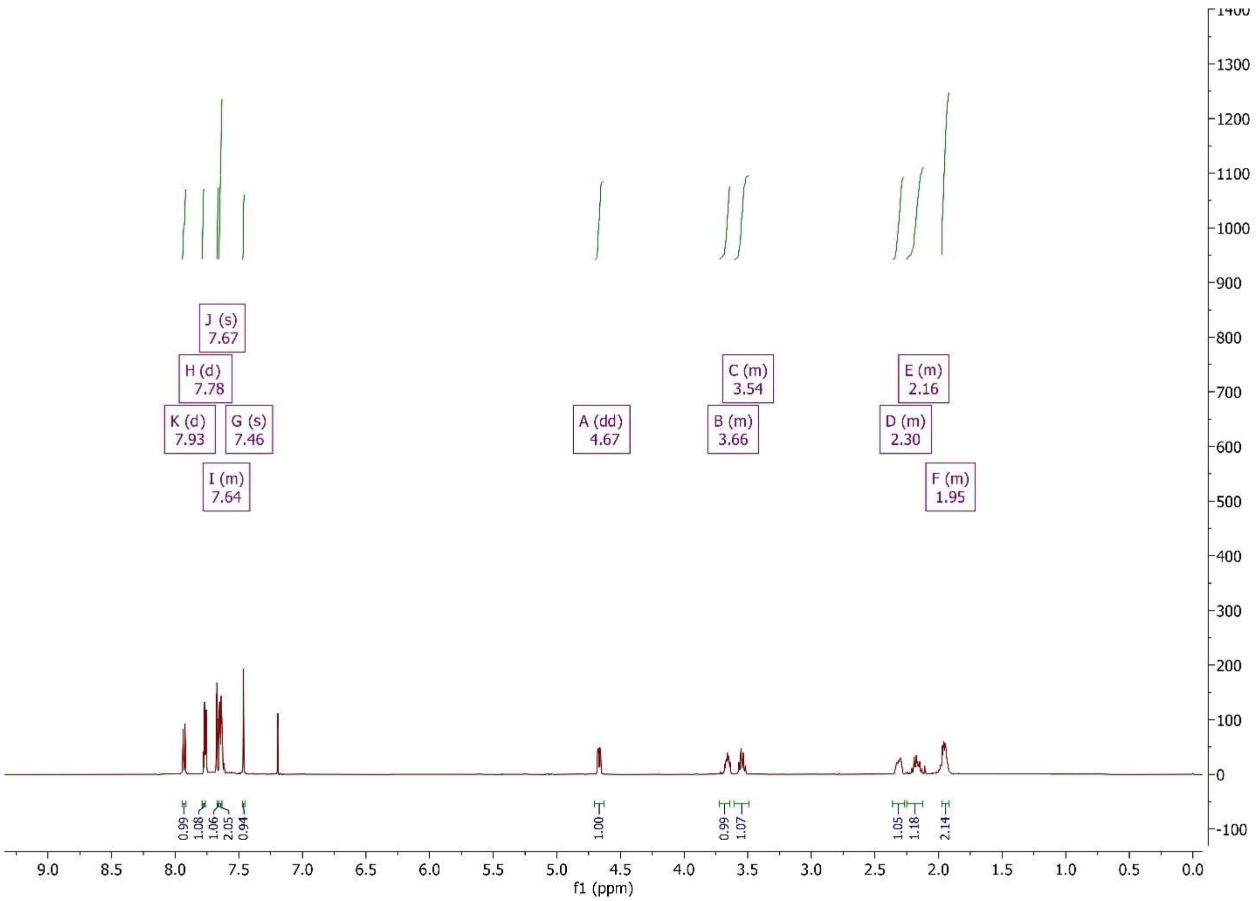

Figure S29: ^{1}H NMR spectrum of compound **14**.

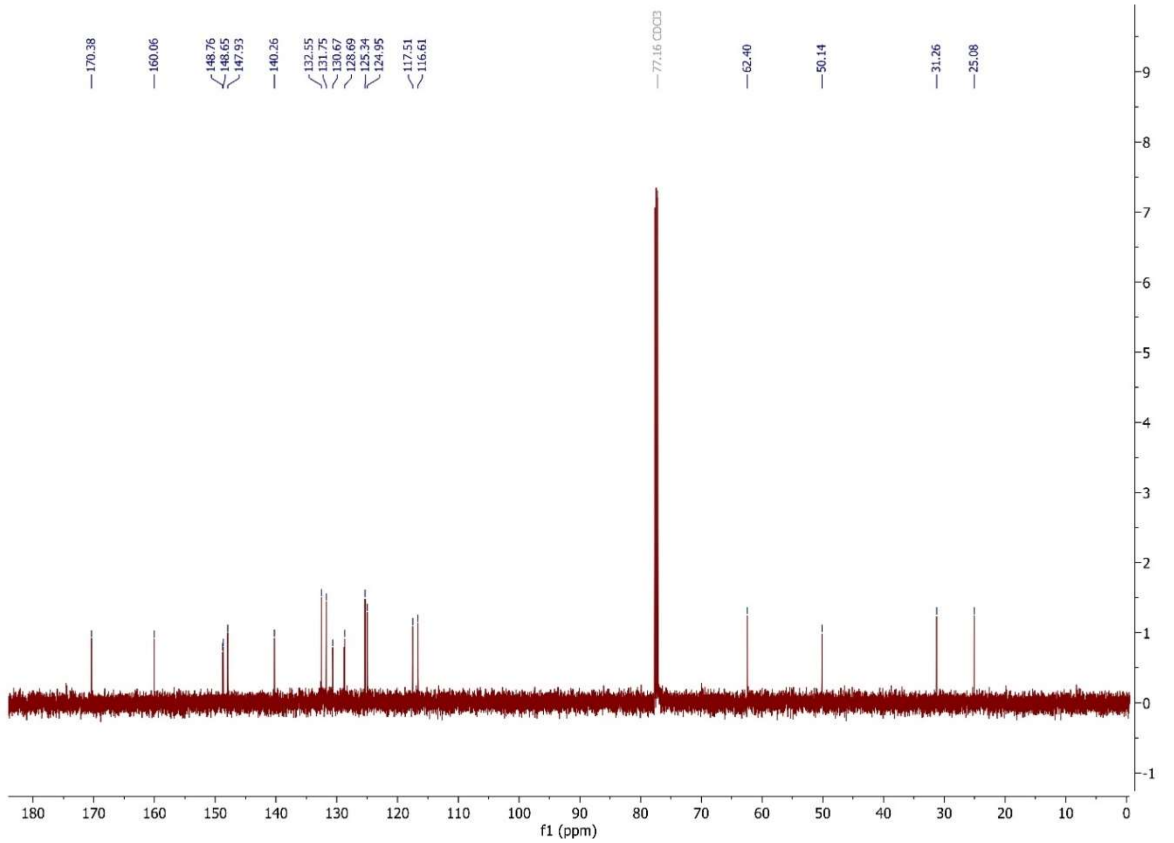

Figure S30: ^{13}C NMR spectrum of compound **14.**

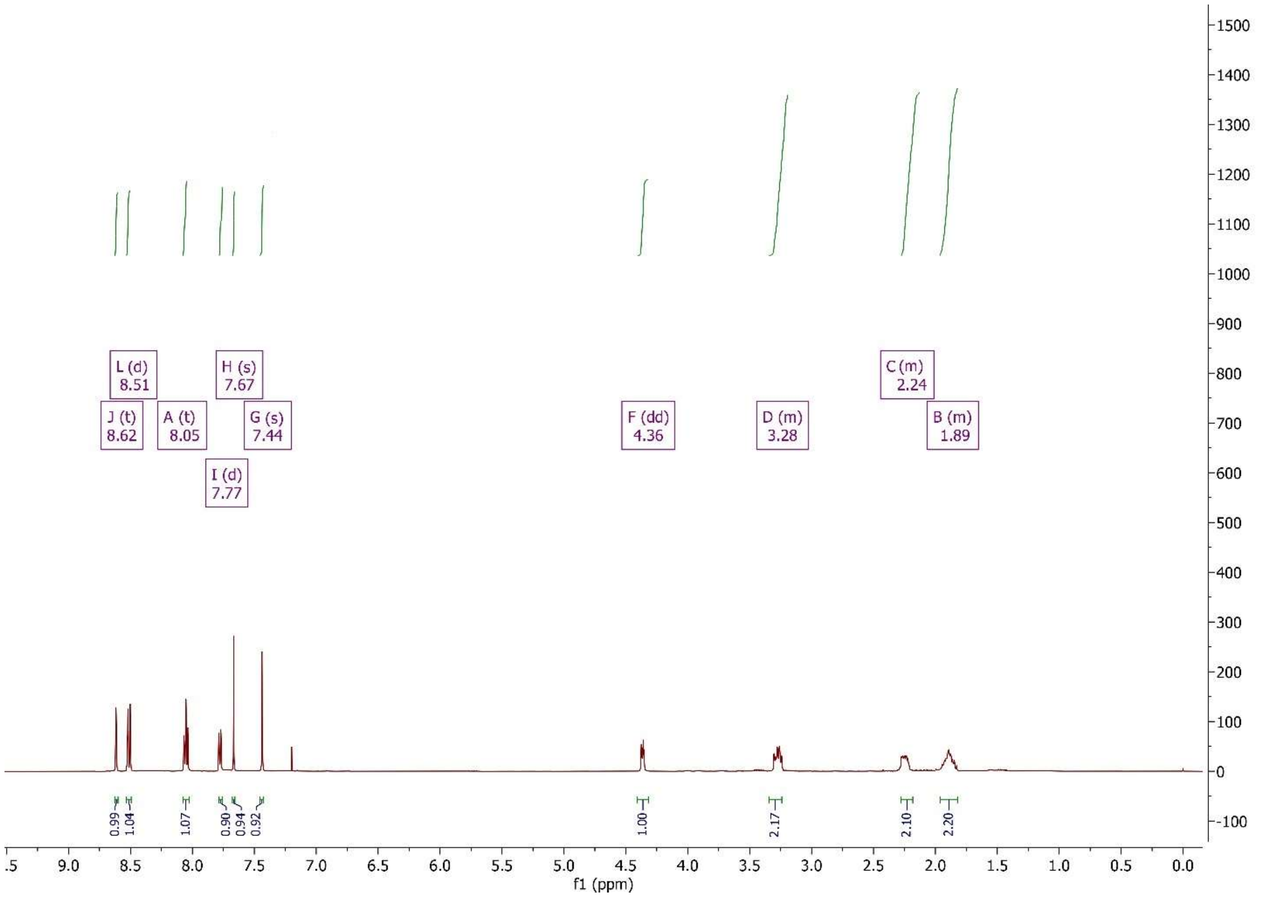

Figure S31: ^{1}H NMR spectrum of compound **15**.

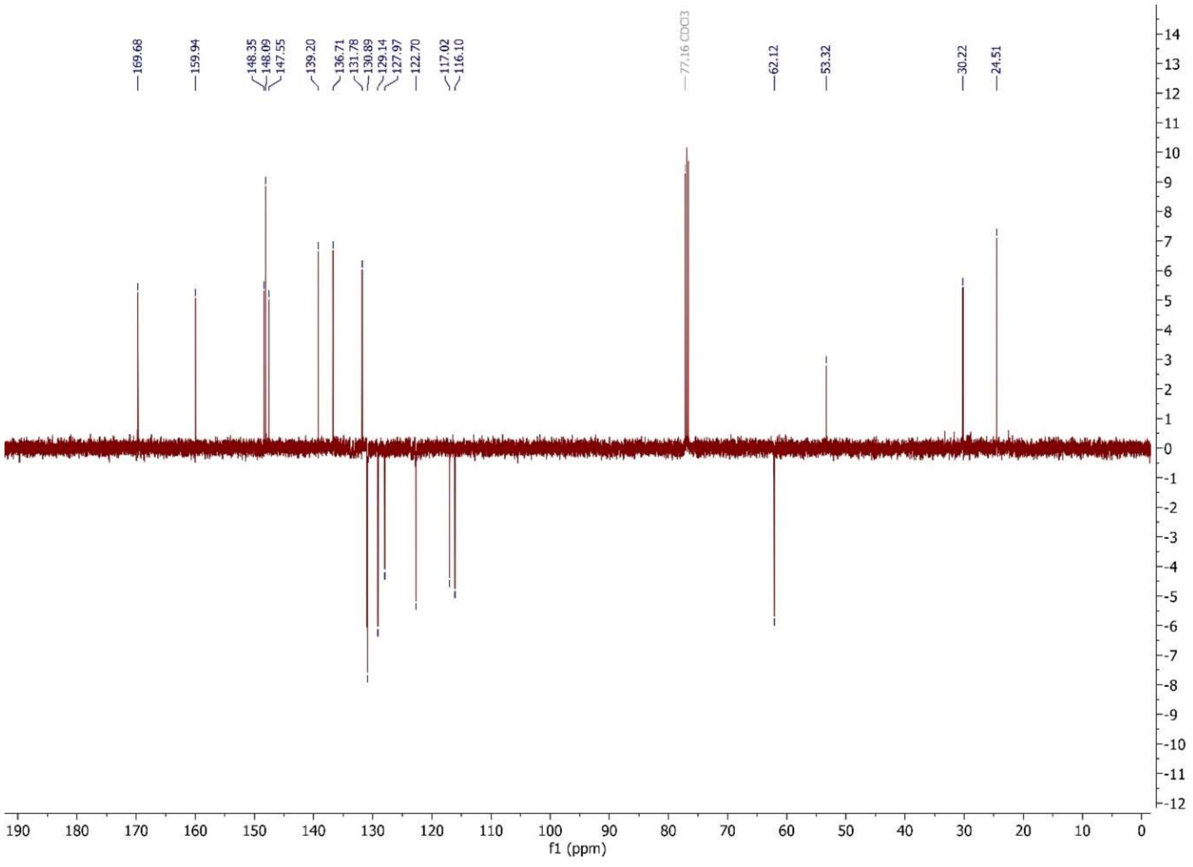

Figure S32: ^{13}C NMR spectrum of compound **15.**

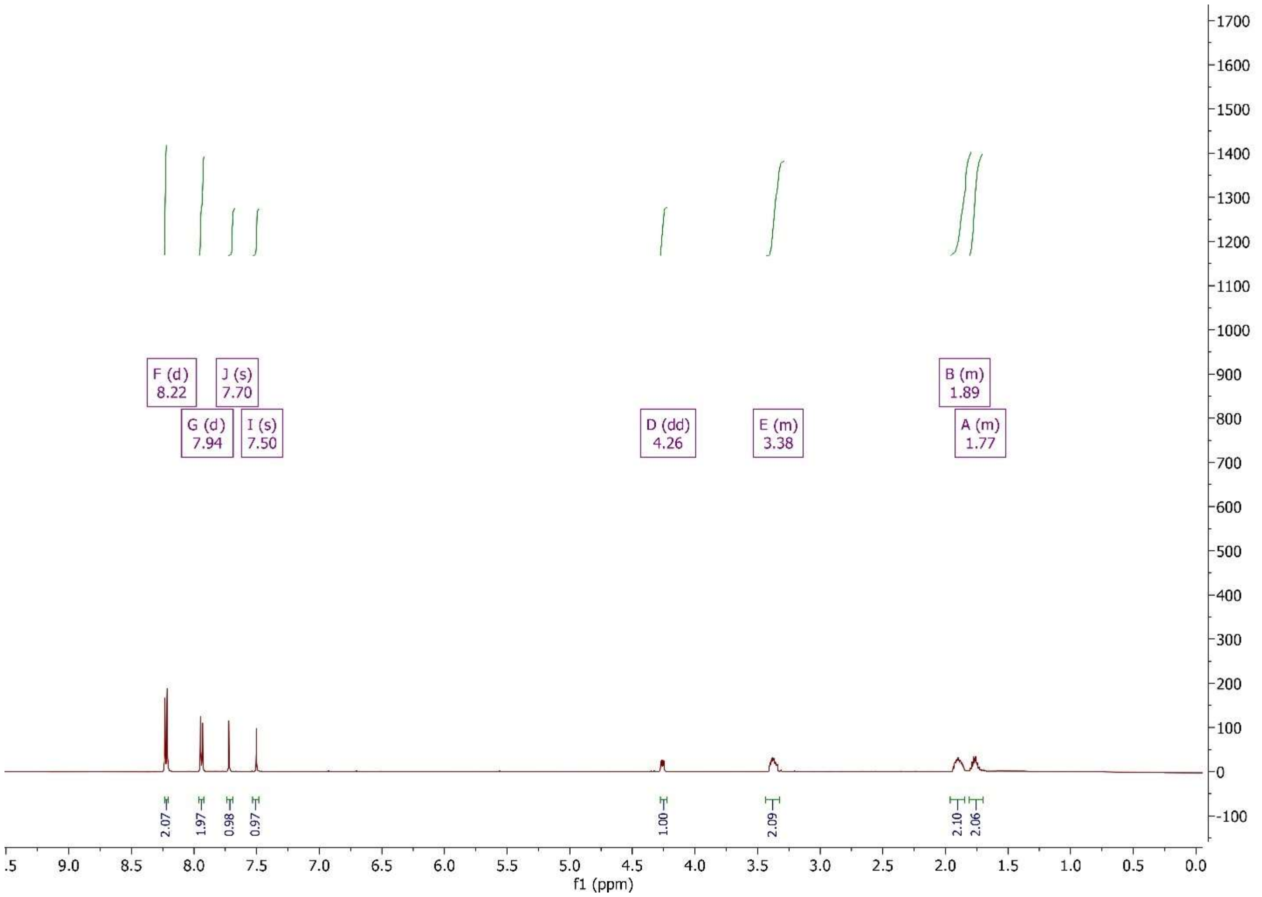

Figure S33: ^{1}H NMR spectrum of compound **16**.

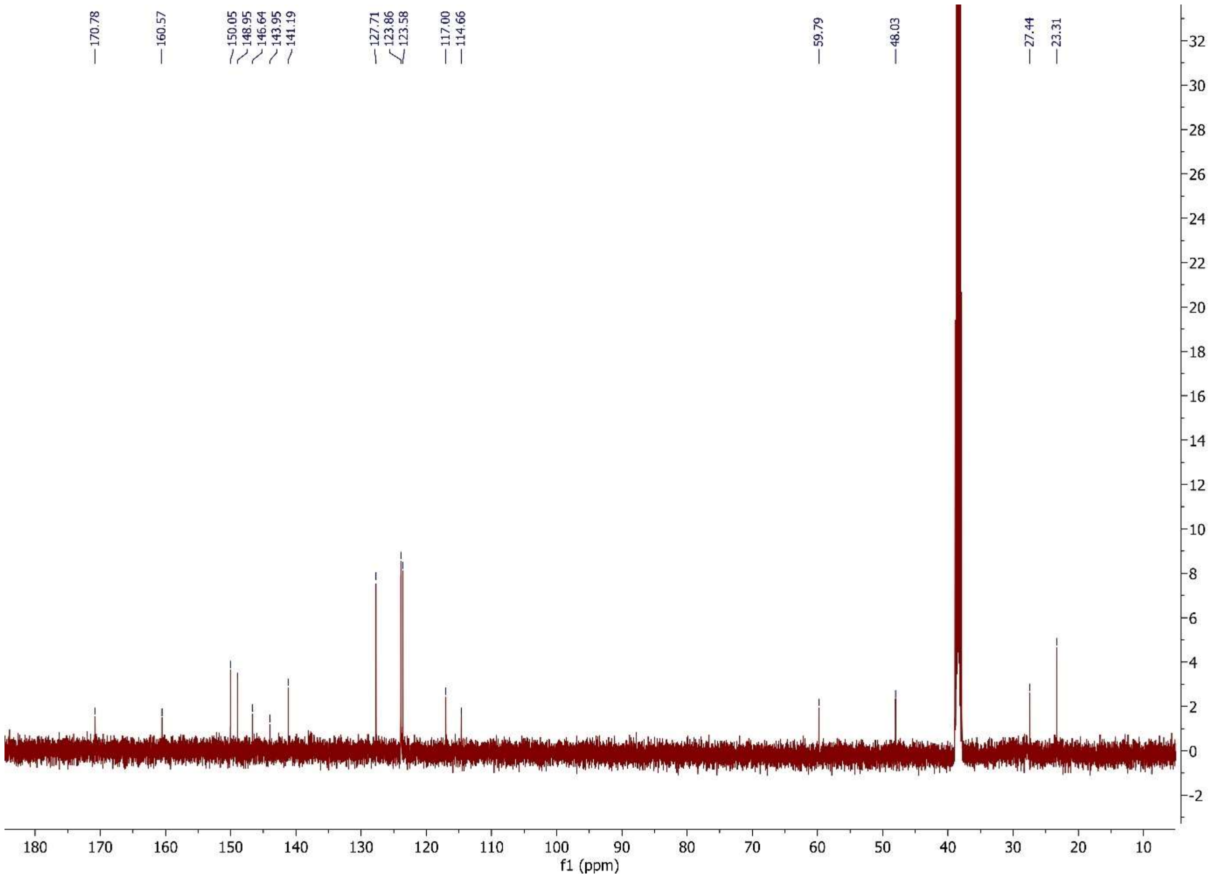

Figure S34: ^{13}C NMR spectrum of compound **16.**

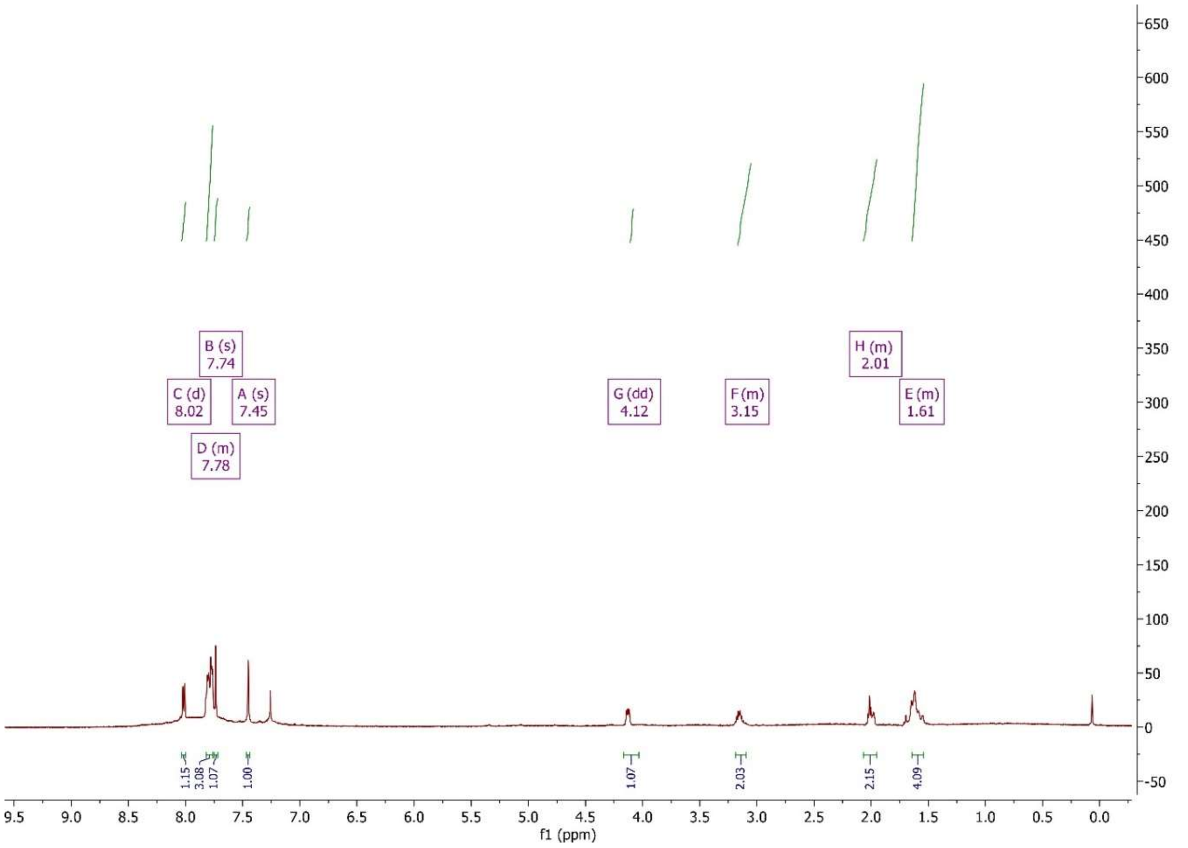

Figure S35: ^{1}H NMR spectrum of compound **17**.

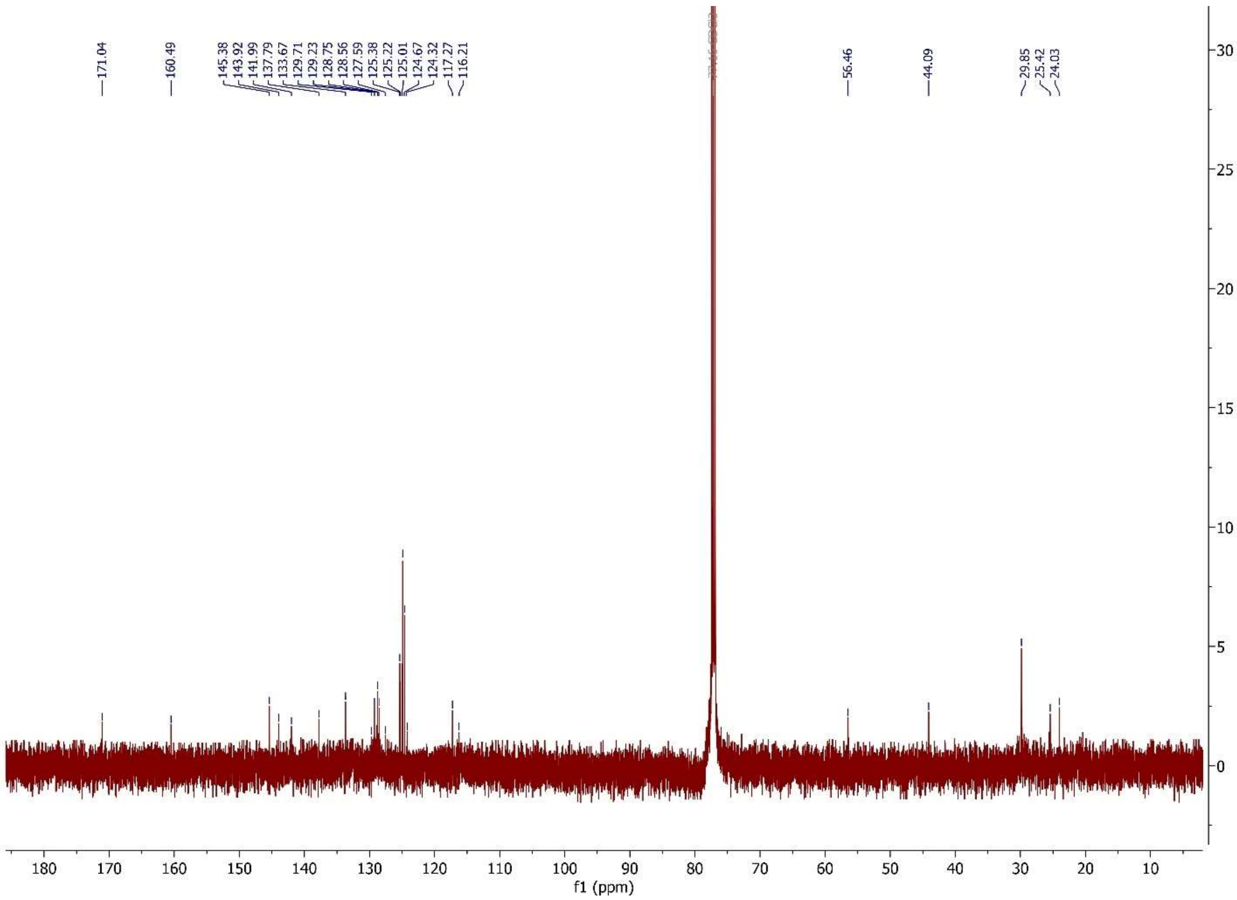

Figure S36: ^{13}C NMR spectrum of compound **17.**

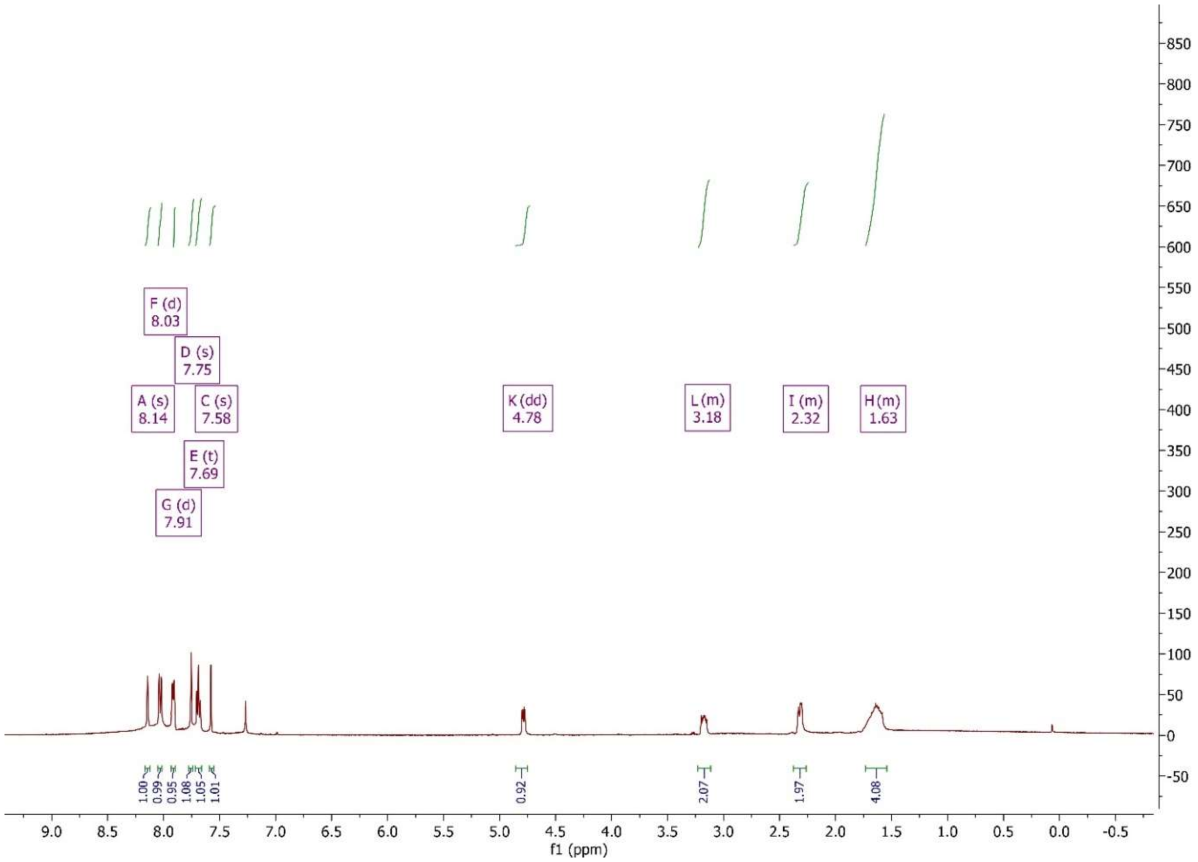

Figure S37: ^{1}H NMR spectrum of compound **18**.

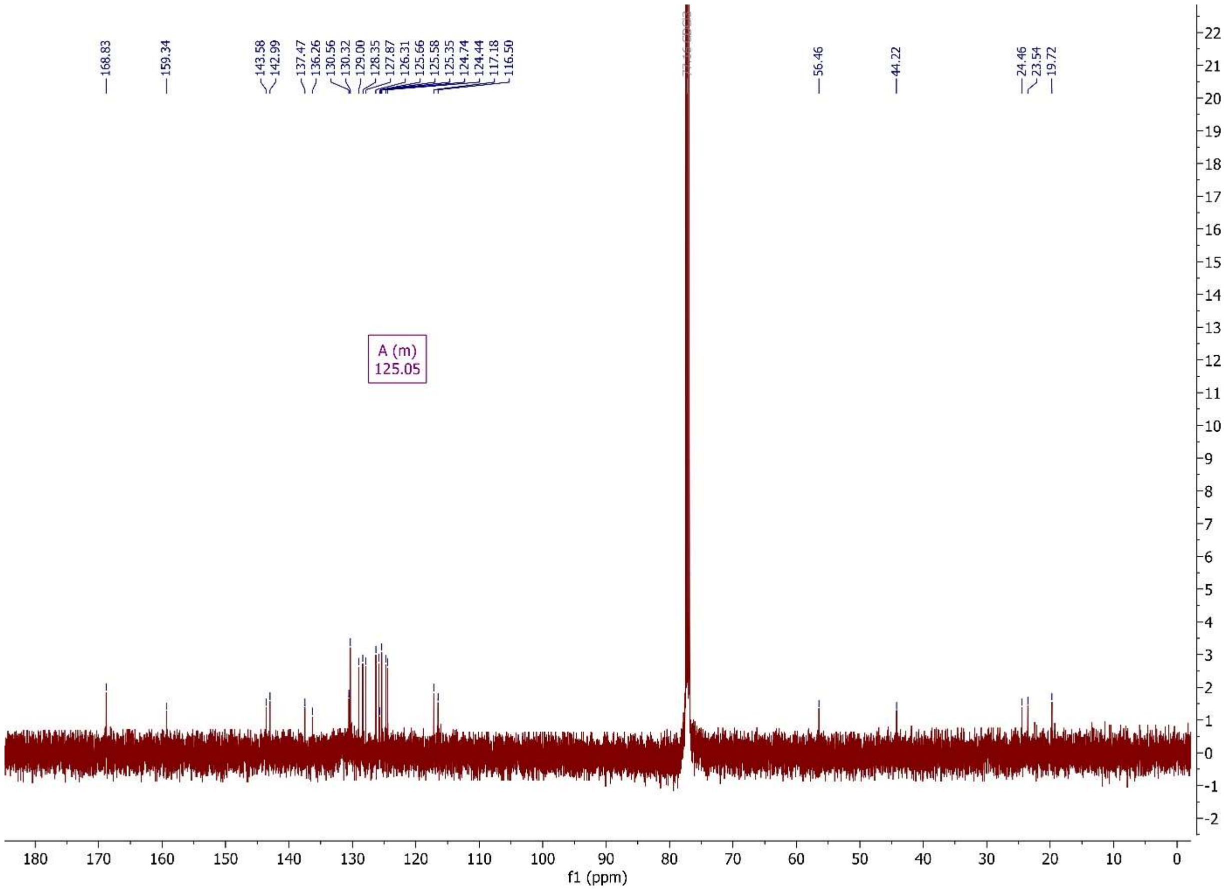

Figure S38: ^{13}C NMR spectrum of compound **18.**

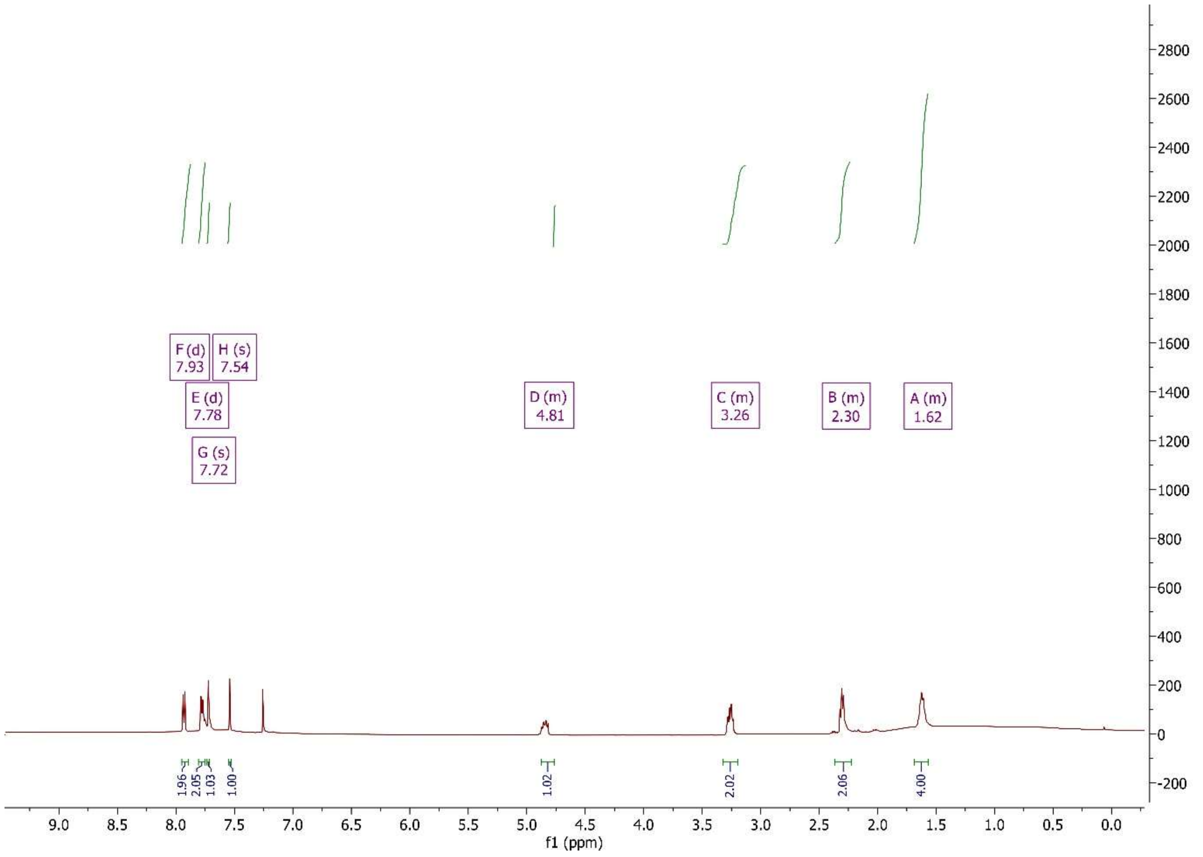

Figure S39: ^{1}H NMR spectrum of compound **19**.

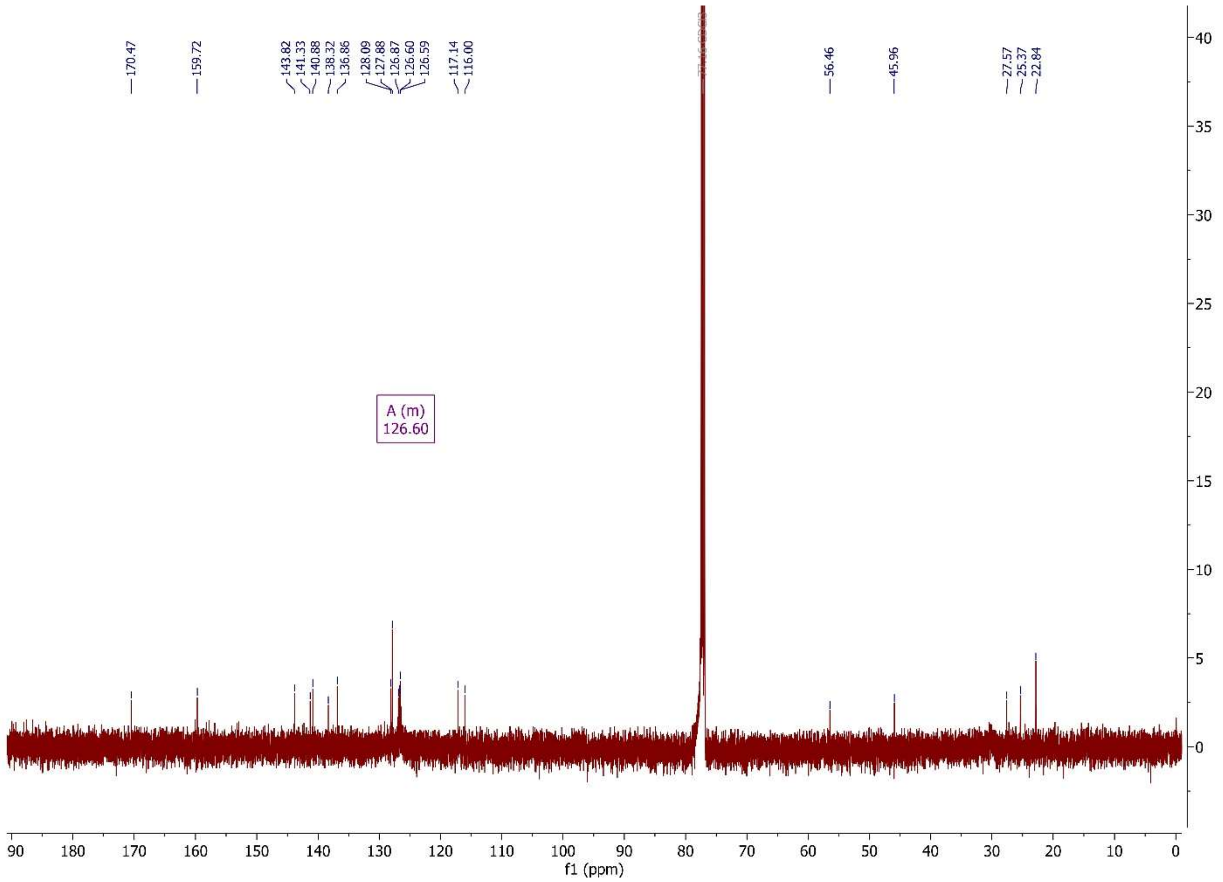

Figure S40: ^{13}C NMR spectrum of compound **19.**

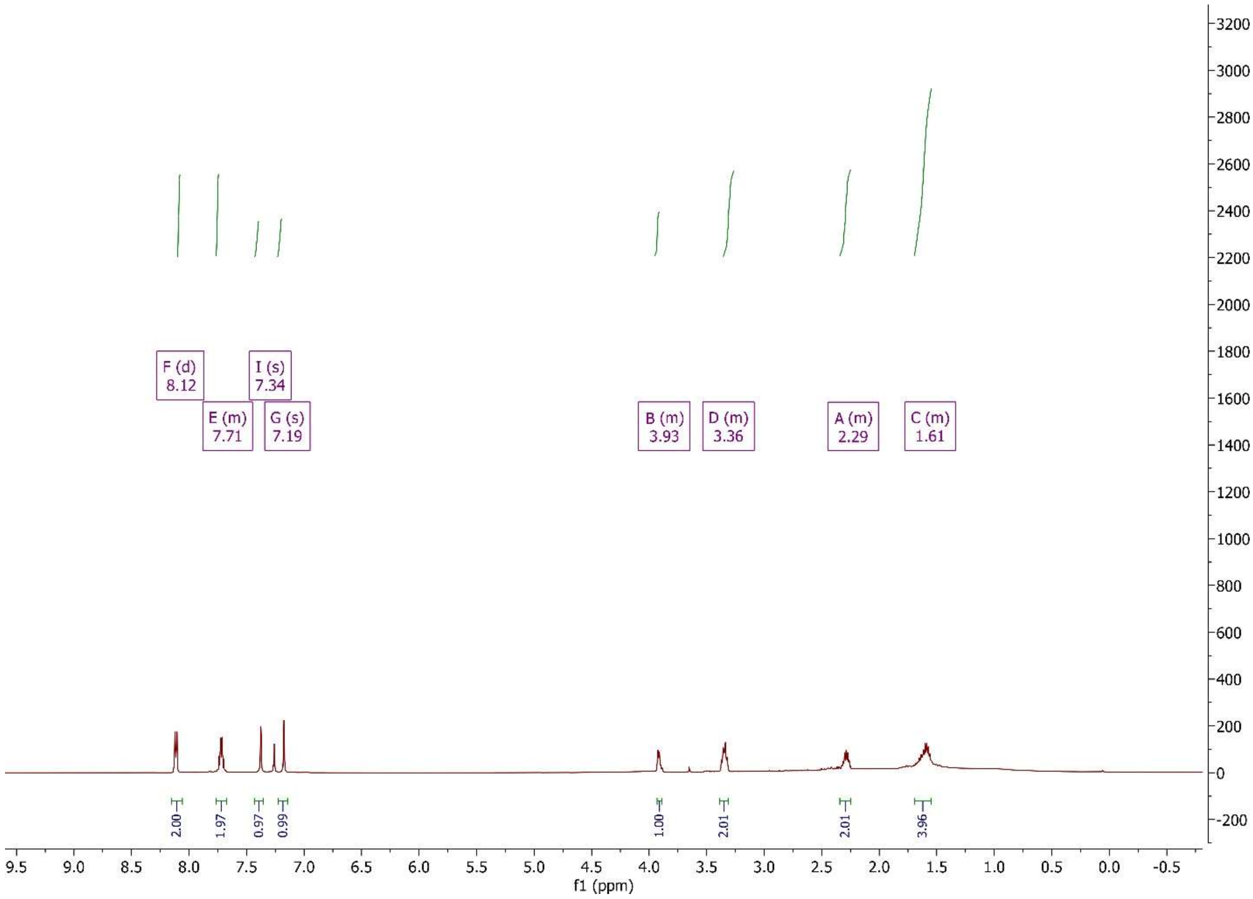

Figure S41: ^{1}H NMR spectrum of compound **20**.

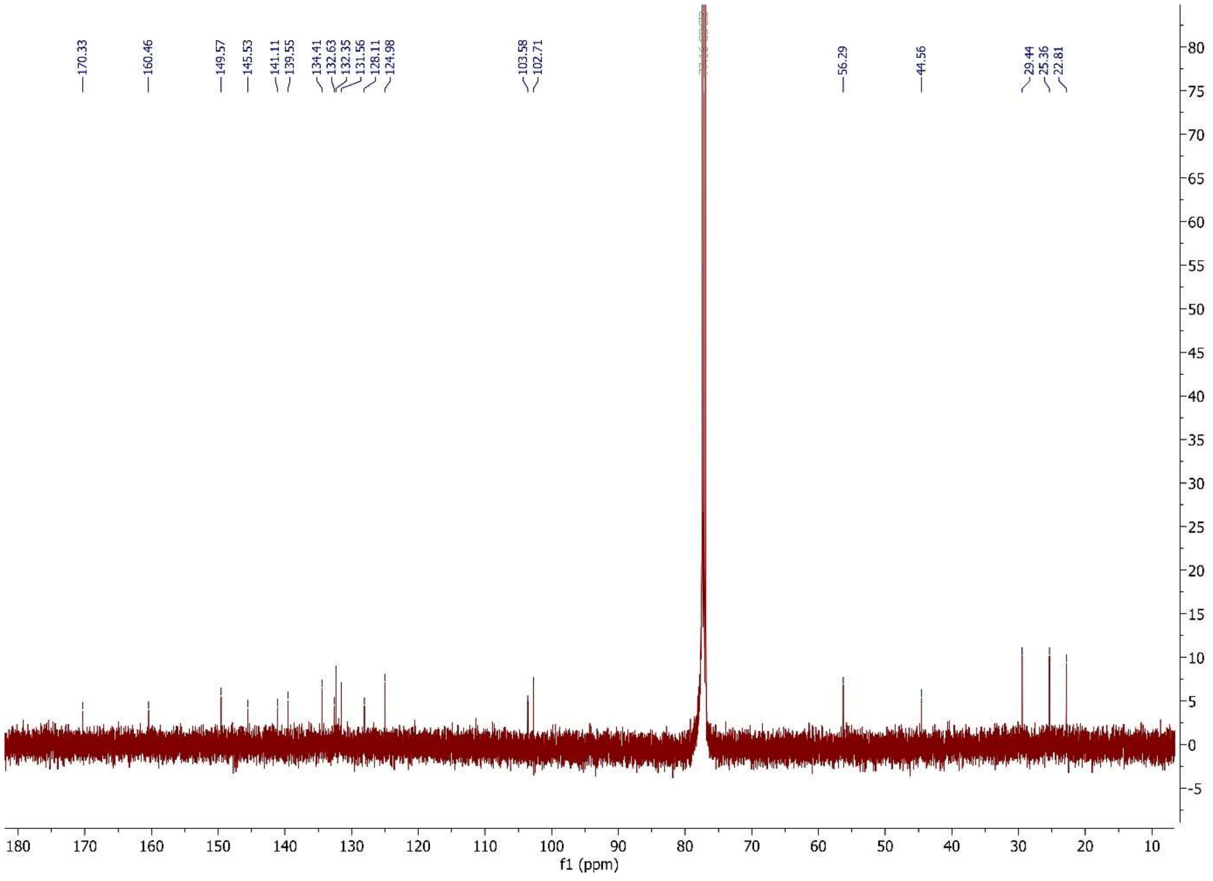

Figure S42: ^{13}C NMR spectrum of compound **20.**

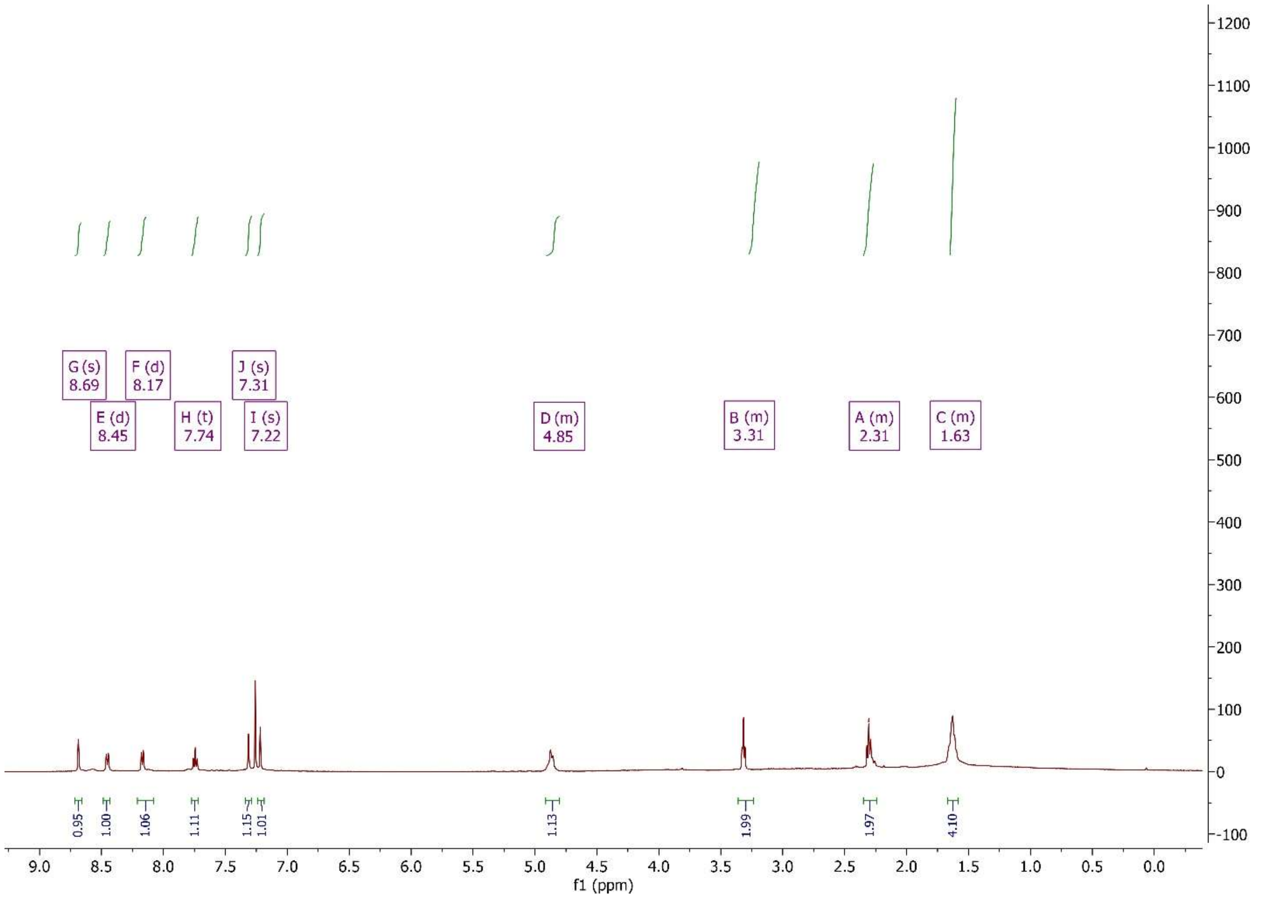

Figure S43: ^{1}H NMR spectrum of compound **21**.

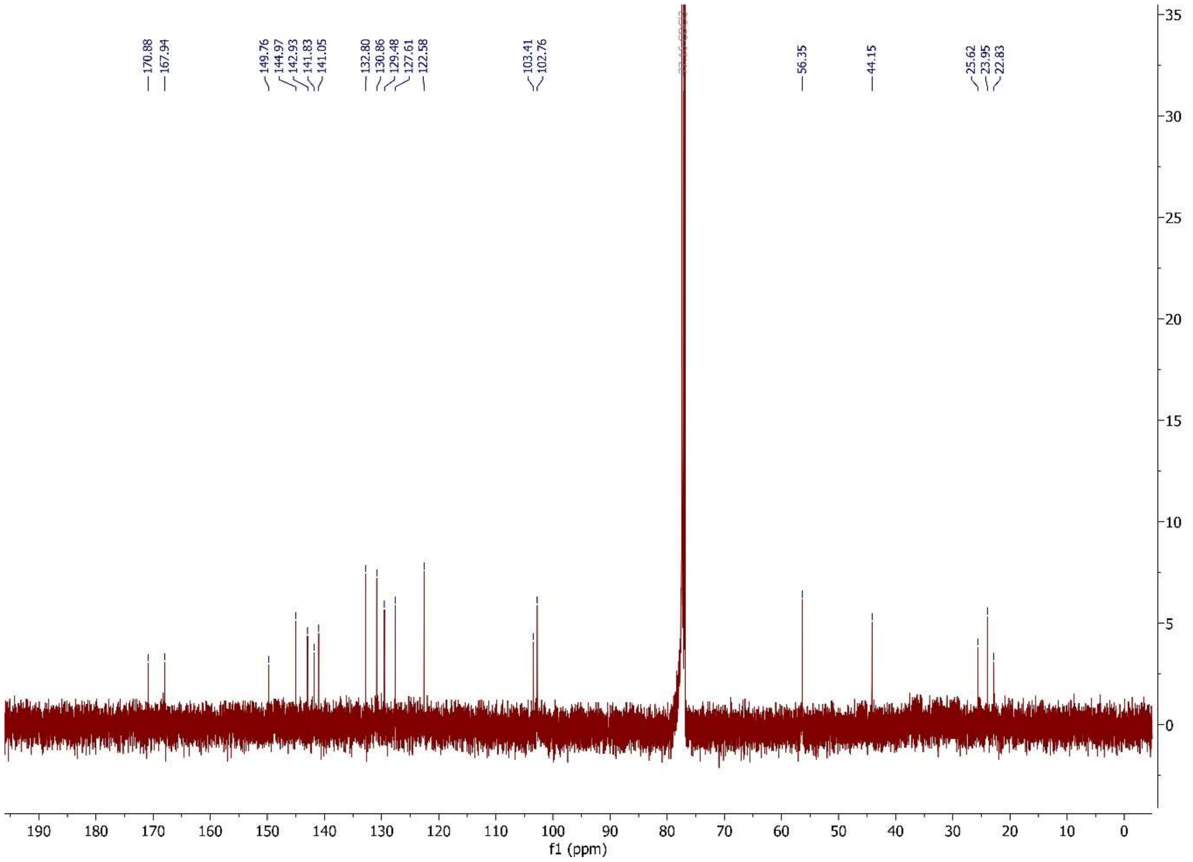

Figure S44: ^{13}C NMR spectrum of compound **21.**

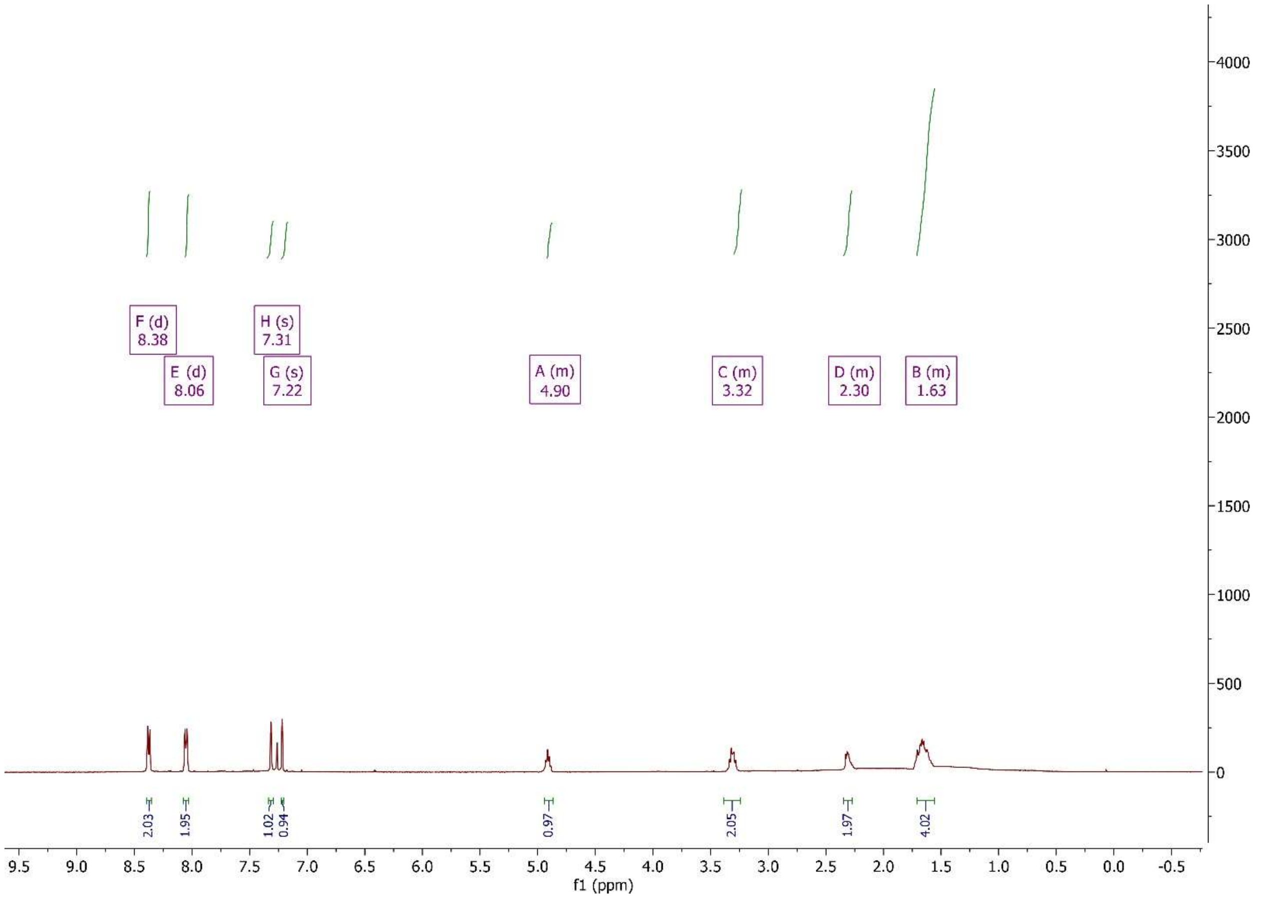

Figure S45: ^{1}H NMR spectrum of compound **22**.

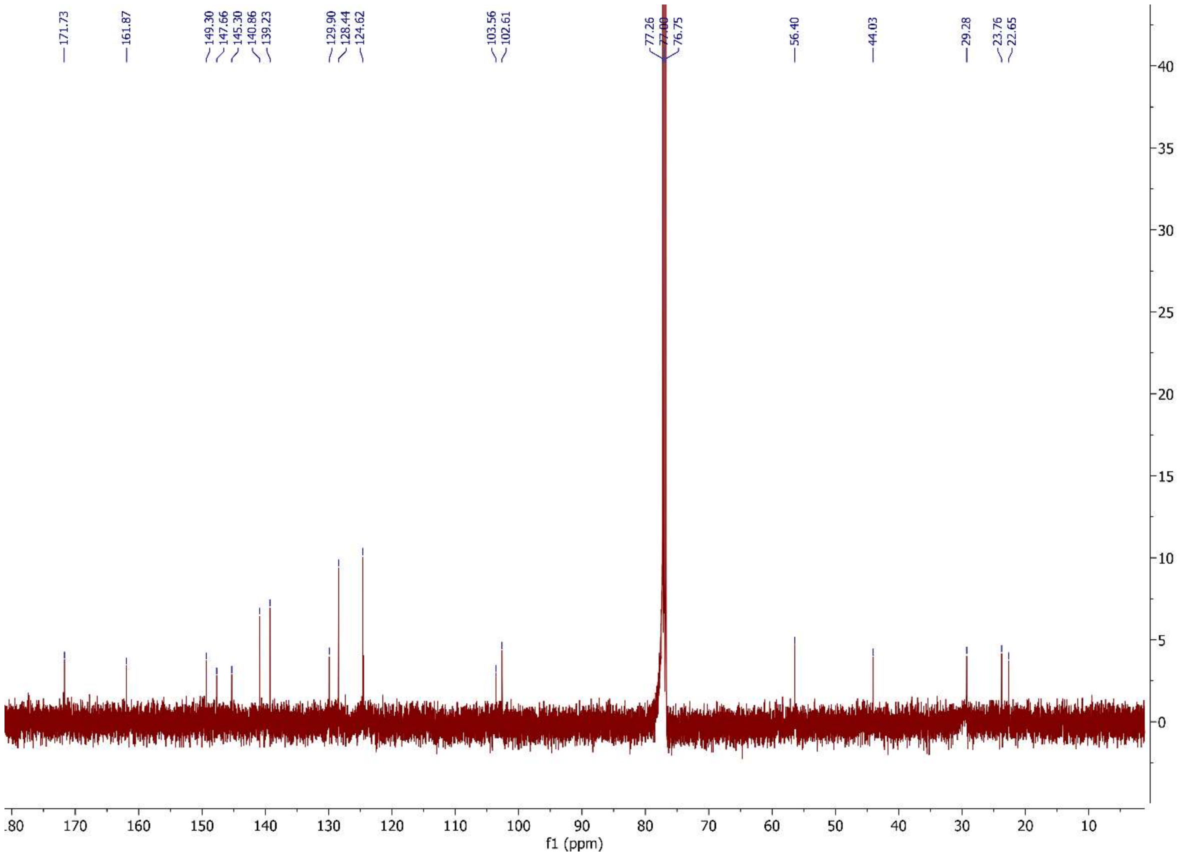

Figure S46: ^{13}C NMR spectrum of compound **22.**

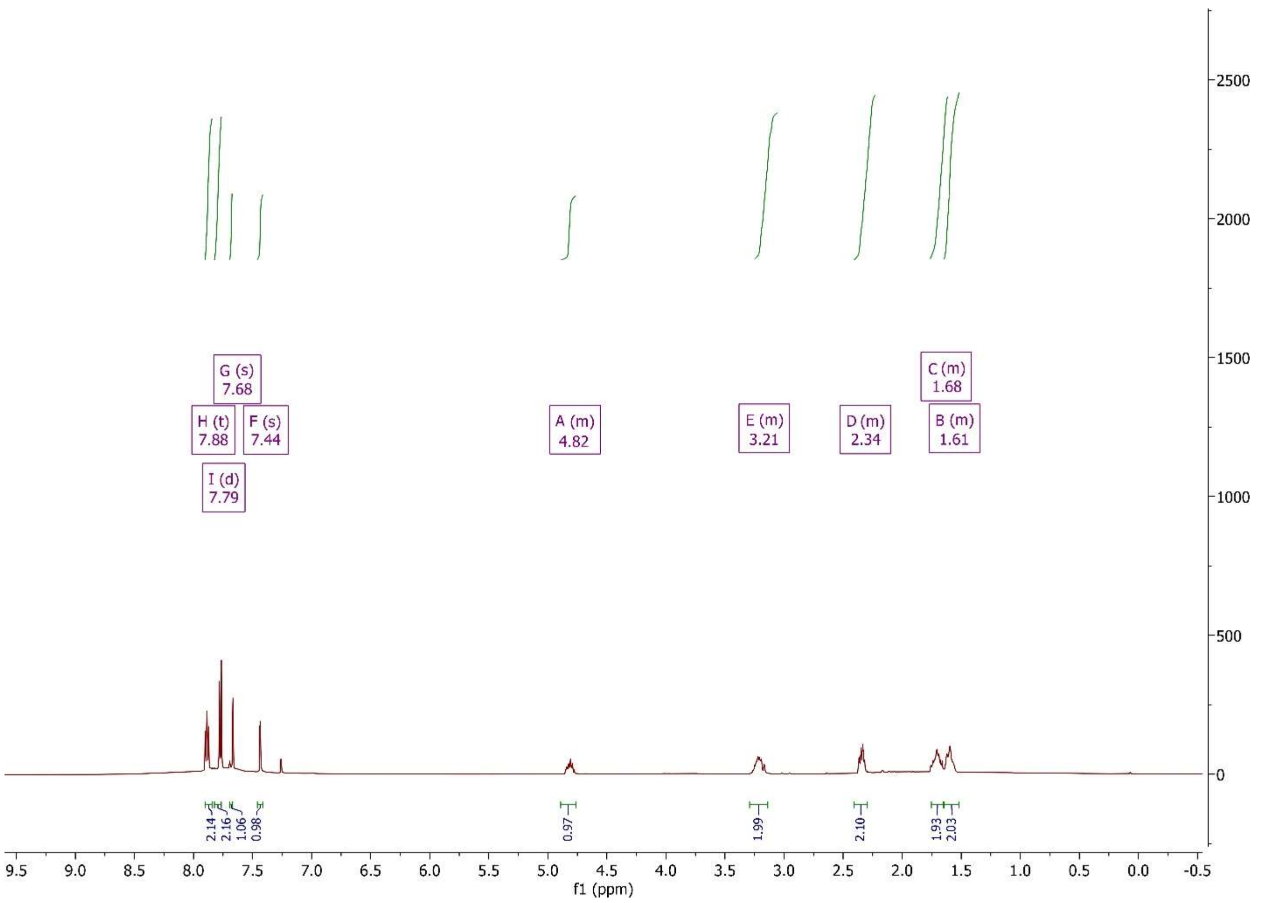

Figure S47: ^{1}H NMR spectrum of compound **23**.

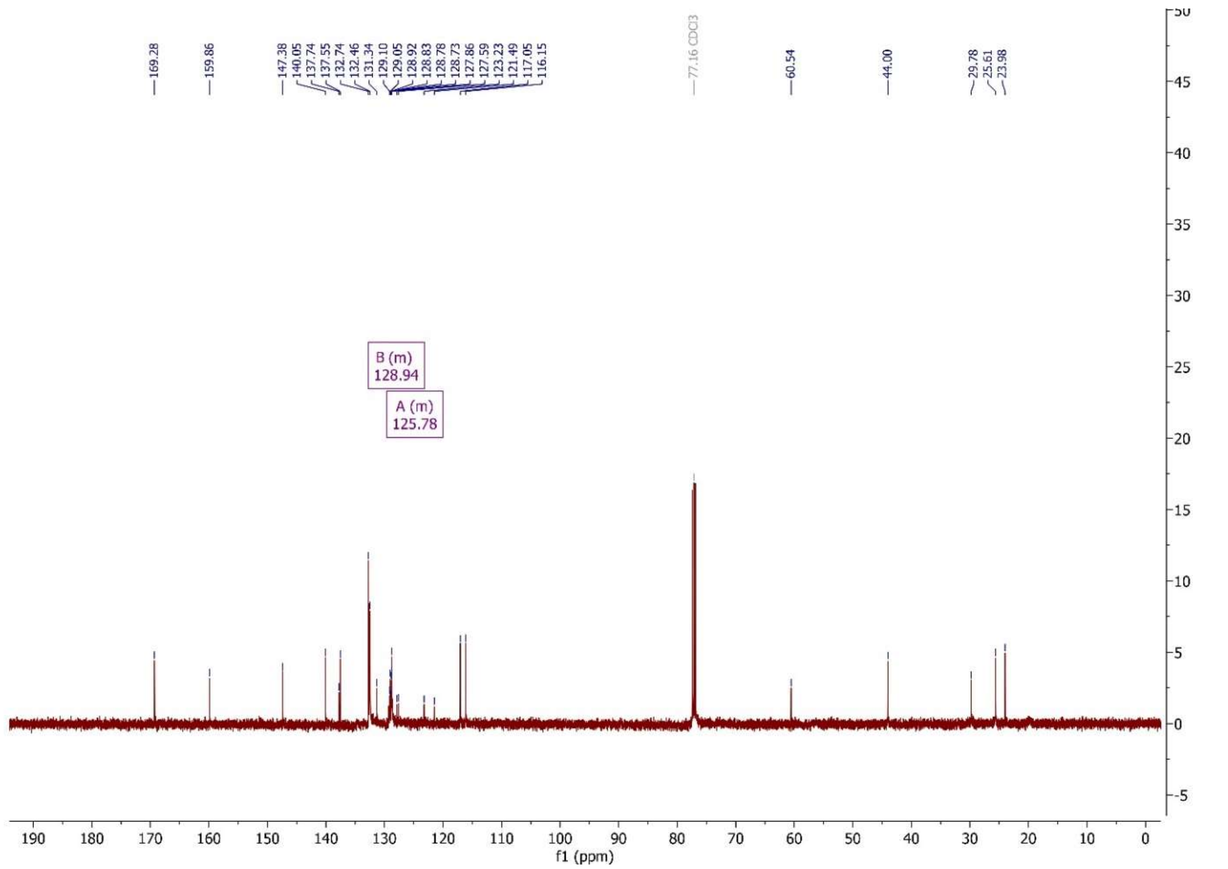

Figure S48: ^{13}C NMR spectrum of compound **23.**

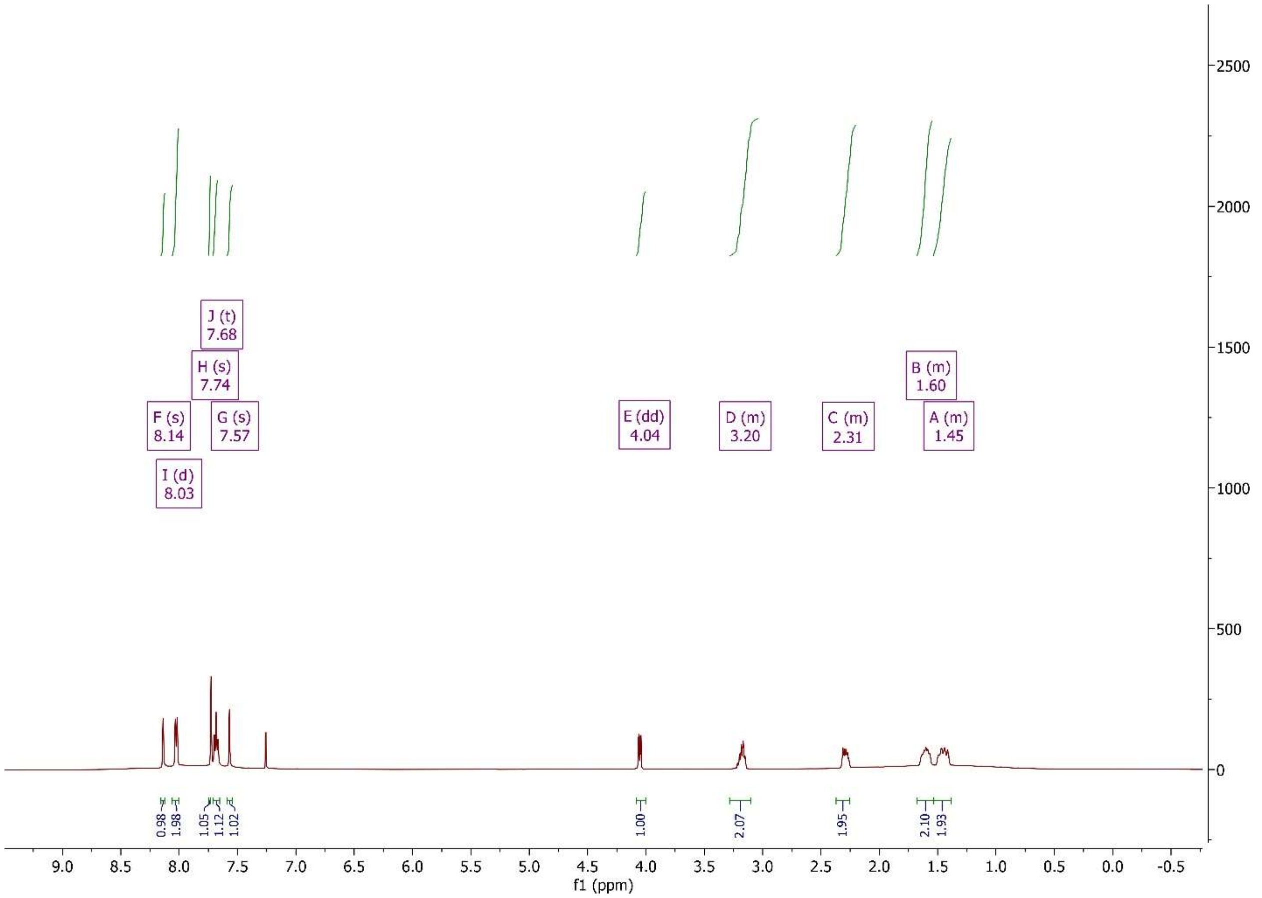

Figure S49: ^{1}H NMR spectrum of compound **24**.

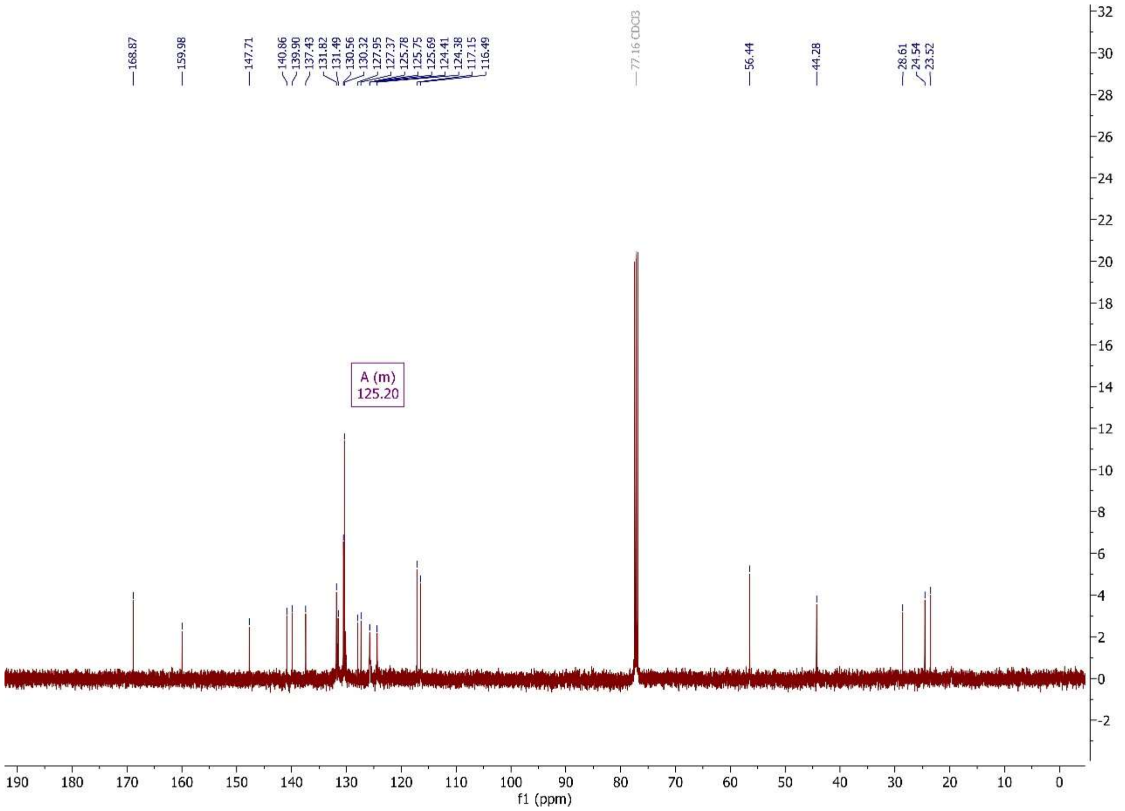

Figure S50: ^{13}C NMR spectrum of compound **24.**

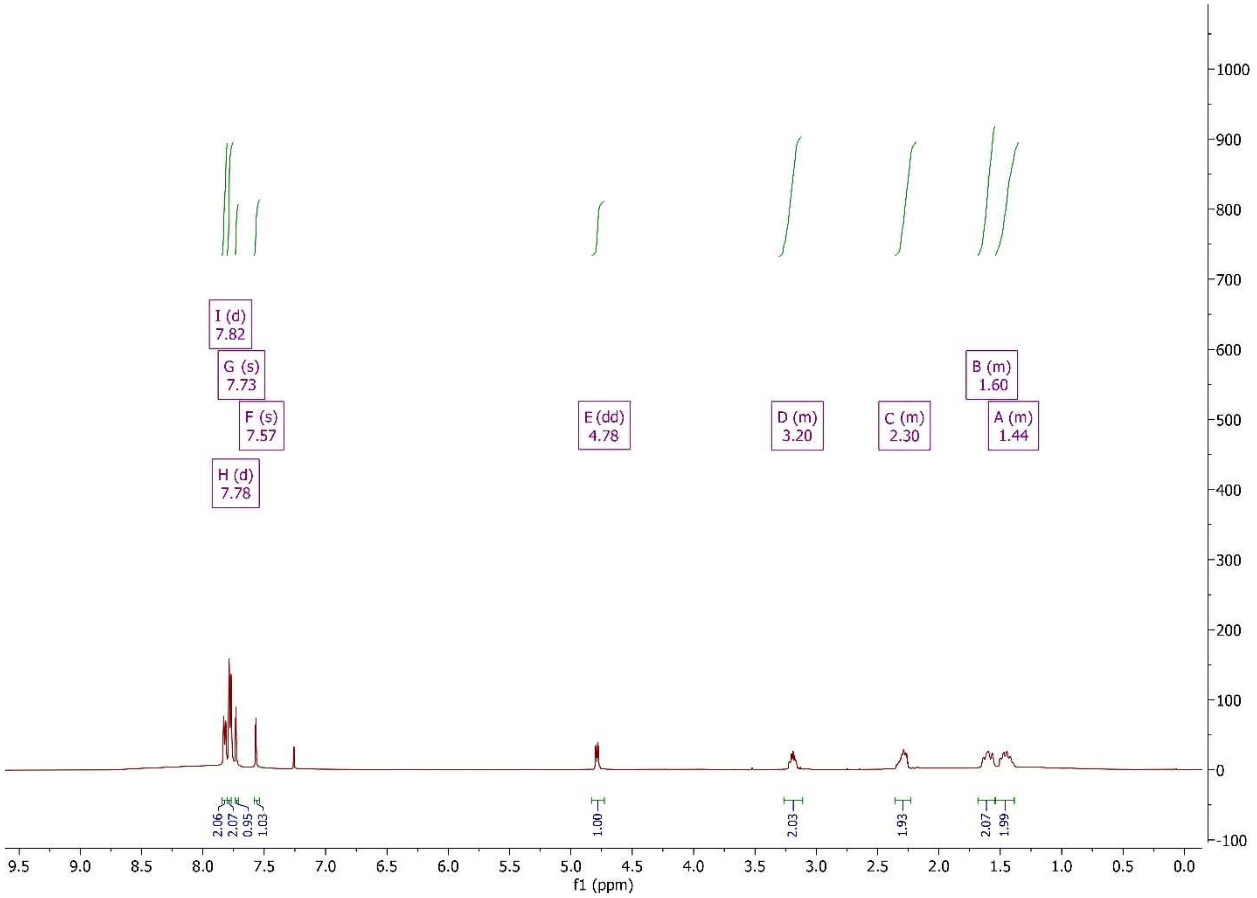

Figure S51: ^{1}H NMR spectrum of compound **25**.

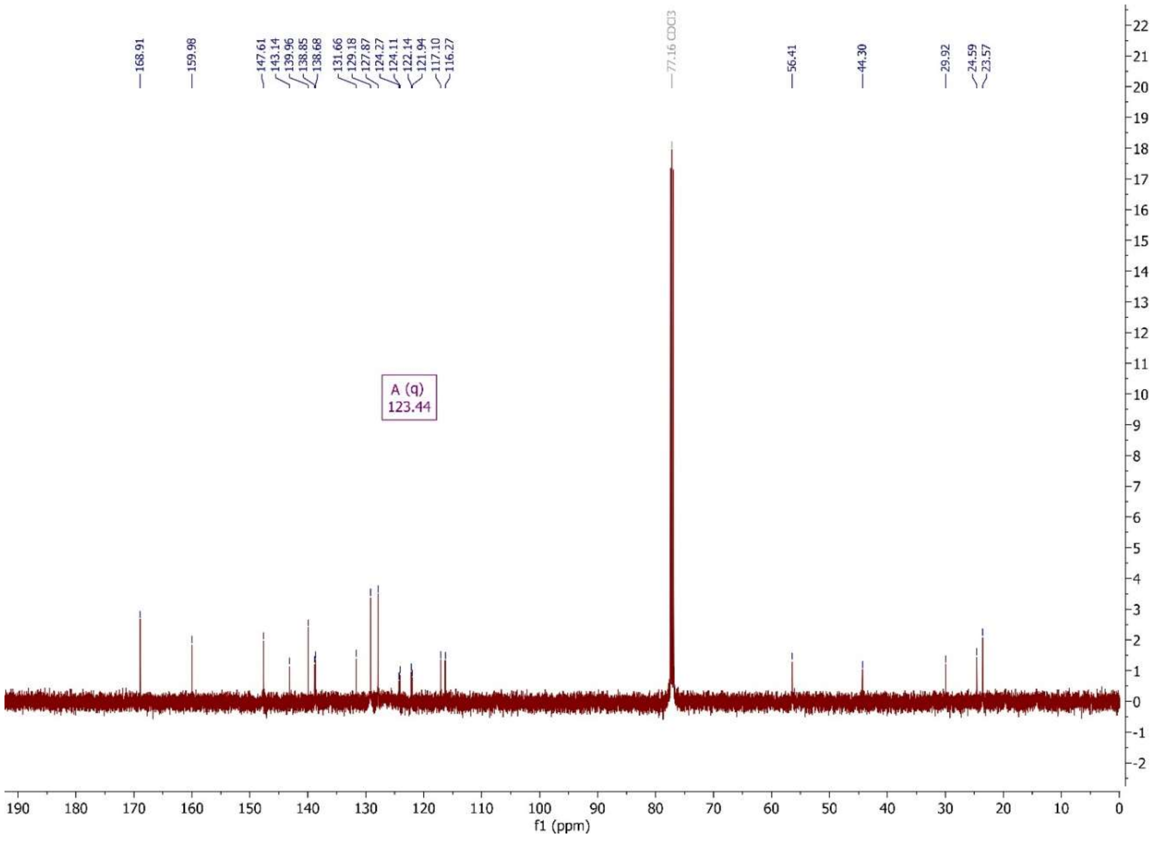

Figure S52: ^{13}C NMR spectrum of compound **25.**

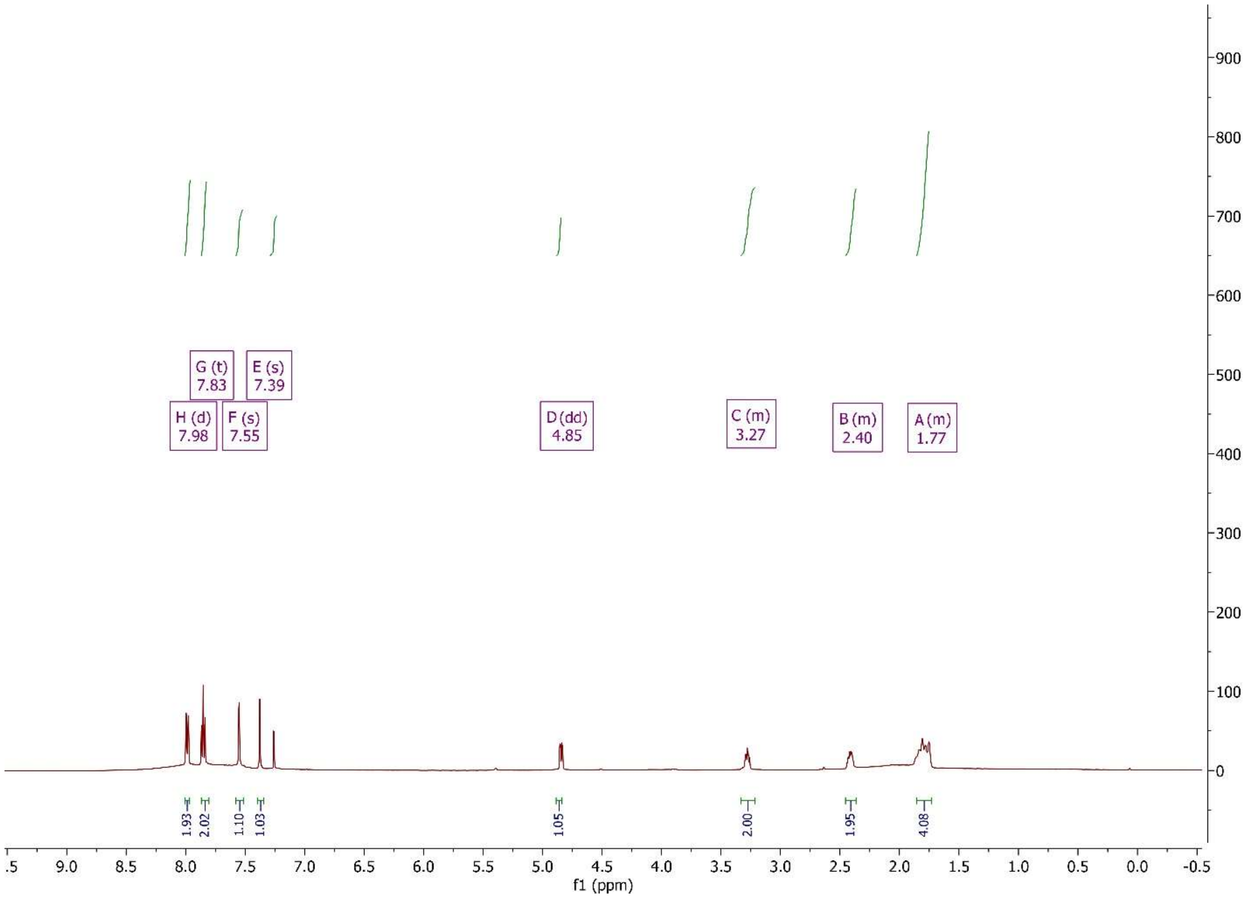

Figure S53: ^{1}H NMR spectrum of compound **26**.

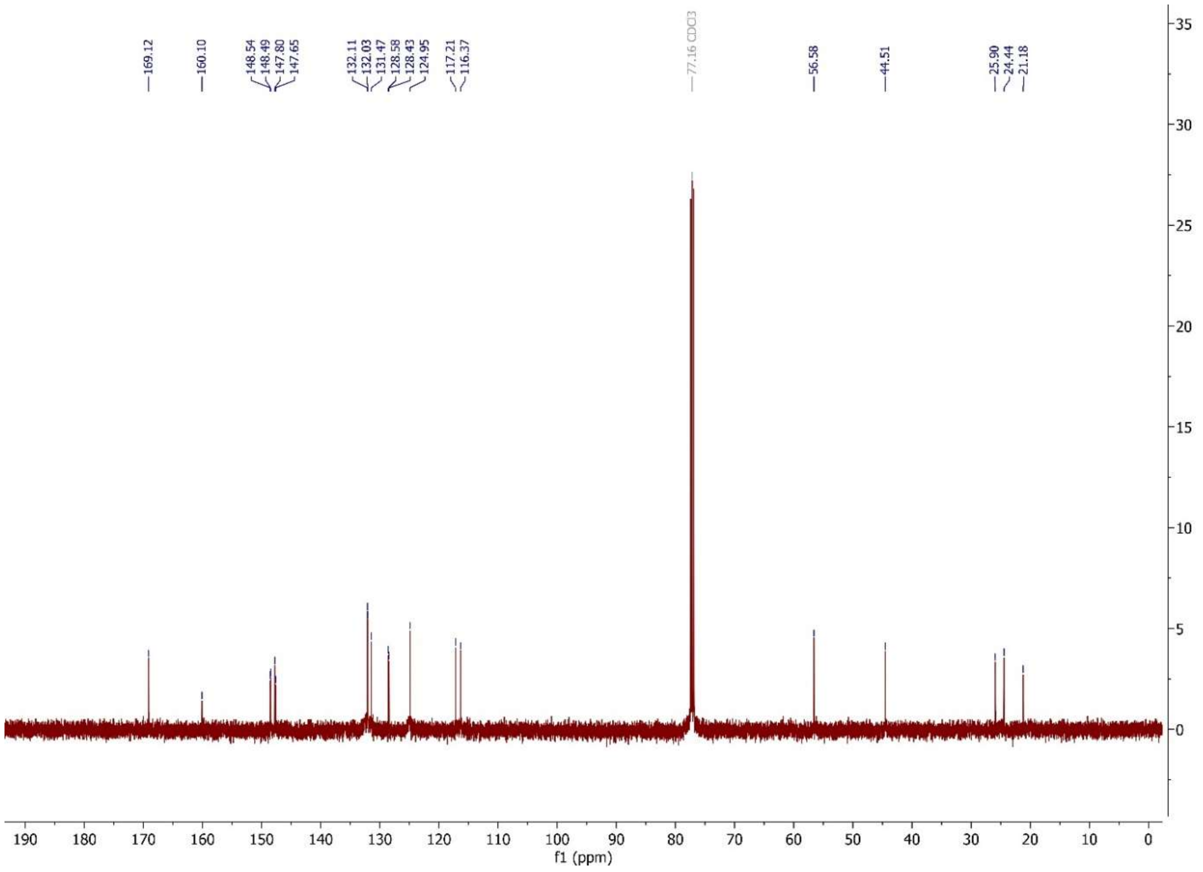

Figure S54: ^{13}C NMR spectrum of compound **26.**

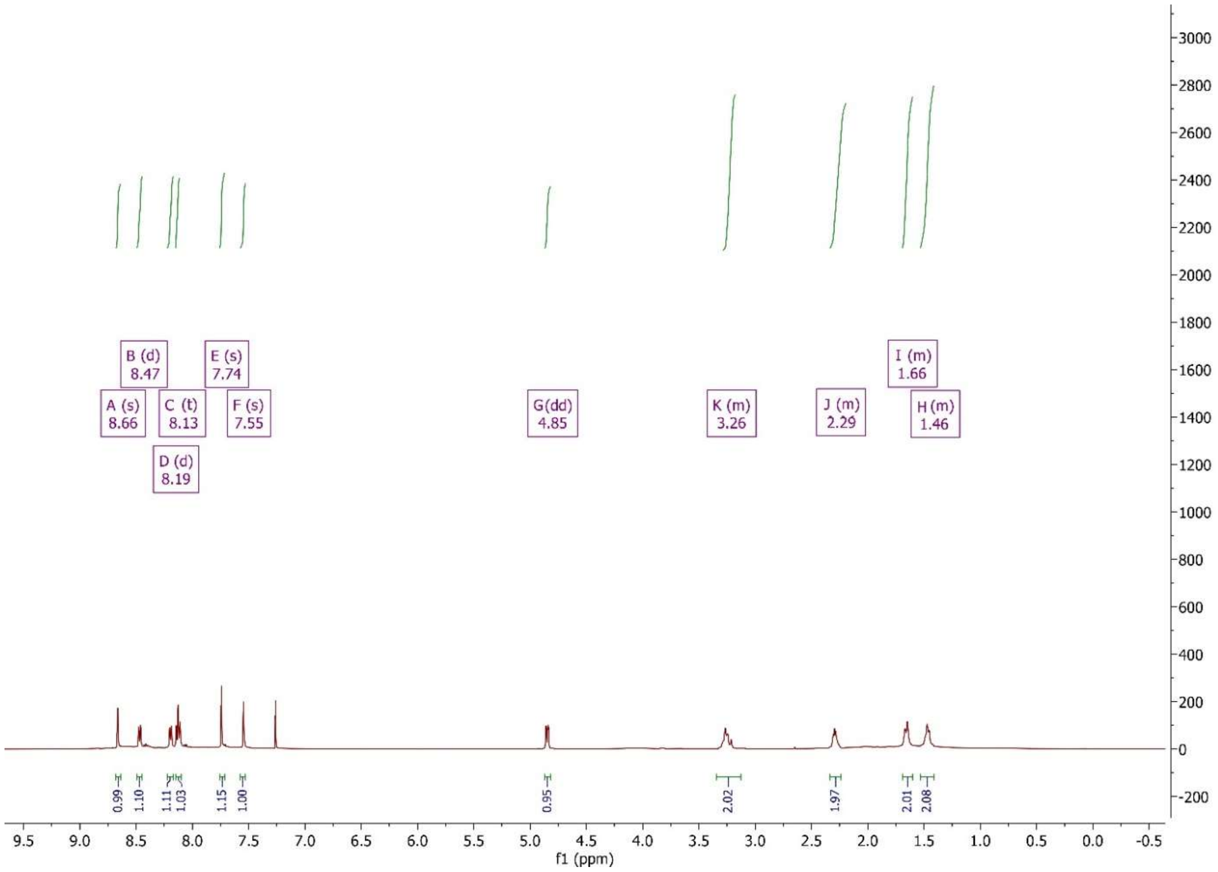

Figure S55: ^{1}H NMR spectrum of compound **27**.

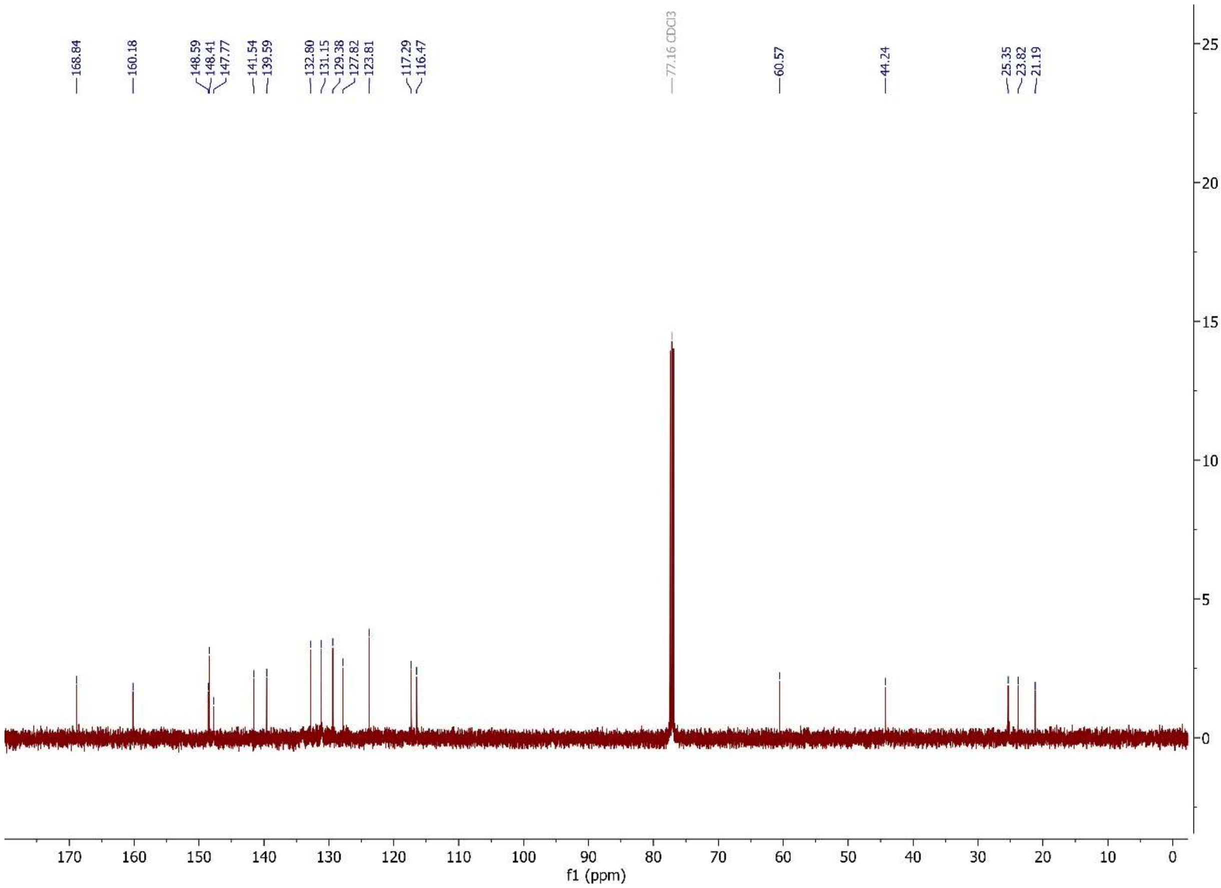

Figure S56: ^{13}C NMR spectrum of compound **27.**

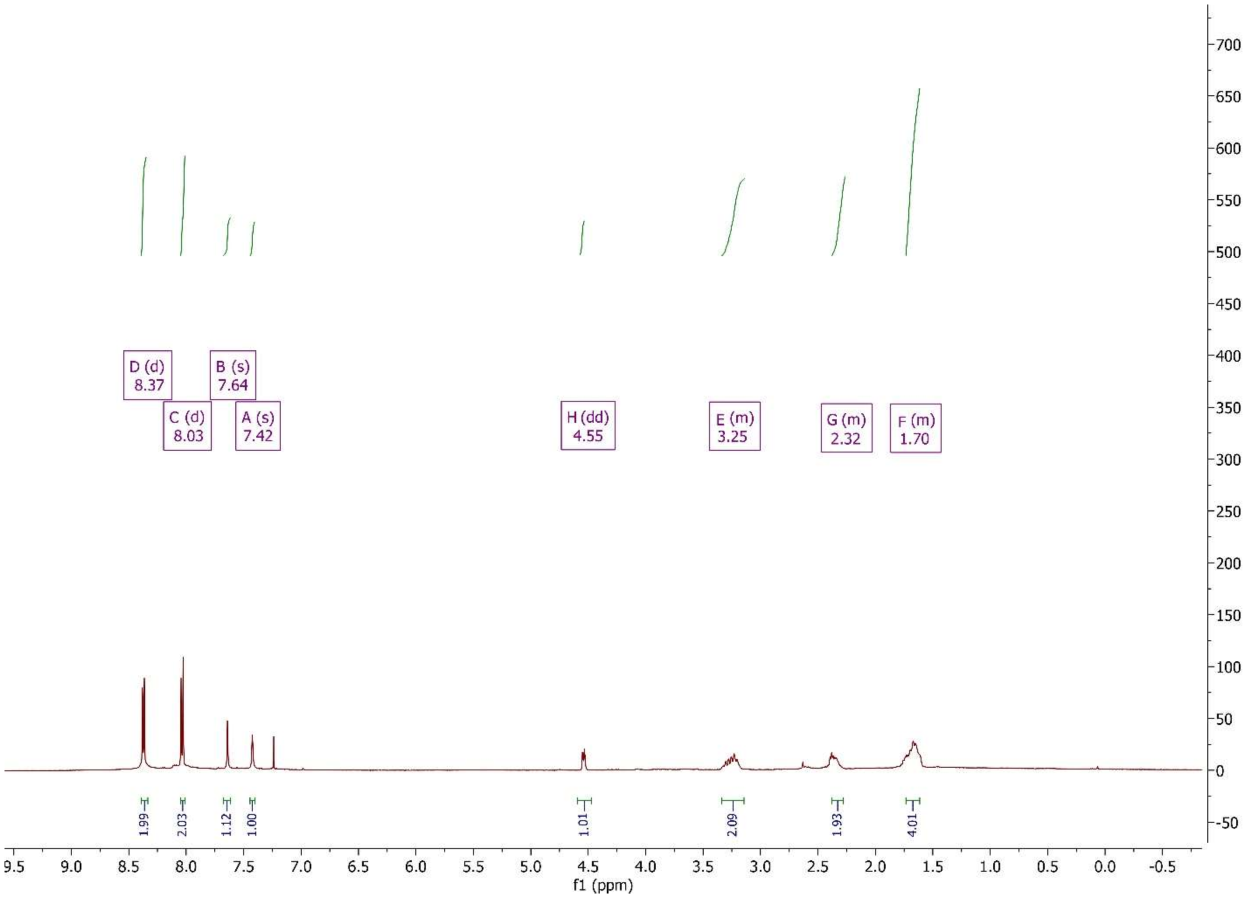

Figure S57: ^{1}H NMR spectrum of compound **28**.

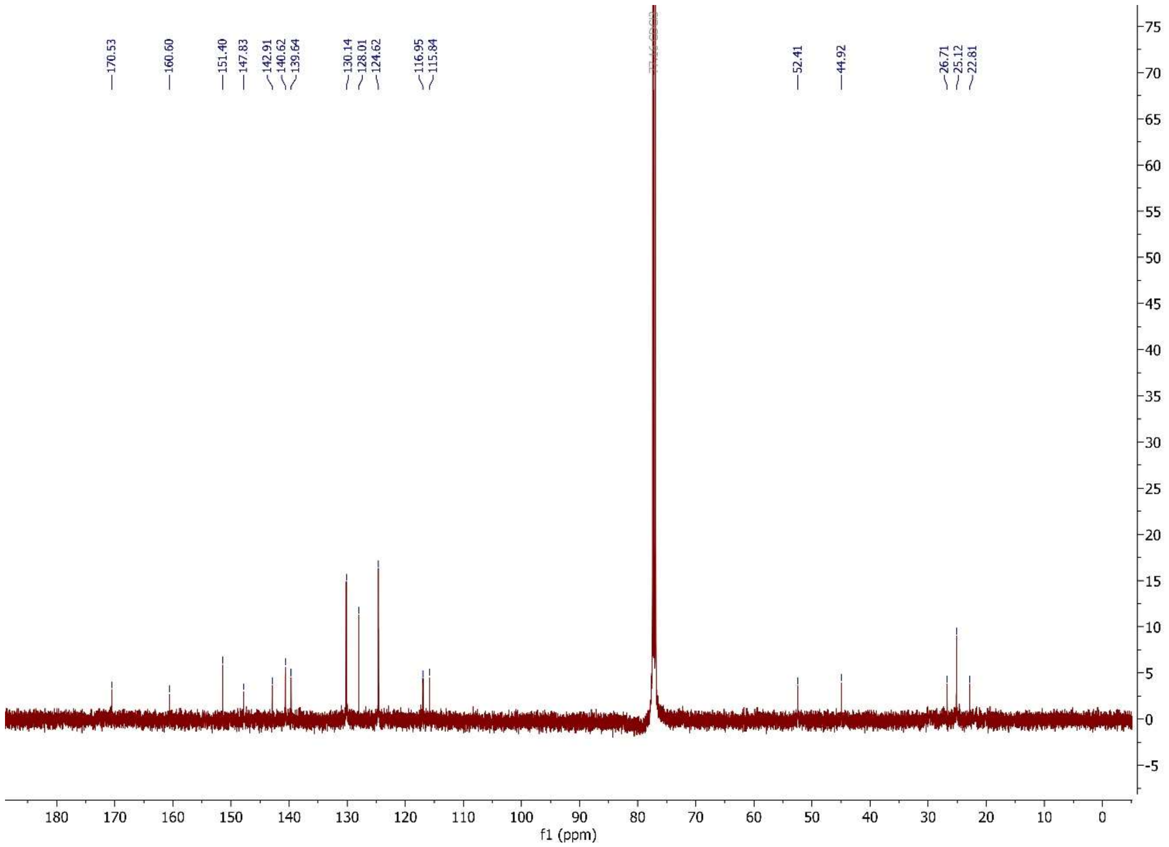

Figure S58: ^{13}C NMR spectrum of compound **28.**
